# Viral quasispecies inference from 454 pyrosequencing

**DOI:** 10.1186/1471-2105-14-355

**Published:** 2013-12-05

**Authors:** Wan-Ting Poh, Eryu Xia, Kwanrutai Chin-inmanu, Lai-Ping Wong, Anthony Youzhi Cheng, Prida Malasit, Prapat Suriyaphol, Yik-Ying Teo, Rick Twee-Hee Ong

**Affiliations:** 1 Saw Swee Hock School of Public Health, National University of Singapore, Singapore, Singapore; 2 Center for Emerging and Neglected Infectious Diseases, Mahidol University, Bangkok, Thailand; 3 Division of Bioinformatics and Data Management for Research, Office for Research and Development, Faculty of Medicine Siriraj Hospital, Mahidol University, Bangkok, Thailand; 4 Medical Biotechnology Research Unit, National Center for Genetic Engineering and Biotechnology, National Science and Technology Development Agency, Bangkok, Thailand; 5 Dengue Hemorrhagic Fever Research Unit, Office for Research and Development, Faculty of Medicine Siriraj Hospital, Mahidol University, Bangkok, Thailand; 6 Department of Statistics and Applied Probability, National University of Singapore, Singapore, Singapore; 7 NUS Graduate School for Integrative Science and Engineering, National University of Singapore, Singapore, Singapore; 8 Life Sciences Institute, National University of Singapore, Singapore, Singapore; 9 Genome Institute of Singapore, Agency for Science, Technology and Research, Singapore, Singapore; 10 Center for Infectious Disease Epidemiology and Research, National University of Singapore, Singapore, Singapore

**Keywords:** Virus quasispecies, Sequence alignment, 454 pyrosequencing

## Abstract

**Background:**

Many potentially life-threatening infectious viruses are highly mutable in nature. Characterizing the fittest variants within a quasispecies from infected patients is expected to allow unprecedented opportunities to investigate the relationship between quasispecies diversity and disease epidemiology. The advent of next-generation sequencing technologies has allowed the study of virus diversity with high-throughput sequencing, although these methods come with higher rates of errors which can artificially increase diversity.

**Results:**

Here we introduce a novel computational approach that incorporates base quality scores from next-generation sequencers for reconstructing viral genome sequences that simultaneously infers the number of variants within a quasispecies that are present. Comparisons on simulated and clinical data on dengue virus suggest that the novel approach provides a more accurate inference of the underlying number of variants within the quasispecies, which is vital for clinical efforts in mapping the within-host viral diversity. Sequence alignments generated by our approach are also found to exhibit lower rates of error.

**Conclusions:**

The ability to infer the viral quasispecies colony that is present within a human host provides the potential for a more accurate classification of the viral phenotype. Understanding the genomics of viruses will be relevant not just to studying how to control or even eradicate these viral infectious diseases, but also in learning about the innate protection in the human host against the viruses.

## Background

Virus populations exist as pools of non-identical but related members called quasispecies [1]. Quasispecies are associated with the error-prone replications, high mutation rates and short generation times of the evolutionary dynamics of viruses, generating the genetic diversity that allows the species to persist in their hosts [2]. Due to the genetically labile nature of such viruses, which include most RNA virus such as dengue virus (DENV) and HIV, they often develop resistance to vaccines and antiviral drugs very quickly. The fitness of a virus, defined as the ability for a given virus variant to tolerate environmental changes and to reproduce successfully, is often reflected in the frequency [3]. It is thus of interest to try to study and characterise the fittest variants of the quasispecies, which may lead to the development of more effective therapeutic treatments.

When compared to high-throughput next-generation sequencing (NGS) techniques, traditional Sanger capillary sequencing tends to be more time consuming and relatively more expensive per sequenced base. NGS techniques have been widely applied in de-novo sequencing, re-sequencing, metagenomics and intra-host characterization of infections pathogens. These techniques produce more sequencing fragments as compared to traditional Sanger sequencing, thus allowing more details with a much higher coverage. However, confounding results may be derived due to the PCR step, shorter read lengths and higher sequencing error rates of these sequencing fragments.

Here, we are interested in using NGS to re-sequence the virus genome to characterize the fittest variants within a quasispecies from infected patients. While the cost of NGS is falling rapidly, the shorter reads and higher sequencing error rates require more computationally intensive analyses in assembling the sequence reads and in distinguishing if each polymorphic site is a genuine biological variant or a sequencing error.

There are currently several methods for quasispecies assembly, of which the majority of these methods have been summarized in the extensive review by Beerenwinkel and Zagordi, which looked at the problems and challenges faced in deciphering viral populations through the use of “ultra-deep sequencing” and also compared several of the existing methods and their limitations [4]. The quasispecies assembly problem can be divided into four components: (a) pre-processing of low quality reads and mapping of the filtered reads to the respective reference genome; (b) distinguishing whether each polymorphic site is the result of sequencing errors or due to a genuine mutation; (c) reconstructing full haplotypes from filtered and corrected reads; and (d) calculating the frequencies and confidence scores for the constructed haplotypes. The existing methods typically provide solutions that address a combination of at most three components in the problem of quasispecies reconstruction, frequently missing out on the pre-processing step [5-11].

More recently, Prosperi and colleagues introduced a set of formulae for the combinatorial analysis of a quasispecies [12]. They also introduced a reconstruction algorithm based on combinations of multinomial distributions using amplicons. This was subsequently implemented into QuRe to analyze long reads through sliding windows with a Poisson error correction method [13]. Also, QColors was published for non-contiguous reads [14] and QuasiRecomb, which takes into consideration recombination events that may occur in DNA viruses and RNA viruses such as HIV [15].

Of the approaches for quasispecies reconstruction, five methods have accompanying software that have been made publicly available:

ShoRAH (http://www.bsse.ethz.ch/cbg/software/shorah) [8-10];

ViSpA (http://alla.cs.gsu.edu/~software/VISPA/vispa.html) [5,6];

PredictHaplo (http://bmda.cs.unibas.ch/HivHaploTyper/) [11]; QuRe (http://sourceforge.net/projects/qure/) [12,13]; and

QuasiRecomb (http://www.bsse.ethz.ch/cbg/software/quasirecomb) [15].

In this paper, we aim to: (1) introduce a novel method QuasQ for reconstructing the genome sequences of the quasispecies that appropriately incorporates the base quality scores of each sequenced fragment; (2) using the base quality scores as well as the frequencies of each sequenced fragment, to derive the likelihood scores which will be used to effectively reduce the number of false positive haplotypes in the quasispecies inference. To compare the performance of QuasQ with the updated versions of ShoRAH, ViSpA, QuRe and QuasiRecomb, we performed a series of simulations generated from reference DENV serotype 1 sequence data where we vary: (i) the frequency distributions of the simulated variants; (ii) the total number of simulated variants; and (iii) the overall coverage of the data. In addition, we attempt to reconstruct the quasispecies of subtype B type-1 human immunodeficiency virus (HIV-1) *pol* clones from a real experiment obtained from 454 GS FLX Titanium platform [16]. Through these, we showed that our approach, QuasQ, shows reasonable recall rate in inferring the number of true unique variants with few false positives and frequencies that lie close to that of the clinical data as compared to ShoRAH, ViSpA, QuRe and QuasiRecomb. Finally, we apply this algorithm to clinical datasets of isolated DENV consisting of all four serotypes, sequenced on the 454 Genome Sequencer FLX System machine. QuasQ and the simulated datasets are available for download at http://www.statgen.nus.edu.sg/~software/quasq.html.

## Results

### Simulations results

The performance of QuasQ in detecting true polymorphic sites is first measured based by: (i) how many true polymorphic sites have been detected by QuasQ (recall rate); and (ii) how many of the detected polymorphic sites are true polymorphic sites (precision). QuasQ reported recall rates of between 0.984 and 0.998 and a precision of between 0.982 and 0.998 in the detection of true polymorphic sites (Table 1).

**Table 1:** Summary of the average (and standard deviation) of the recall rates, i.e. the number of true polymorphic sites detected out of all true polymorphic sites, and precision, i.e. the proportion of true polymorphic sites detected with respect to the total number of polymorphic sites reported by QuasQ, in each of the eight scenarios

**Setting**	**Recall rate**	**Precision**
**A**	0.997	0.982
**B**	0.990	0.988
**C**	0.993	0.992
**D**	0.984	0.995
**E**	0.998	0.995
**F**	0.995	0.996
**G**	0.994	0.997
**H**	0.985	0.998

Also, the simulated data allows QuasQ to be compared against four out of five implemented and released softwares for quasispecies reconstruction; ShoRAH, ViSpA, QuRe and QuasiRecomb. Our simulation set-up varied the number of variants and their relative frequencies, the mutation rates per generation and overall coverage of the simulated reads, and this yielded eight scenarios (annotated A-H, see Table 2). We simulated 50 datasets in each of the eight scenarios. In assessing the performance of QuasQ, ShoRAH, ViSpA, QuRe and QuasiRecomb, we quantify the performance in terms of (i) how many of the simulated variants have been correctly inferred by the software (recall rate); (ii) how many unique simulated sequences are reported by the software (precision); (iii) the degree of genetic similarity between the reconstructed sequences and the simulated sequences (similarity); and (iv) how close the estimated frequencies of the constructed haplotypes are when compared to the simulated sequences.

**Table 2:** Summary of the eight settings used to simulate the data for comparing the performance of the different sequence alignment approaches

**Setting**	**# quasi-species**	**Mutation rate**	**Coverage**
**A**	10	0.02	750
**B**	15	0.02	750
**C**	10	0.05	750
**D**	15	0.05	750
**E**	10	0.02	1500
**F**	15	0.02	1500
**G**	10	0.05	1500
**H**	15	0.05	1500

However, in our analysis of the simulated data under scenario A with QuasiRecomb (assuming both options of with and without recombination), around 10,000 haplotypes were constructed during each round of simulations with a precision of between 0.0002-0.0004 and these accounted for only 10%-40% of the simulated variants (see Additional file 1: Table S1). As a result, we subsequently excluded it from the comparison against the other 4 methods.

We observed that QuasQ in general constructed comparable or less number of haplotypes than ShoRAH, ViSpA and ViSpA (corrected), while QuRe has the lowest number (Table 3). It should also be noted that ShoRAH, ViSpA and QuRe produced haplotypes of differing lengths from the simulations. Hence, we used BLASTn (*E Value* <0.001) to align and score the constructed sequences with respect to the actual simulated variants. The constructed haplotypes aligned to ≥80% of the length of the reference genome with the highest identity to that simulated variant is considered (see Additional file 1 for example). However, we find that majority of the haplotypes constructed by ViSpA aligns to <80% of the length of any simulated variants. Hence ViSpA was similarly excluded in the assessment of recall rates, precision and F-measure.

**Table 3:** **Summary of the mean and standard deviation of the total number of constructed haplotypes by QuasQ, ShoRAH, QuRe, ViSpA and ViSpA (with corrected reads) over 50 runs across each of the eight scenarios as described in Table**2

		**Mean number of constructed sequences (SD)**				
**Setting**	**Simulated #**	**QuasQ**	**ShoRAH**	**QuRe**	**ViSpA**	**ViSpA (Corrected)**
**A**	10	194.7 (139.5)	267.8 (52.3)	84.5 (21.2)	7,524.0 (297.6)	166.0 (37.5)
**B**	15	435.0 (207.2)	298.0 (47.8)	103.7 (29.6)	8,257.4 (223.6)	240.5 (48.6)
**C**	10	189.1 (115.6)	468.0 (122.7)	67.3 (17.8)	8,744.2 (355.1)	409.1 (77.2)
**D**	15	315.2 (172.8)	671.2 (124.6)	90.0 (18.7)	9,367.3 (434.1)	563.3 (115.8)
**E**	10	382.8 (186.3)	290.2 (62.7)	128.7 (23.4)	14,721.6 (451.4)	213.1 (48.7)
**F**	15	506.4 (133.5)	419.6 (97.6)	164.3 (44.7)	16,064.6 (629.5)	344.0 (92.3)
**G**	10	449.8 (206.9)	786.3 (184.2)	125.4 (29.0)	16,373.3 (1,127.6)	629.5 (111.9)
**H**	15	534.7 (143.2)	1,155.1 (244.0)	150.7 (34.7)	17,040.8 (2,682.2)	917.8 (207.1)

**Figure 1:**
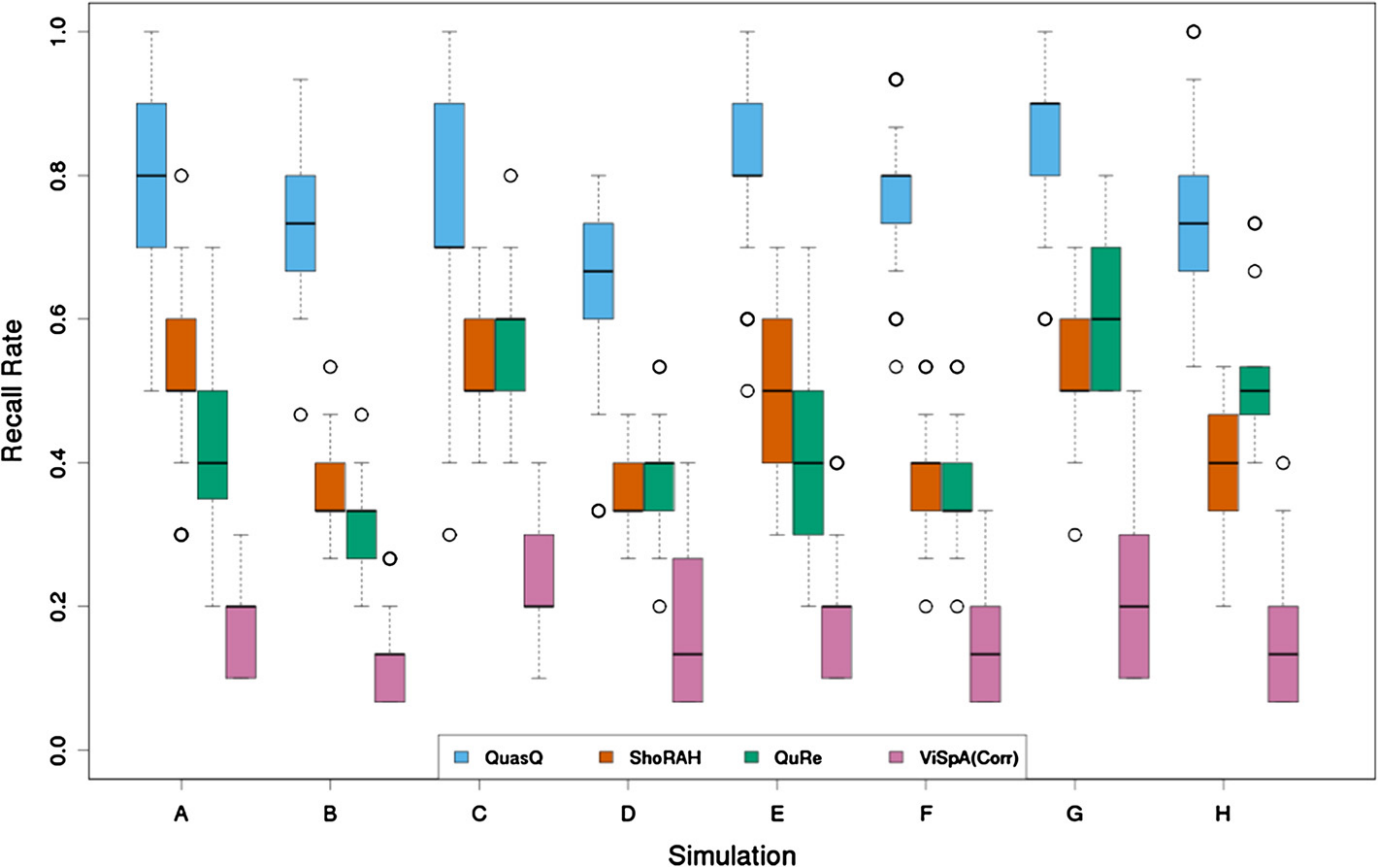
**Recall rate comparison of QuasQ and existing methods for simulations.** Boxplot of the recall rate, i.e. the number of unique simulated variants detected out of the 10 or 15 simulated variants, for 50 simulation runs across each of the eight settings (A-H) as described in Table 2 for QuasQ (blue), ShoRAH (red), QuRe (green) and ViSpA with ShoRAH corrected reads (pink).

We observed that QuasQ has a relatively higher recall rate as compared to ShoRAH, QuRe and ViSpA (corrected), thus detecting more true variants than the other softwares (Figure 1). It is also noted that the constructed sequences from the four softwares typically account for only between 60% to 80% of the simulated variants.

**Figure 2:**
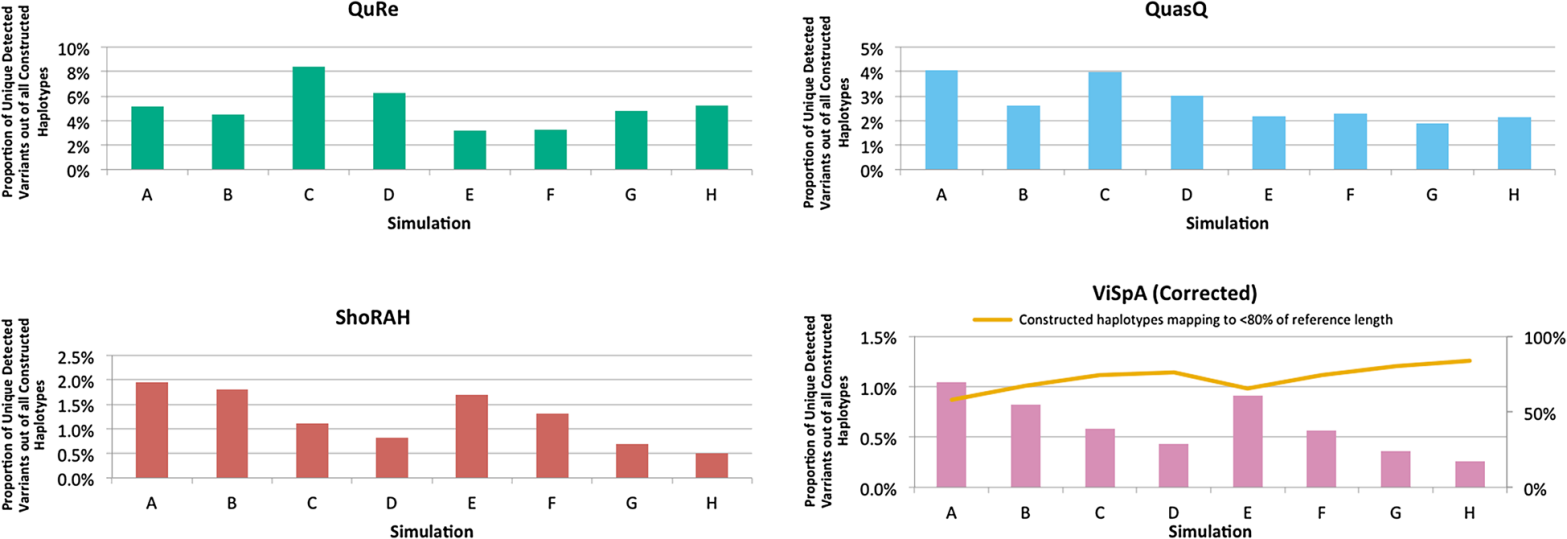
**Precision comparison of QuasQ and existing methods for simulations.** The vertical axes represent the average proportion of unique simulated variants detected with respect to the total number of constructed haplotypes by the corresponding method. Bars in blue indicate the average proportion of the constructed haplotypes that map to the simulated variants, whereas bars in red indicate that the average proportion of the constructed haplotypes that are mapped to the repeated same simulated variants. Both the red and blue bars consider haplotypes that are mapped to at least 80% of the length of the respective reference sequence. For QuasQ, ShoRAH and QuRe, all constructed haplotypes map to at least 80% of the length of the respective reference sequence. For ViSpA with ShoRAH corrected reads, the green line which is described by the vertical axis on the right, shows the proportion of haplotypes that do not map to at least 80% of the length of the reference sequence out of all the constructed haplotypes. These proportions are average of the 50 simulation runs across each of the eight settings (A-H) as described in Table 2.

**Figure 3:**
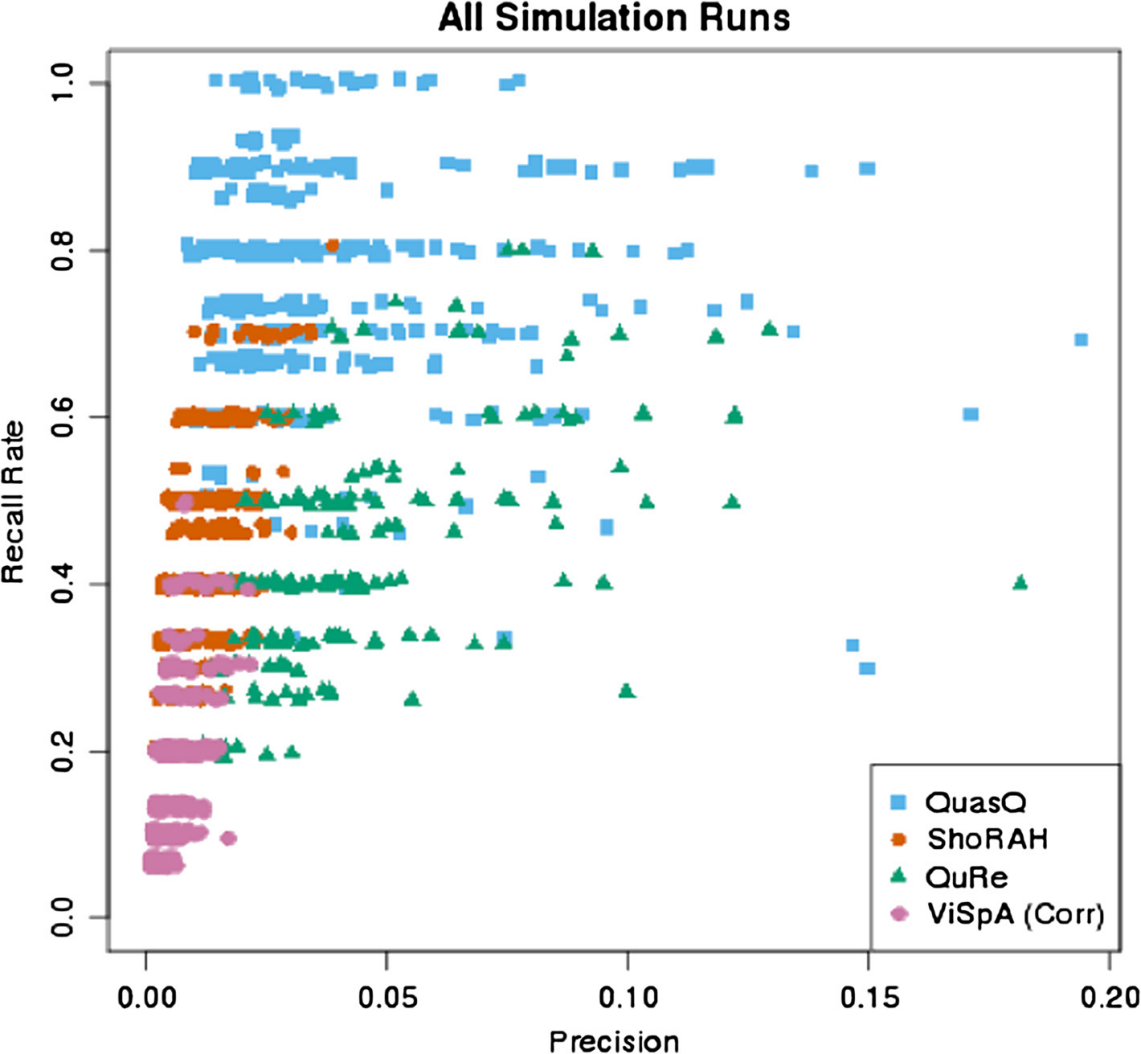
**Performance comparison of QuasQ and existing methods for all simulation runs.** Precision, defined as the proportion of unique simulated variants detected with respect to the total number of constructed haplotypes, versus the recall rate, defined as the number of unique simulated variants detected out of the 10 or 15 simulated variants, for QuasQ (blue squares), ShoRAH (red diamonds), QuRe (green triangles) and ViSpA with ShoRAH corrected reads (pink circle) in reconstructing the quasispecies over 50 runs across each of the eight settings (A-H) as described in Table 2.

Another metric that is relevant in comparing the performance between the softwares is in addressing the precision of each method: how many of the simulated variant sequences have been successfully reconstructed, out of the total number of reconstructed sequences. We observed that QuasQ tends to exhibit a higher precision compared to ShoRAH and ViSpA (corrected), although it is apparent that a significant proportion of the sequences that are inferred by all the methods do not actually match any of the simulated variant sequences (Figure 2). QuRe on the other hand displayed a much higher precision than the rest of the software due to the conservative reconstruction algorithm that produces lesser number of constructed sequences. Figure 3 shows the recall rate against precision across all the simulated runs (see Additional file 1: Figure S3 for detailed breakdown).

A more meaningful interpretation is to calculate the F-measure of the recall metric and precision metric, defined as the harmonic mean calculated as 2(recall rate × precision)/(recall rate + precision), as this will evaluate how many of the simulated sequences have been successfully identified that are accompanied by the least number of irrelevant reconstructed sequences. Here, QuasQ outperformed ShoRAH and ViSpA (corrected) in most simulations but ranks slightly below QuRe due to QuRe’s conservative nature (Table 4).

**Table 4:** **Summary of the mean F-measure for QuasQ, ShoRAH, QuRe and ViSpA (with ShoRAH corrected reads) over 50 runs across each of the eight scenarios as described in Table**2

	**F-measure (Mean)**			
**Setting**	**QuasQ**	**ShoRAH**	**QuRe**	**ViSpA (Corr)**
**A**	0.108	0.039	0.096	0.020
**B**	0.065	0.035	0.085	0.016
**C**	0.100	0.024	0.153	0.019
**D**	0.080	0.017	0.110	0.008
**E**	0.055	0.035	0.061	0.018
**F**	0.048	0.027	0.064	0.011
**G**	0.047	0.015	0.094	0.007
**H**	0.044	0.011	0.099	0.005

The constructed haplotype sequences are compared against each of the 10 or 15 simulated sequences, and we identified the constructed sequence that has the highest match to each of the simulated sequences and calculate the extent of base identity between the constructed sequence and the best-matched simulated sequence. For those simulated sequences, which are not matched by any of the constructed sequences, there will not be any base identity score. We observe that in general, the sequences constructed by QuasQ have a higher match to the simulated sequences than those by the other three methods (Additional file 1:Figures S2–S54).

**Figure 4:**
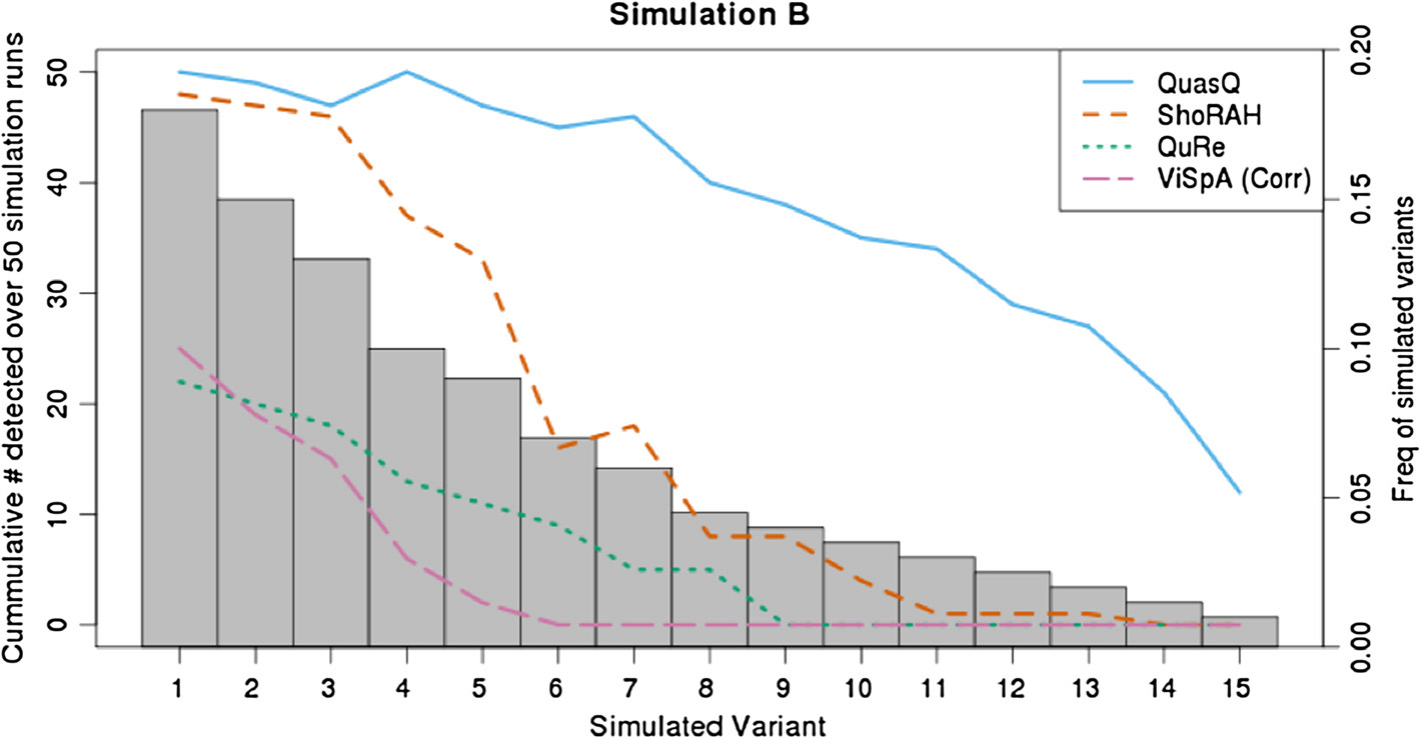
**Characterization of frequencies of haplotypes detected for Simulation B.** The detected simulated variants (out of a total of 15) for each of the 50 runs of simulations with type B setting (Table 2) were tabulated. This figure plots the cumulative count of each of the simulated variants constructed over the 50 runs of simulation B for QuasQ (blue), ShoRAH (red), QuRe (green) and ViSpA with ShoRAH corrected reads (pink) as described by the left vertical axis. The grey bars show the respective frequencies of the simulated variants as described by the right vertical axis.

In addition, we characterized the unique variants detected across the runs of each simulation type by its frequencies (Figure 4 and Additional file 1: Figure S55). The rationale behind this is that the simulated variants with the highest frequencies should be detected more easily than those of lower frequencies. QuasQ systematically outperforms ShoRAH, QuRe and ViSpA (corrected) especially for variants of frequencies < 0.2.

### Subtype B HIV-1 quasispecies results

We also compared the recall rates, precision and F-measure of the 4 softwares in reconstructing quasispecies variants of subtype B HIV-1, where the variant sequences have been established previously in an experiment by Zagordi et al. [16]. We observed that QuasQ has a higher recall rate as compared to QuRe, ViSpA and ViSpA ran with ShoRAH corrected reads but was lower than that by ShoRAH and returned the highest in precision of reconstruction of the variants of 1 among all 4 softwares. Overall, QuasQ achieved the highest F-measure (Table 5).

**Table 5:** **Summary of the recall rate, precision and F-measure for QuasQ, ShoRAH, QuRe, ViSpA, ViSpA (using ShoRAH corrected reads), QuasiRecomb (no recombination) and QuasiRecomb (with recombination) in reconstruction of the subtype B HIV-1 *pol *** **gene quasispecies**

	**Recall**	**Precision**	**F-measure**
QuasQ	0.700	1.000	0.824
ShoRAH	0.900	0.153	0.261
QuRe	0.600	0.154	0.245
Vispa	0.600	0.005	0.010
Vispa (Corrected)	0.400	0.250	0.308
QuasiRecomb (No recomb)	0.500	0.004	0.008
QuasiRecomb (With recomb)	0.500	0.001	0.002

**Figure 5:**
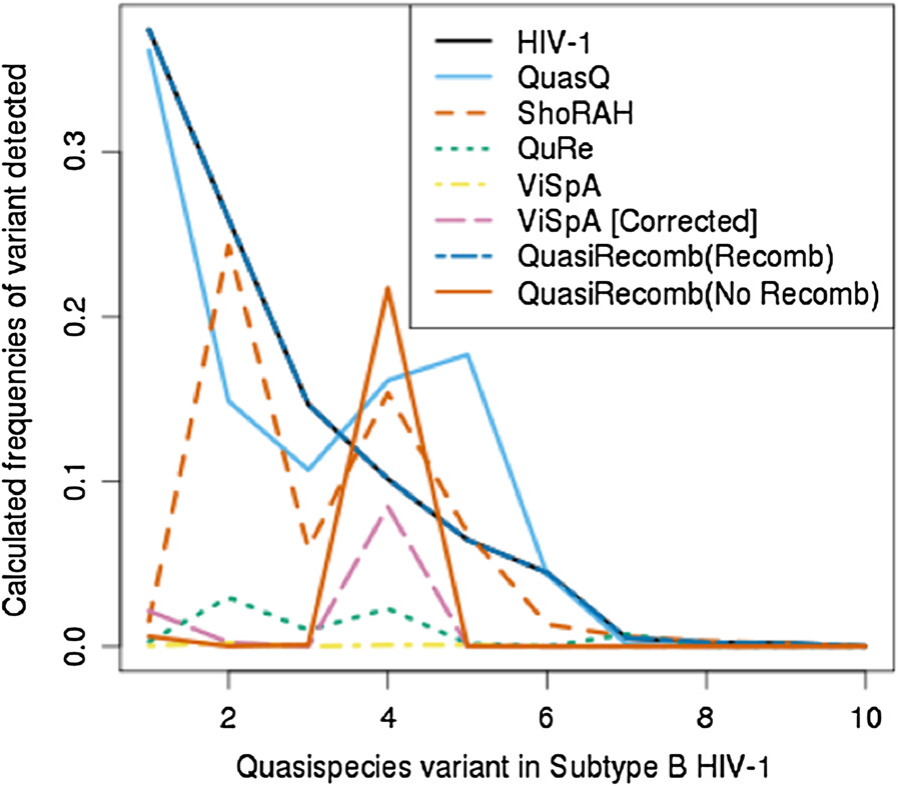
**Estimated frequencies of the haplotypes detected for HIV-1 dataset.** Frequencies of each of the best matched constructed haplotypes were estimated and plotted with the known frequencies of the sequenced variants. QuasQ (blue) and ShoRAH (red) constructed haplotypes are estimated with FreqEst while QuRe (green), ViSpA (yellow),ViSpA with ShoRAH corrected reads (pink), QuasiRecomb without recombination (turquoise) and Quasirecomb with recombination (orange) are estimated with their respective frequency estimators.

Figure 5 shows the estimated frequencies of the constructed haplotypes that are best matched to the subtype B HIV-1 variants. The trend of the frequencies of the haplotypes constructed by QuasQ resembles the original frequencies of the variants most.

**Figure 6:**
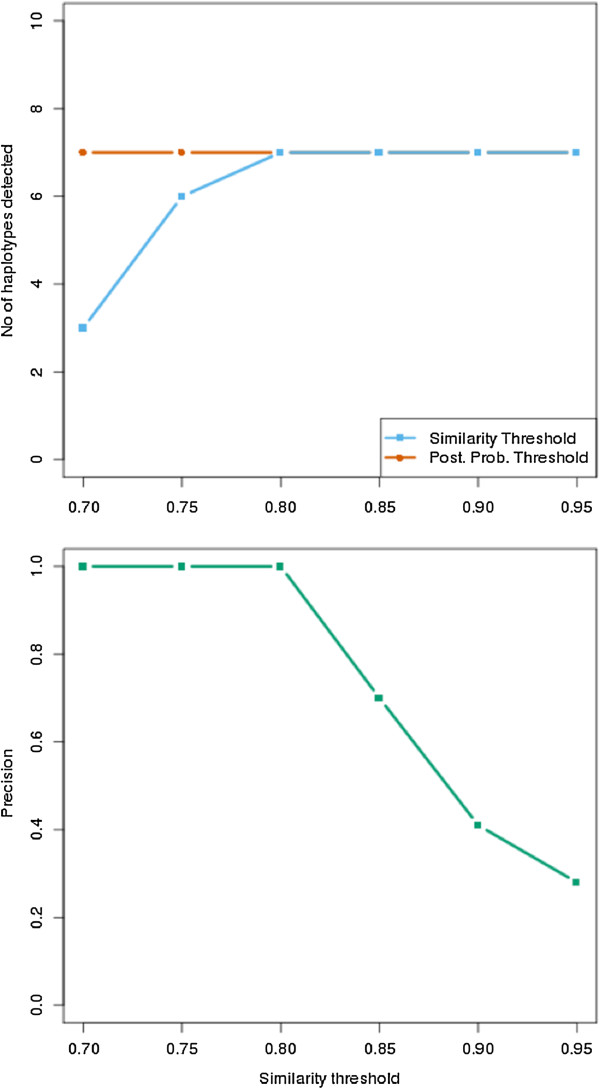
**Efficacy of QuasQ the similarity parameter during collapsing. (a)** Number of simulated variants of the subtype B HIV-1 *pol* gene quasispecies detected at different posterior probability threshold (0.7 to 0.95) with similarity threshold held constant at 0.9 (red) and different similarity threshold (0.7 to 0.95) with posterior probability held constant at 0 (blue). **(b)** Precision, i.e. the proportion of unique simulated variants detected with respect to the total number of constructed haplotypes, of QuasQ at detecting true variants at different similarity threshold.

In addition, we studied the efficacy of the collapsing algorithm by varying both the posterior probability and the similarity parameters (Figure 6). The total number of haplotypes constructed by QuasQ before collapsing was 70, of which 7 true variants were detected. It is observed that variation of the posterior probability (with similarity constant at 0.9) did not show significant difference in the number of detected true variants. Hence, we do not impose a posterior probability threshold. Posterior probability is calculated and used in determining the most probable branch during collapsing. However, as expected, when the similarity parameter is varied from 0.7 to 0.9 (with posterior probability threshold kept constant at 0), the number of detected true variants increased. This is expected because at low similarity threshold, pairs of haplotypes that are not too similar (eg 70% similarity) may be collapsed to one haplotype. The precision of QuasQ at varying thresholds, however, remained rather high.

### Clinical DENV data from Thailand

We apply QuasQ, ShoRAH, ViSpA, QuRe and QuasiRecomb to reconstruct the quasispecies sequences of the five dengue virus strains from the four serotypes, although we emphasize the results do not necessarily have any bearing on the relative performance of the approaches given the absence of any knowledge on the underlying quasispecies diversity.

The 454 pyrosequencing of the five isolates generated 54,440 reads in total, where 51,731 (95%) reads remain after filtering reads with at least 1 N call, and 50,633 (93%) reads remain after a second filter based on removing sequence reads with lengths in the 1 percentile of either extreme. Each sequence read is then mapped against the reference genomes of the four serotypes. The average read length and corresponding standard deviation of the sequence reads for serotypes 1, 2 and 3 are summarized in Table 6, suggesting that sequence output across the three isolates are comparable. As with the findings from the simulations, QuasQ inferred less number of haplotypes than ShoRAH and QuasiRecomb but more than ViSpA and QuRe, although we emphasize it is currently not possible to infer the relative performance of the methods from this observation. In addition, ViSpA (using ShoRAH corrected reads) inferred comparable number of haplotypes as with QuRe.

**Table 6:** Summary of the mean and standard deviation of the read lengths from 454 pyrosequencing of serotypes 1, 3 and 4 of the dengue virus obtained from lab cultures in Thailand

**Serotype**	**Mean**	**SD**	**QuasQ**	**ShoRAH**	**QuRe**	**ViSpA**	**ViSpA (Corrected)**	**QuasiRecomb (No recomb)**	**QuasiRecomb (With recomb)**
**1**	224.1	52.7	7	203	1	566	2	91	90
**3**	223.7	51.7	33	205	2	516	4	24	23
**4**	209.9	61.6	68	145	2	188	3	-	-

The last seven columns show the number of constructed haplotypes from QuasQ, ShoRAH, QuRe, ViSpA, ViSpA (with ShoRAH corrected reads), QuasiRecomb (no recombination) and QuasiRecomb (with recombination).

The haplotypes constructed by each of the four softwares are aligned back to the reference of the respective serotypes using BLASTn (*E value* <0.001). Additional file 1: Figure S56–S58 shows the top scoring alignment where the points represent the positions where the haplotypes differ from the reference sequence. Across all three serotypes, QuRe constructed haplotypes that aligned to <20% of the reference genome. Majority of the haplotypes constructed by ViSpA, before using ShoRAH corrected reads, aligned to ≤80% of the reference genome with ≤80% identity. And it is observed that ViSpA constructs haplotypes with significantly higher hamming distance to the reference genome as compared to ShoRAH and QuRe and this number is drastically decreased when ViSpA is used with ShoRAH corrected reads; the constructed haplotypes by ViSpA with ShoRAH corrected reads have significantly lower hamming distance as compared to the haplotypes constructed by ShoRAH and QuasQ.

In addition, we investigated the protein similarity between the constructed haplotypes and the reference genome using tblastx (*E value* <0.001) and found that, with the exception of ViSpA (before correction of reads), all the other softwares returned an identity in excess of 99% when aligned to protein sequence of the respective reference genome. Haplotypes constructed by ViSpA (before correction of reads) have identities of 94-98%. Haplotypes constructed by QuasQ show slightly higher protein similarity with the reference genome than those constructed by ViSpA (with ShoRAH corrected reads), followed by ShoRAH, QuRe and ViSpA (before correction of reads) as summarized in Table 7.

**Table 7:** Summary of the alignment result of the translated haplotypes of serotypes 1, 3 and 4 of the dengue virus obtained from lab cultures in Thailand constructed by QuasQ, ShoRAH, QuRe, ViSpA, ViSpA (with ShoRAH corrected reads), QuasiRecomb (no recombination) and QuasiRecomb (with recombination) with the translated reference genome

	**Serotype 1**	**Serotype 3**	**Serotype 4**
**QuasQ**	98.3%	60.1%	75.0%
**ShoRAH**	45.0%	37.3%	33.4%
**QuRe**	18.2%	23.6%	13.5%
**ViSpA**	3.0%	3.1%	3.1%
**ViSpA (Corr)**	96.3%	40.5%	71.0%
**QuasiRecomb (No recomb)**	98.3%	60.3%	-
**QuasiRecomb (With recomb)**	98.3%	60.3%	-

The table gives the average percentage of the respective reference genome length with which the constructed haplotypes are aligned to.

## Discussion

We have introduced a strategy QuasQ that utilizes the base quality scores of each sequence read to infer the authenticity of a mutation in reconstructing the genome sequences of viral quasispecies that are present within a single human host. Owing to the fact that each host carries multiple variants from a single serotype, inferring the number of quasispecies that are actually present as well as reconstructing the genome sequences of these different quasispecies is not trivial. While there are available softwares for performing this sequence alignment, these typically do not incorporate the quality of the sequence base calling, which can be valuable in deciding whether an observed polymorphic site contains a genuine mutation or is attributed to a base calling error. In an ideal setting, the presence of a single discordant site between two genome sequences should indicate that these genome sequences belong to two closely-related yet distinct variants, however in practice these two sequences are likely to be identical and the single discordant site is very likely a base calling error. With the novel collapsing method introduced in this paper, we have showed effectiveness in the reduction of such haplotypes caused by sequencing errors. Our simulations and comparisons on the real HIV-1 data have indicated that QuasQ outperformed the existing methods with publicly available software, by: (1) identifying higher number of true variants, while (2) minimizing the number of false variants, (3) where the reconstructed haplotype sequences exhibit higher degree of genetic similarities with the true sequences and (4) reconstructs haplotypes with frequencies resembling that of the original data. These findings strongly indicate that a sequence alignment strategy for viruses that utilizes the base quality scores ends up inferring a lower number of variants in a quasispecies, as this will down-weigh or even remove the contribution of polymorphic sites that are mainly attributed to base calling errors.

The ability to correctly infer the individual sequences of the quasispecies opens up vast opportunities in investigating the epidemiology of viruses, such as dengue. Consider a setting where dengue patients are admitted to a clinic and blood samples are obtained daily, allowing a longitudinal tracking of the evolution of dengue virus diversity. Association analysis can subsequently be performed to correlate clinical dengue symptoms with quasispecies diversity, allowing the functional impact and virulence of either individual or clusters of mutations to be assessed. In such a longitudinal setting, the ability to track dominant variant of a quasispecies will also allow researchers to identify specific strains that are more adaptive and resistant to innate host immunity, which allows the assessment of the mutations that correlate with this resistance. Understanding the genomic architecture of antibody resistance in the dengue virus is expected to be vital in designing effective vaccines. In addition, identifying the responsible mutations for dengue severity may provide the potential for predicting the outcome of dengue infection, whether it is the milder dengue fever or the more severe hemorrhagic form. The latter application has important public health implication, as dengue is more prevalent in developing countries and prolonged monitoring of dengue patients in a hospital setting for the purpose of minimizing hemorrhagic fever can place a considerable burden on the healthcare system.

## Conclusions

By introducing the ability to infer the viral quasispecies colony that is present within a human host, QuasQ provides the potential for a more accurate classification of the viral phenotype. Perhaps the future of viral genomics will focus on comparing healthy controls who are carriers of virulent strains of the quasispecies against subjects exhibiting severe clinical symptoms of the infectious disease but are in fact affected by less virulent strains of the quasispecies. It is clear that understanding the genomics of viruses will be relevant not just to studying how to control or even eradicate these viral infectious diseases, but also in learning about the innate protection in the human host against the viruses.

## Methods

Our algorithm QuasQ assumes the availability of high-throughput DNA data sequenced using 454 pyrosequencing. This technology produces reads with average read lengths of 330 bases, and yields per-base quality scores calibrated on the Phred scale for all valid calls [17]. This base quality score maps directly to the probability that a particular base is incorrectly called due to a sequencing error. The distribution of base quality scores from a clinical application of 454 sequencing of DENV (the details of the sequencing can be found in subsection “Dengue virus template preparation and sequencing”) is shown in Additional file 1: Figure S1. When the sequencing is unable to generate a valid call of one of the four bases on a particular read, a null call or “N” is assigned. By incorporating this information on base quality and read quality, the QuasQ algorithm thus contains four components: (1) pre-processing and quality checking of the raw sequence reads, and subsequently mapping the high quality reads to the reference genome; (2) local correction of sequencing errors; (3) global reconstruction and collapsing of haplotypes; and finally (4) inferring the number of variants and their respective likelihoods and frequencies.

### Pre-processing

A small fraction of the reads tend to possess more sequencing errors than others in 454 sequencing. These low quality reads thus contribute disproportionately to the total error rates. It has been reported previously that sequence reads (i) containing one or more N calls; or (ii) with lengths that are either extremely short or long are often indicative of low-quality sequencing [18]. We thus remove any reads that contain at least one N call, or are of extreme lengths (defined as sequence reads with lengths in the 1 percentile of either extremes of the distribution, see Additional file 1: Figure S2 for the distribution of average base quality scores against read lengths).

### Mapping to the reference genome

The reads that remain from the pre-processing are mapped against the reference genome using Bowtie 2.0.0 [19] (see Additional file 1). The sequencing process can occasionally introduce in vivo artifacts and produce high-quality sequence reads that are not reflective of the target genome. If undetected, these artifacts will erroneously increase the diversity of the haplotypes constructed. We thus implement an additional step of mapping each read against the reference genome while permitting some discordance to reflect genuine rapid evolutionary changes of variants as compared to the reference genome. Our pipeline retains only those reads that were uniquely mapped and where at least 80% of each read maps to the reference genome with at least 80% similarity.

A known source of problem with 454 pyrosequencing is the tendency to incorrectly ascertain the sequence content in the presence of a homopolymer (a stretch of identical bases), resulting in either an undercount or overcount of the identical bases [18]. To accommodate the possibility of such homopolymer-induced errors, our algorithm allows indels to be inserted or deleted with respect to the reference genome.

### Local error correction

A mutation in the haplotypes may either be genuine and explains the diversity of the variants within a quasispecies, or it can be an error introduced during sequencing. To minimize the occurrence of the latter, we implemented a sliding window clustering algorithm to correct for sequencing errors using the co-variation of the alleles. At every window, all combinations of alleles that have frequencies ≤ 0.5% of the coverage at that window are identified and clustered with those of the shortest hamming distance. The intuition behind this is that combinations of alleles that occur singly are more likely to be caused by sequencing errors, which are rare and occur randomly, as compared to true mutations, which should occur in proportions relative to their respective frequencies in the population.

### Quasispecies sequence reconstruction

**Figure 7:**
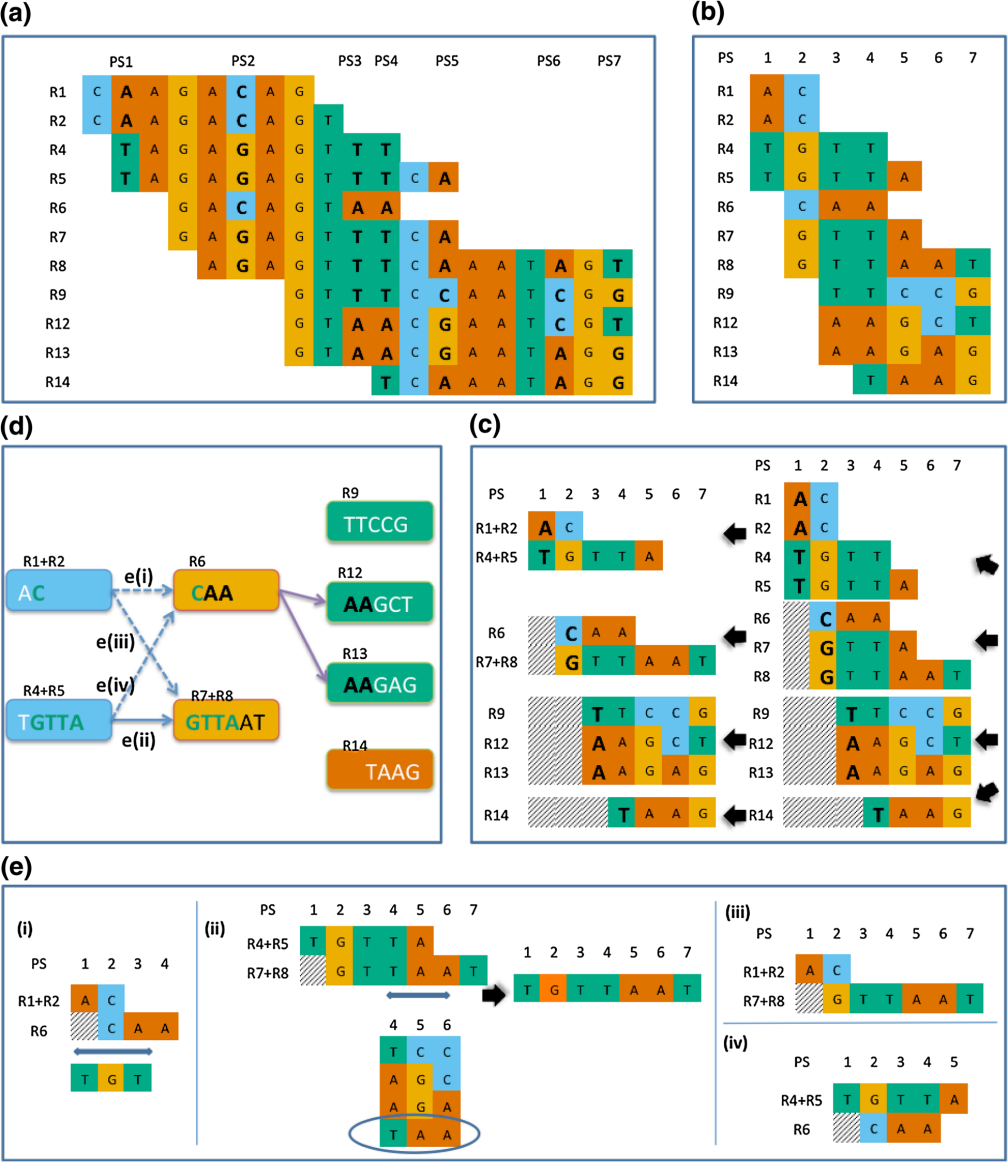
**QuasQ quasispecies sequence reconstruction.** Post-processed local error-corrected reads are stacked, to identify **(a)** all polymorphic sites (PS) having two or more alleles. **(b)** The genomic sequence is then ‘reduced’ to consist of only the polymorphic sites identified in **(a)**. All mapped reads are similarly ‘reduced’ to their sequenced bases in PS. **(c)** These ‘reduced’ reads are then grouped into disjoint sets according to their starting PS positions, and in each set, the longest representative reads based on sequence identity would be identified (R4 collapsing into R5). **(d)** A read-graph method is then applied to connect the disjoint sets of representative merged reads, where each node consist of the read sequence of DNA bases, with directed edges to connect two nodes if the first node is a complex prefix of the second node. Solid arrows such as **e**(ii) represent possible directed edges, while non-probable edges such as **e**(iii) and **e**(iv) due to non-identical node sequences overlap are represented by dotted arrows. For **e**(i), though identical overlap occurs between nodes R1 + R2 and R6, it is not considered probable by QuasQ as there is no sequence read spanning the nodes and the immediate neighboring polymorphic site. A region that is centered at the right-most polymorphic site of the overlap, with coverage greater than 10^th^ percentile of the total coverage of the reads is defined. The coverage of the region is important since if the coverage is low, the absence of any supporting read is not necessarily an indication that the merging of the two contiguous segments is false. For pairs of reads with identical overlap **e**(i), if the allele combination in the defined region is not the same as that of any other read in that region, the two nodes are not joined. In this figure, the only constructed haplotype is thus *R4 + R5 + R7 + R8*.

The quasispecies genome sequences are reconstructed with the corrected reads. This reconstruction happens within each serotype. A pictorial illustration of the reconstruction is shown in Figure 7.

The reads after post-processed for local error correction are piled-up by positions, and all polymorphic sites (PS) where two or more alleles are present will be identified (Figure 7a). The reference genome is then ‘reduced’ to consist of only the polymorphic sites identified earlier (Figure 7b). Similarly, all mapped reads are ‘reduced’ to consist only of the bases called in the same polymorphic sites. The ‘reduced’ mapped reads are then grouped into disjoint sets according to their starting polymorphic sites (Figure 7c). The longest representative reads based on sequence base identities would therefore be identified for each set of reads, such that shorter reads that are complete subsequence of the longer reads will be filtered out. A read-graph method is then applied to connect the disjoint sets of representative merged reads in ascending order of the positions of polymorphic sites, where each node consist of the read sequence of DNA bases, with directed edges to connect two nodes if the first node is a complex prefix of the second node (Figure 7d). For any two reads that overlap, it is important to ensure that the overlapping region spans genuine polymorphic sites that are not the result of in vitro artifacts. To evaluate this, the method looks for at least one sequence read that not only spans the overlapping portion but also extends to include the polymorphic site immediately adjacent to the right and, if possible, left flank of the overlapping segment, depending on the coverage (Figure 7e).

**Figure 8:**
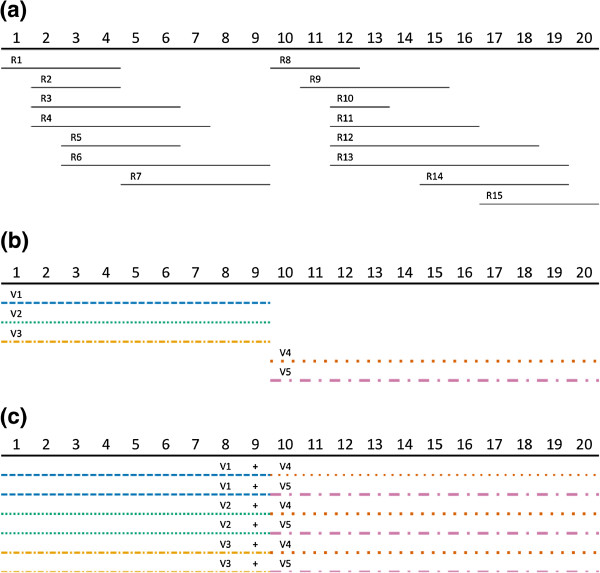
**Joining constructed sequences from disjoint blocks. (a)** Here, we have the situation, where we can separate all sequenced reads into two disjoint blocks, Block 1 (positions 1 to 9) and Block 2 (positions 10 to 20) as there are no reads that span across positions 9 and 10. We then construct the haplotypes in each block independently as in **(b)** to obtain 3 possible haplotypes (V1, V2, V3) in Block 1 and 2 possible haplotypes (V4, V5) in Block 2. We then explore the possibilities of combinations of all 6 (3x2) possible haplotypes between the two blocks in **(c)**.

In the situation when there are no reads that overlap between two consecutive polymorphic sites, the combined sequences from before and after the gap are constructed separately; these sequences are subsequently joined in all possible ways (Figure 8).

### Likelihood construction

We calculate the likelihood of each constructed haplotype by factoring into account the chance that a polymorphic site is attributed to a sequencing error. The intuition behind this inference is that a sequencing error is more likely to have happened when: (1) only a small proportion of the reads spanning a polymorphic site carry the minor allele(s); (2) the average base quality score for the minor allele is low. It is however important to acknowledge the possibility that situation (1) can happen if it is a recent mutation and is thus present only in a small proportion of the quasispecies.

The base quality score (Q) is reported on a Phred scale:

$$Q=-10\mathrm{lo}g_{10}p,$$

where *p* is the probability that the called base is a sequencing error.

Hence, for a particular read of length *N*,

$$\log\left(P\left(\mathrm{No}\;\mathrm{errors}\;\mathrm{in}\;\mathrm{read}\right)\right)=\sum -\log\left(1-p_{i}\right)=\sum -\log\left(1-10^{-Q_{i}/10}\right),$$

where the summation is performed across all the *N* bases.

For each constructed haplotype sequence, the reads spanning each polymorphic site can be divided into two categories: (a) reads with the exact same base composition as the constructed haplotype sequence; (b) reads without the exact same base composition as the constructed haplotype sequence. We are thus interested to calculate the likelihood for the observed data spanning each constructed haplotype sequence given that this haplotype sequence is real. This means that at each polymorphic site, either all the reads are correct (thus allowing the relative frequencies of the different alleles to be estimated from the proportion of the reads carrying each allele) or that those reads carrying a different allele at this site are erroneous.

P(Observed base | Sequence is real) =

P(Observed base | Sequence is real, all reads without errors) ×

P(All reads without errors) +

P(Observed base | Sequence is real, category (a) contains errors) ×

P(category (a) contains errors) +

P(Observed base | Sequence is real, category (b) contains errors) ×

P(category (b) contains errors).

The -log-likelihood for each constructed haplotype sequence is thus:

$$\sum -\log P\left(\mathrm{Observed}\;\mathrm{base}\;j\left|\;\mathrm{Sequence}\;\mathrm{is}\;\mathrm{real}\right)\right),$$

where the summation is performed across all bases.

The posterior probability for sequence *k* out of a pair of possible sequences (*k*, *l*) is thus calculated as

$$\frac{\mathrm{likelihood}\;\left(\mathrm{sequence}\;k\right)}{\mathrm{likelihood}\;\left(\mathrm{sequence}\;k\right)+\mathrm{likelihood}\;\left(\mathrm{sequence}\;l\right)}.$$

### Inferred haplotype construction

Using the aligned sequences for the inferred haplotype of a quasispecies, we then proceed to construct a neighbor-joining tree similar to the method by Saitou and Nei [20], where the distance between any two sequence alignments (inferred haplotypes) is simply quantified as the proportion of sites that differ. As every branch on the tree corresponds to an inferred haplotype sequence with a known likelihood score, two neighboring branches are collapsed into a single branch based on two parameters. The first parameter is the patristic distance between the two branches where both branches will be collapsed if the distance is less than a user-specified threshold. The second parameter is the difference in posterior probabilities of the two branches where both branches will be collapsed if the difference between them is larger than a user-specified threshold. The posterior probabilities for the two branches or sequence haplotypes *k, l* are calculated as described in the earlier paragraph. The intuition behind this branch collapsing is to remove highly similar haplotype sequences and retain only the sequence that is most probable.

The frequencies of the constructed haplotypes are subsequently inferred using *freqEst*[8], an Expectation-Maximization (EM) algorithm, which allows the most prevalent haplotype to be identified. The probability distribution of each of the constructed haplotypes (*p*_*1*_,…,*p*_*H*_), where H is the set of all the constructed haplotypes, is estimated by maximizing the log-likelihood function of the probability of observing a read drawn with uniform probability, across all the reads that are consistent with the constructed haplotypes.

### Simulation set-up

In order to compare the performance of QuasQ against ShoRAH, we performed a series of simulations on artificial DENV quasispecies data generated from DENV 1 strain Hawaii. Reads are simulated using ART 454 (Version 2.1.8)[21] (see Additional file 1) which mimics the real error profile of the reads generated by the 454 FLX machine. We varied the following three factors that affect the genome reconstruction of the DENV quasispecies after sequencing:

(1) *Number of real variants within quasispecies*.

One important element in reconstructing viral genomes is in inferring the number of variants within a quasispecies that is present within each human host, since this varies across different hosts and can directly impact the sequence reconstruction. In addition, as Eriksson et al. [8] and Prosperi et al. [12] have shown, the frequencies of the variants within a simulated dataset affects the confidence in reconstruction. We consider two scenarios in our simulations where there are actually 10 and 15 different genome sequences, with frequency profiles following geometric distributions. All the reads are randomly generated from these sequences using ART 454 producing reads of average read length of 350 bp.

(2) *Mutation rates*.

Here, we investigate the robustness of QuasQ to different mutation rates between different haplotypes of a quasispecies. We use a per-base mutation rate 0.02 or 0.05 per generation as observed in [22], such that the number of mutations in the offspring copy from the parent copy follows a Poisson distribution with a mean of 200 or 500.

(3) *Overall coverage of the reads*.

Deep sequencing of genomes promises the information for detection of low frequency mutations but brings with it higher chances of sequencing errors in the increasing number of reads. The ability to identify low-frequency variants at low coverage and also to be able to differentiate sequencing errors from true polymorphisms in data at high coverage is what we want to test for. Hence, we simulate the datasets at ~700x and ~1500x coverage with average of ~30,000 and ~60,000 reads respectively.

Our simulations assume a total genome size of 10,700 basepairs, and the ancestral sequence is taken to be DENV 1 strain hawaii. We adopt a sequential approach in generating the quasispecies genome sequences, where the *i*^th^ variant is effectively a copy of one of the previous (*i* – 1) variants except at the mutation sites. The resultant genome sequences thus constitute the true data that we will attempt to reconstruct with QuasQ, ShoRAH, ViSpA, QuRe and QuasiRecomb. Aside from varying the total number of sequences generated, we vary, too, the frequencies of each of these sequences and finally used ART 454 to generate reads of different coverage. There are eight simulation scenarios we consider after accounting for the different combinations of the three factors (see Table 2), and 50 datasets are simulated within each scenario.

### Subtype B HIV-1 quasispecies

We attempt to reconstruct the viral quasispecies from a real experiment in which the sequence of the variants of the quasispecies sequenced was known beforehand, and thus can be used to access the effectiveness of each software. The dataset from the experiment by Zagordi et al. [16], in which 10 different clinical isolates of subtype B HIV-1 quasispecies were pooled in a mixed sample in different proportions (at a maximum contribution of 30% from each isolate, at an average diversity of about 7%, and with an estimated rate of heterogeneity of 0.35). The first 1,245 bases of the *pol* gene were sequenced without PCR amplification using 454 FLX Titanium resulting in 16,540 reads.

### Dengue virus template preparation and sequencing

In the application of QuasQ to actual dengue viruses, we isolated five DENV prototype strains commonly used in the laboratory consisting of: (i) serotype 1, strain Hawaii (acc: EU848545); (ii) serotype 2, strain NGC (acc: M29095); (iii) serotype 2, strain 16681 (acc: U87411); (iv) serotype 3, strain H87 (acc: M93130); and (v) serotype 4, strain H241 (acc: AY947539). These prototypes are cultured in C6/36 cell line obtained from the Dengue Hemorrhagic Fever Research Unit, Siriraj Hospital in Bangkok, Thailand. Dengue RNA was extracted from the viral-culture supernatant with the QIAamp Viral RNA Kit (QIAGEN) and converted to cDNA by SuperScript III First-Strand Synthesis System (Invitrogen). The AccuPrime™ Taq DNA Polymerase High Fidelity (Invitrogen) was used to amplify the cDNA template. The PCR product was purified using the QIAquick Gel Extraction Kit (QIAGEN) and the DNA concentration was measured using NanoDrop. Pooled dsDNA template of all four serotypes of the DENV was finally sequenced by the Genome Sequencer FLX System (Roche Company) according to standard protocol which starts from random fragmentation by Nebulizer.

### ShoRAH settings

The program settings for ShoRAH version 0.6 that were used in our analyses considered a window size of 330 basepairs, based on our average read lengths of 350 bp, and a recommended alpha of 0.1 for the Dirichlet process mixture. We also provided ShoRAH with a file that contains the appropriate serotype reference sequence. An example command line that was used is: python shorah.py –b INPUT.bam –r INPUT_REF.fas –w 330 –a 0.1 –k > global.log

### ViSpA settings

The program settings for ViSpA version 01 that were used in our analyses allow for (i) 1 (for corrected reads) or 5 (for uncorrected reads) mismatches when deciding whether a read is a sub-read of the super-read; and (ii) 105 (for simulations with mutation rate of 0.02) or 262 (for simulations with mutation rate of 0.05) mutations, in distinguishing between variants. We also provided ViSpA with a file that contains the appropriate serotype reference sequence. An example command line that was used is: sh main.bash INPUT.fas INPUT_REF.fas 12 (i) (ii).

We find that majority of the haplotypes constructed by ViSpA aligns to <80% of the length of any simulated variants, as described in the “Simulation Results” section later. Hence we excluded ViSpA in the later comparions. However, since ViSpA was packaged with the aligner, SEGEMEHL, as opposed to Bowtie that was ran for the other three softwards, to make it a fair comparison, Bowtie-aligned data was used during ViSpA’s analysis as well. Additional rounds of analysis were ran on ViSpA using ShoRAH corrected reads to test the viability of ViSpA’s alignment algorithm, despite the absence of the pre-processing step – ViSpA (Corrected).

### QuRe settings

The program settings for QuRe version 0.9997 that were used in our analyses considered the defaults of 0.0044 homopolymeric error rate, 0.0007 non-homopolymeric error rate and 2,500 iterations. A relatively small number of iterations was used because at a higher number, QuRe very often run out of memory. We also provided QuRe with a file that contains the appropriate serotype reference sequence. An example command line that was used is: java -Xmx4G QuRe INPUT.fas INPUT_REF.fas 0.0044 0.0007 2500

### QuasiRecomb settings

The program settings for QuasiRecomb version 1.1 that were used in our analyses considered both with and without recombination, without gaps and applied the conservative method. The example command lines there were used are:

Without Recombination: java -jar ~/src/QuasiRecomb.jar -i INPUT.bam -noGaps -quality -unpaired -conservative –noRecomb

With Recombination: java -jar ~/src/QuasiRecomb.jar -i INPUT.bam –noGaps -quality -unpaired -conservative

In assessing these five softwares, QuasQ, ShoRAH, ViSpA, QuRe and QuasiRecomb, we quantify and compare their performances in terms of (i) how many of the simulated variants have been correctly inferred by the software (recall rate); (ii) out of the total number of constructed haplotypes, how many unique simulated sequences are reported by the software (precision); and (iii) the degree of genetic similarity between the reconstructed haplotype sequences and the simulated variants.

## Competing interests

The authors declare they have no competing interests.

## Authors’ contributions

WTP, EX, YYT and RTO designed the study. KC, PM and PS generated the data. WTP, EX, LPW and AYC performed the analysis. WTP, EX, YYT and RTO wrote the manuscript. All authors read and approved the final manuscript.

## Supplementary Material

Additional file 1: Supplementary Data. Click here for file
