# Supplementary material for: Viral quasispecies inference from 454 pyrosequencing
Source: BMC Bioinformatics. 2013 Dec 5;14:355. doi: 10.1186/1471-2105-14-355 (PMC4234478; doi:10.1186/1471-2105-14-355)
Supplement: Additional file 1: — Supplementary Data. [file 1471-2105-14-355-S1.pdf]

SUPPLEMENTARY MATERIAL

Viral quasispecies inference from 454 pyrosequencing

Poh and Xia et al.

CONTENTS

**1**	**Supplementary methods and analyses**		**3**
	1.1	Example command to run Bowtie2	3
	1.2	Example command for tree construction with *phangorn*	3
	1.3	Example scoring of constructed haplotypes using BLASTn for simulated data	3
	1.4	Software settings used during analysis of Subtype B HIV-1 quasispecies	4
	1.5	Software settings used during analysis of Clinical DENV data from Thailand	4
**2**	**Supplementary figures**		**5**
	-	Supplementary Figure 1	5
	-	Supplementary Figure 2	6
	-	Supplementary Figure 3	7
	-	Supplementary Figure 4	8
	-	Supplementary Figure 5	9
	-	Supplementary Figure 6	10
	-	Supplementary Figure 7	11
	-	Supplementary Figure 8	12
	-	Supplementary Figure 9	13
	-	Supplementary Figure 10	14
	-	Supplementary Figure 11	15
	-	Supplementary Figure 12	16
	-	Supplementary Figure 13	17
	-	Supplementary Figure 14	18
	-	Supplementary Figure 15	19
	-	Supplementary Figure 16	20
	-	Supplementary Figure 17	21
	-	Supplementary Figure 18	22
	-	Supplementary Figure 19	23
	-	Supplementary Figure 20	24
	-	Supplementary Figure 21	25

- Supplementary Figure 22 26
- Supplementary Figure 23 27
- Supplementary Figure 24 28
- Supplementary Figure 25 29
- Supplementary Figure 26 30
- Supplementary Figure 27 31
- Supplementary Figure 28 32
- Supplementary Figure 29 33
- Supplementary Figure 30 34
- Supplementary Figure 31 35
- Supplementary Figure 32 36
- Supplementary Figure 33 37
- Supplementary Figure 34 38
- Supplementary Figure 35 39
- Supplementary Figure 36 40
- Supplementary Figure 37 41
- Supplementary Figure 38 42
- Supplementary Figure 39 43
- Supplementary Figure 40 44
- Supplementary Figure 41 45
- Supplementary Figure 42 46
- Supplementary Figure 43 47
- Supplementary Figure 44 48
- Supplementary Figure 45 49
- Supplementary Figure 46 50
- Supplementary Figure 47 51
- Supplementary Figure 48 52
- Supplementary Figure 49 53
- Supplementary Figure 50 54
- Supplementary Figure 51 55
- Supplementary Figure 52 56
- Supplementary Figure 53 57
- Supplementary Figure 54 58
- Supplementary Figure 55 59
- Supplementary Figure 56 60
- Supplementary Figure 57 63
- Supplementary Figure 58 67

3 Supplementary tables 68
- Supplementary Table 1 68

1 Supplementary Methods and Analyses

1.1 Example command to run Bowtie 2.0.0 (Beta 5)

The following is an example of the command line input used to perform the Bowtie2 analysis for mapping the sequence reads against the reference genome.

bowtie2 --sam-nohead --local -x *<ref>* -U *<sequence.fastq>* > *<output.sam>* 2> *<output.log>*

1.2 Example command for tree construction with *phangorn*

The following is an example of the R commands used to perform the tree construction with *phangorn*, for constructing the maximum parsimony tree using the inferred sequence alignment of the DENV quasi-species.

```
> library(phangorn)
> data <- read.phyDat("reads.fas", format="fasta", type="DNA")
> dna2 <- as.phyDat(data)
> tre.ini <- nj(dist.dna(as.DNAbin(data), model = "raw"))
> tre.pars <- optim.parsimony(tre.ini, dna2)
```

1.3 Example scoring of constructed haplotypes using BLASTn for simulated datasets

The following is an example of the scoring algorithm used for each constructed haplotype against the respective true simulated haplotypes for the simulation datasets.

i. Alignment of constructed haplotype to simulated haplotype using BLASTn:
   > blastn -query *<constructed haplotype>* -db *<simulated haplotypes (blastn built database)>* -task blastn -dust no -outfmt 7 -evalue 3> *<blastn output>*

ii. Consolidation of BLASTn results:
   a. Search for alignments that map to ≥80% of the reference genome length

```
# BLASTN 2.2.26+
# Query: HAP0_0.151615
# Database: ../db/simA_2_db
# Fields: query id, subject id, % identity, alignment length, mismatches, gap opens, q. start, q. end, s. start, s. end, evalue, bit score
# 26 hits found
HAP0_0.151615   simA_2_ref_0   99.54    10670   49    0   1       10670   1       10670   0.0     1.902e+04
HAP0_0.151615   simA_2_ref_0   80.70    57      10    1   10569   10624   10484   10540   1e-06   50.0
HAP0_0.151615   simA_2_ref_0   80.70    57      10    1   10484   10540   10569   10624   1e-06   50.0
HAP0_0.151615   simA_2_ref_0   100.00   16      0     0   722     737     737     722     0.96    30.1
HAP0_0.151615   simA_2_ref_1   98.48    10668   162   0   1       10668   1       10668   0.0     1.851e+04
HAP0_0.151615   simA_2_ref_1   80.70    57      10    1   10569   10624   10484   10540   1e-06   50.0
HAP0_0.151615   simA_2_ref_1   95.00    20      1     0   8719    8738    8738    8719    0.28    31.9
HAP0_0.151615   simA_2_ref_1   100.00   16      0     0   722     737     737     722     0.96    30.1
HAP0_0.151615   simA_2_ref_2   97.15    10668   304   0   1       10668   1       10668   0.0     1.787e+04
HAP0_0.151615   simA_2_ref_2   82.98    47      7     1   10569   10614   10484   10530   1e-05   46.4
HAP0_0.151615   simA_2_ref_2   100.00   16      0     0   722     737     737     722     0.96    30.1
HAP0_0.151615   simA_2_ref_8   95.88    10668   440   0   1       10668   1       10668   0.0     1.726e+04
```

   b. In this case, we would report that the constructed variant "HAP0_0.151615" aligns best to the simulated sequence "simA_2_ref_0" with identity score of 99.54%, i.e. the constructed variant requires 0.46% mutations to transform to the simulated sequence

1.4 Software settings used during analysis of Subtype B HIV-1 quasispecies

The following are the settings used for ShoRAH, QuRe, ViSpA and ViSpA using ShoRAH corrected reads during analysis of Subtype B HIV-1 quasispecies.

i. ShoRAH: We used a window size of 300 which is slightly smaller than the average read length of 315 and alpha 0.1

```
python shorah.py –b INPUT.bam –r INPUT_REF.fas –w 330 –a 0.1 –k > global.log
```

ii. QuRe: We used the defaults of 0.0044 homopolymeric error rate, 0.0007 non-homopolymeric error rate and recommended 10,00 iterations

```
java -Xmx4G QuRe INPUT.fas INPUT_REF.fas 0.0044 0.0007 10000
```

iii. ViSpA and ViSpA using ShoRAH corrected reads: We used Bowtie-aligned data and (i) 1 (for corrected reads) or 5 (for uncorrected reads) mismatches when deciding whether a read is a sub-read of the super-read and (ii) 367 mutations allowed to distinguish variants, assuming a mutation rate of 0.07 for this dataset [16]

```
sh main.bash INPUT.fas INPUT_REF.fas 12 (i) (ii)
```

1.5 Software settings used during analysis of Clinical DENV data from Thailand

The following are the settings used for ShoRAH, QuRe, ViSpA and ViSpA using ShoRAH corrected reads during analysis of Clinical DENV data from Thailand.

i. ShoRAH: We used a window size of 210 for all three serotypes, which is slightly smaller than the average read length of 213, 222 and 212 for serotype 1, 3 and 4 resepctively, and an alpha of 0.1

```
python shorah.py –b INPUT.bam –r INPUT_REF.fas –w 300 –a 0.1 –k > global.log
```

ii. QuRe: We used the defaults of 0.0044 homopolymeric error rate, 0.0007 non-homopolymeric error rate and recommended 10,00 iterations

```
java -Xmx4G QuRe INPUT.fas INPUT_REF.fas 0.0044 0.0007 10000
```

iii. ViSpA and ViSpA using ShoRAH corrected reads: We used Bowtie-aligned data and (i) 1 (for corrected reads) or 5 (for uncorrected reads) mismatches when deciding whether a read is a sub-read of the super-read and (ii) 105 (for serotypes 1 and 3 assuming a mutation rate of 0.02 [22]) and 262 (for serotype 4 assuming a mutation rate of 0.05 [22]) mutations allowed to distinguish variants

```
sh main.bash INPUT.fas INPUT_REF.fas 12 (i) (ii)
```

2 Supplementary Figure

Supplementary Figure 1.

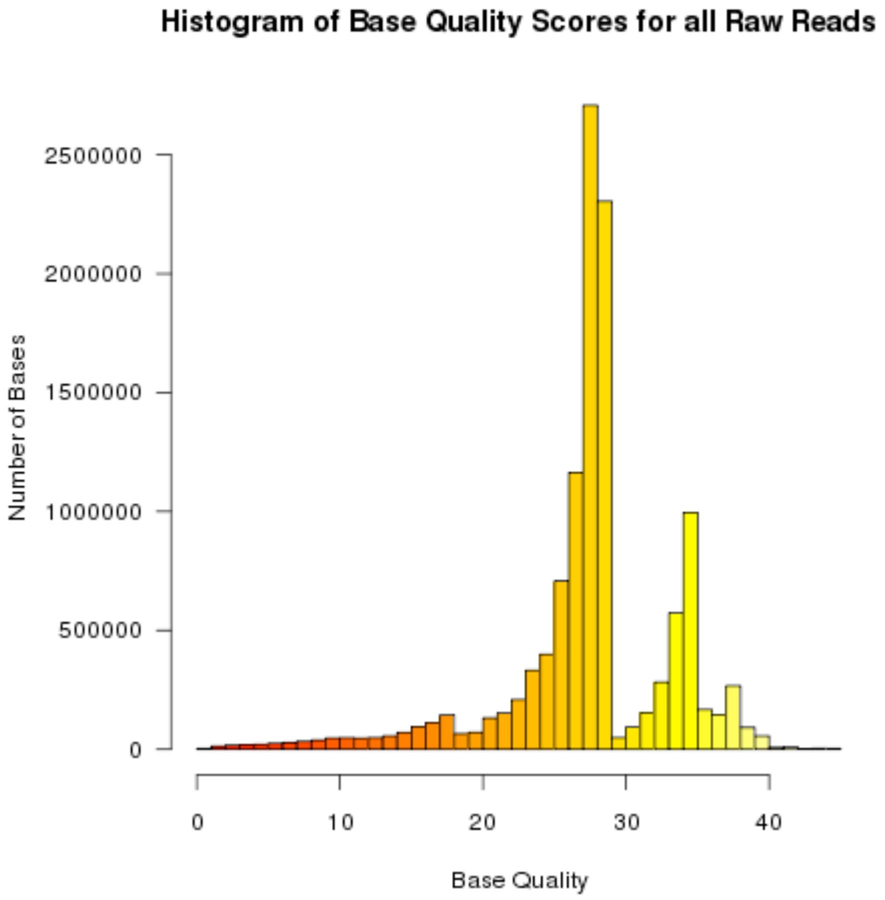

Supplementary Figure 1. Distribution of the quality for each base call in all the sequence reads from the 454 pyrosequencing of five prototype DENV strains from serotype 1 (strain Hawaii), serotype 2 (strain NGC), serotype (strain 16681), serotype 3 (strain H87) and serotype 4 (strain H241) from the Medical Molecular Biology Unit, Sririraj Hospital in Bangkok, Thailand.

Supplementary Figure 2.

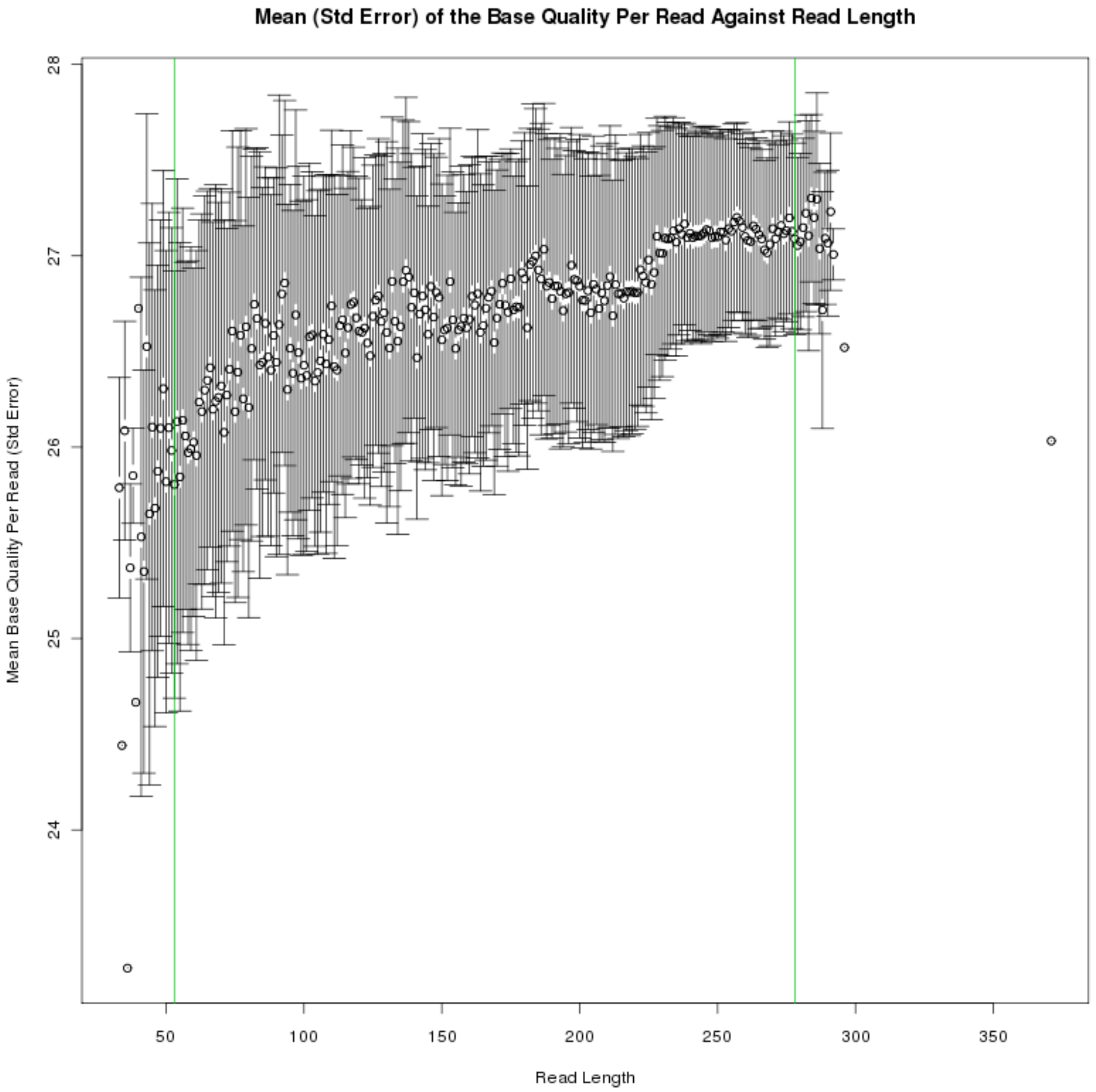

Supplementary Figure 2. Distribution of the average base quality scores against the sequence read lengths. There are 54,440 reads in total. We calculated the average of the base quality scores for each read, and define this as the read quality score. For each integer value of read length, we calculate the mean read quality scores (circle) and the corresponding standard error (vertical bars). The two vertical green lines correspond to the extreme 0.5^{th} percentile of the read length distribution in each direction.

Supplementary Figure 3.

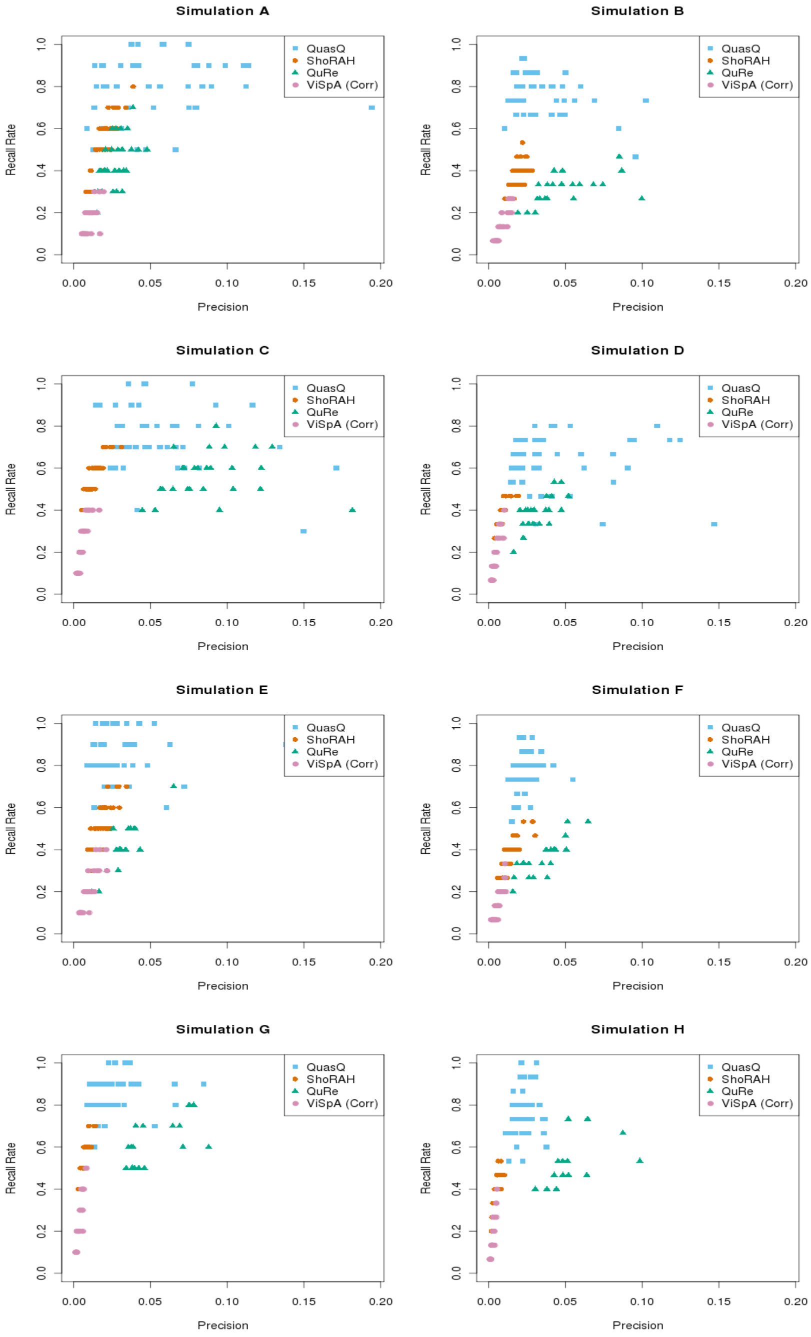

Supplementary Figure 3. Precision i.e. the proportion of unique simulated variants detected with respect to the total number of constructed haplotypes, vs recall rate, i.e. the numer of unique simulated variants detected out of the 10 or 15 simulated variants, for QuasQ (blue squares), ShoRAH (red diamonds), QuRe (green triangles) and ViSpA with ShoRAH corrected reads (pink circle) in reconstructing the quasispecies over 50 runs across each of the eight settings (A-H) respectively as described in **Table 1**.

Supplementary Figure 4.

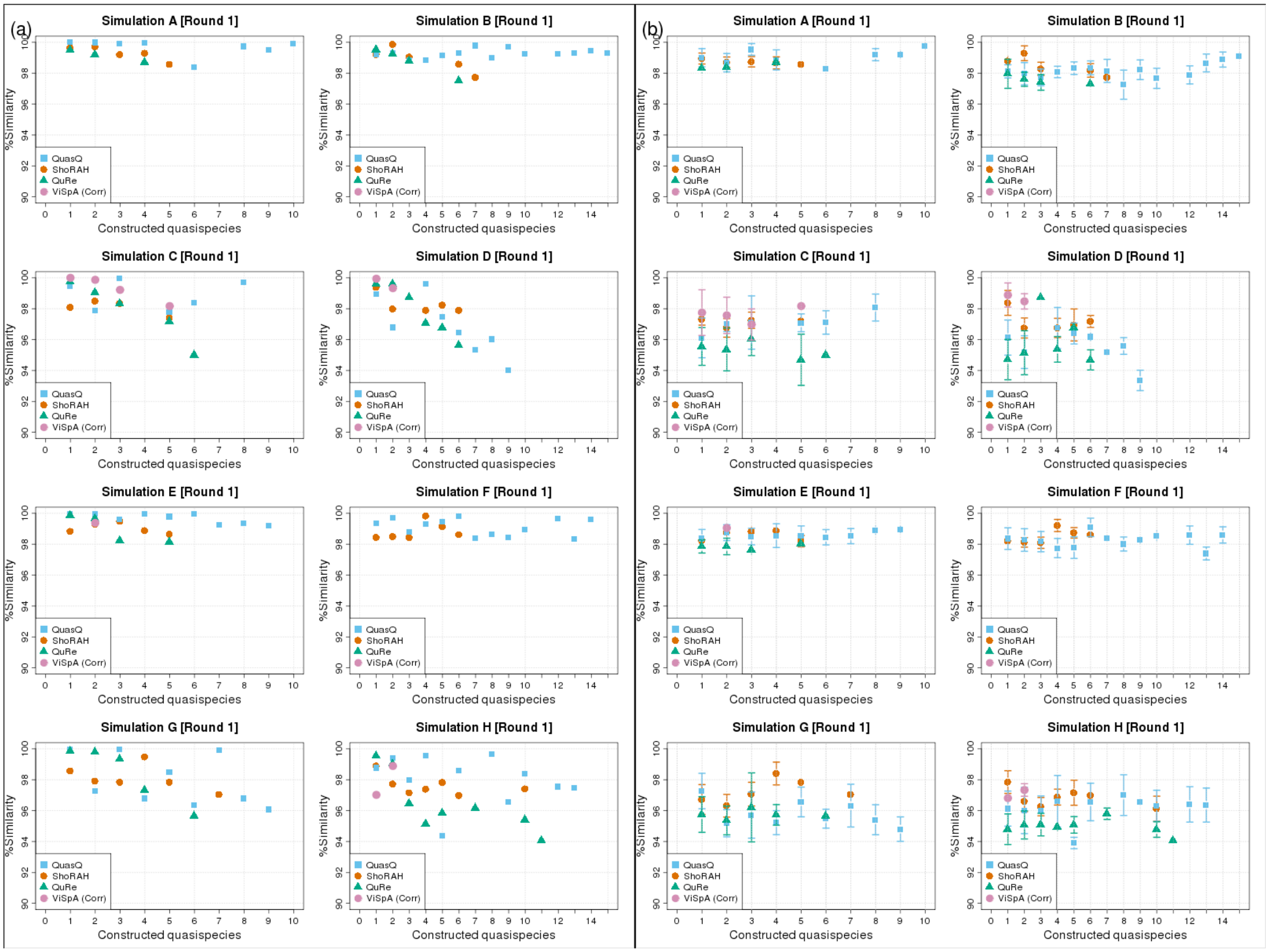

Supplementary Figure 4. (a) Degree of similarity between the best reconstructed haplotype sequences from QuasQ (blue squares), ShoRAH (red diamonds), QuRe (green triangles) and ViSpA with ShoRAH corrected reads (pink circle) with the actual simulated sequences. Here we identified the most similar reconstructed sequence to the simulated variants. (b) This shows the mean and standard deviation of the similarity for the reconstructed sequences that are mapped to a particular simulated variant. The above figures are based on the data from the first round of the simulations, and correspond to the genealogy trees of the simulations that are shown in **Supplementary Figure 5**.

Supplementary Figure 5.

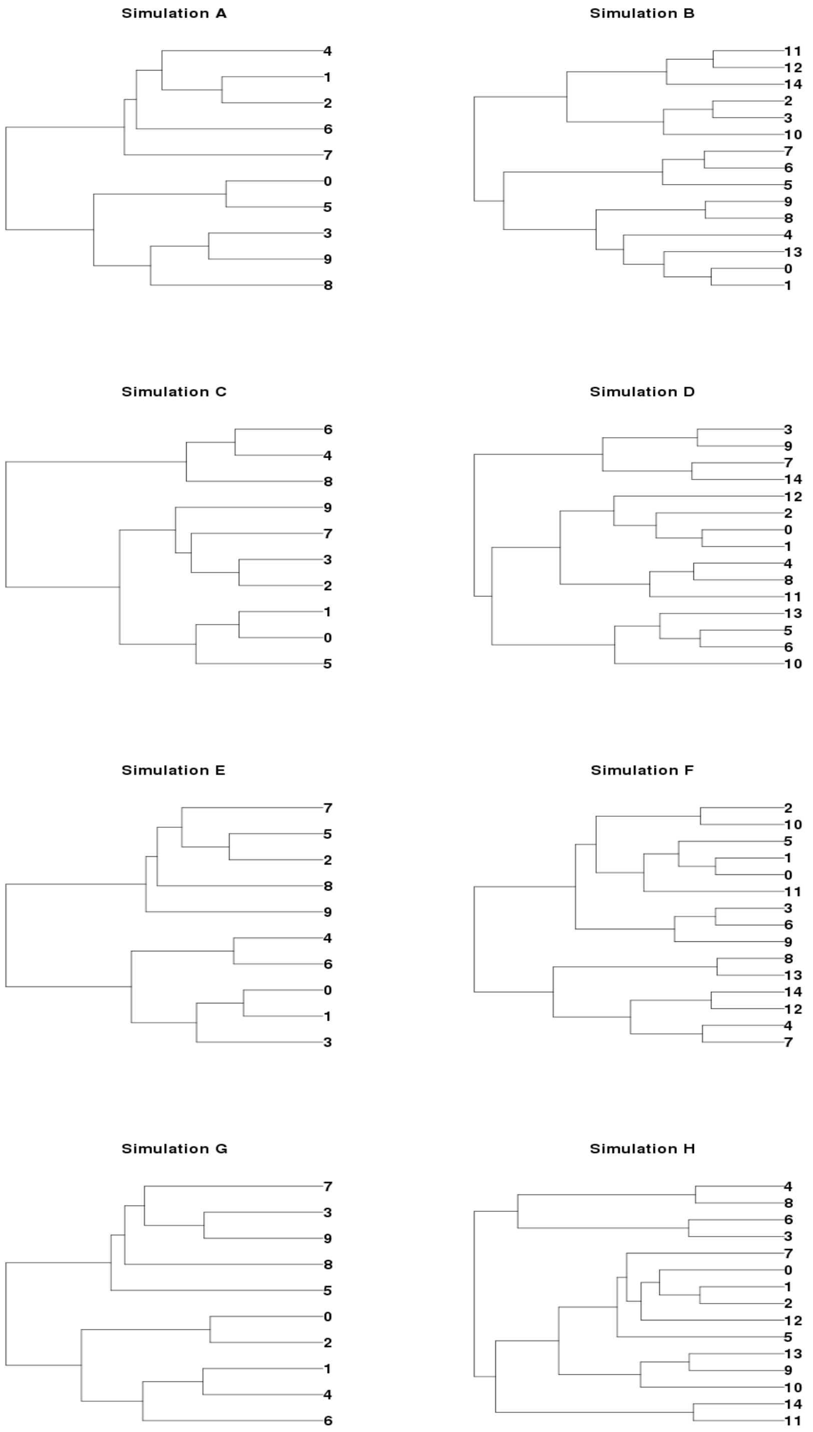

Supplementary Figure 5. The eight genealogy trees illustrating the relationship between the simulated quasispecies in the first round of the simulations.

Supplementary Figure 6.

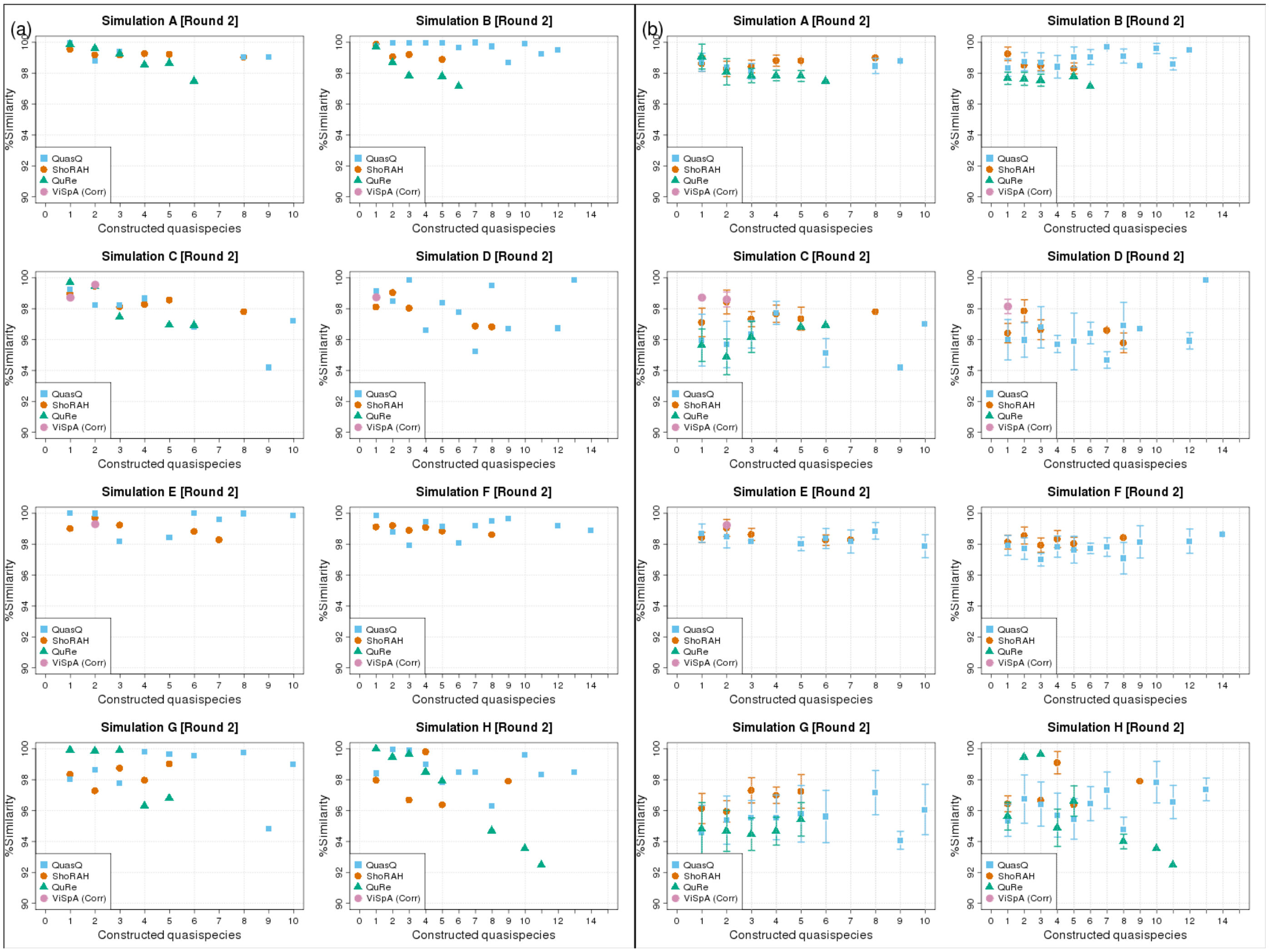

Supplementary Figure 6. (a) Degree of similarity between the best reconstructed haplotype sequences from QuasQ (blue squares), ShoRAH (red diamonds), QuRe (green triangles) and ViSpA with ShoRAH corrected reads (pink circle) with the actual simulated sequences. Here we identified the most similar reconstructed sequence to the simulated variants. (b) This shows the mean and standard deviation of the similarity for the reconstructed sequences that are mapped to a particular simulated variant. The above figures are based on the data from the second round of the simulations.

Supplementary Figure 7.

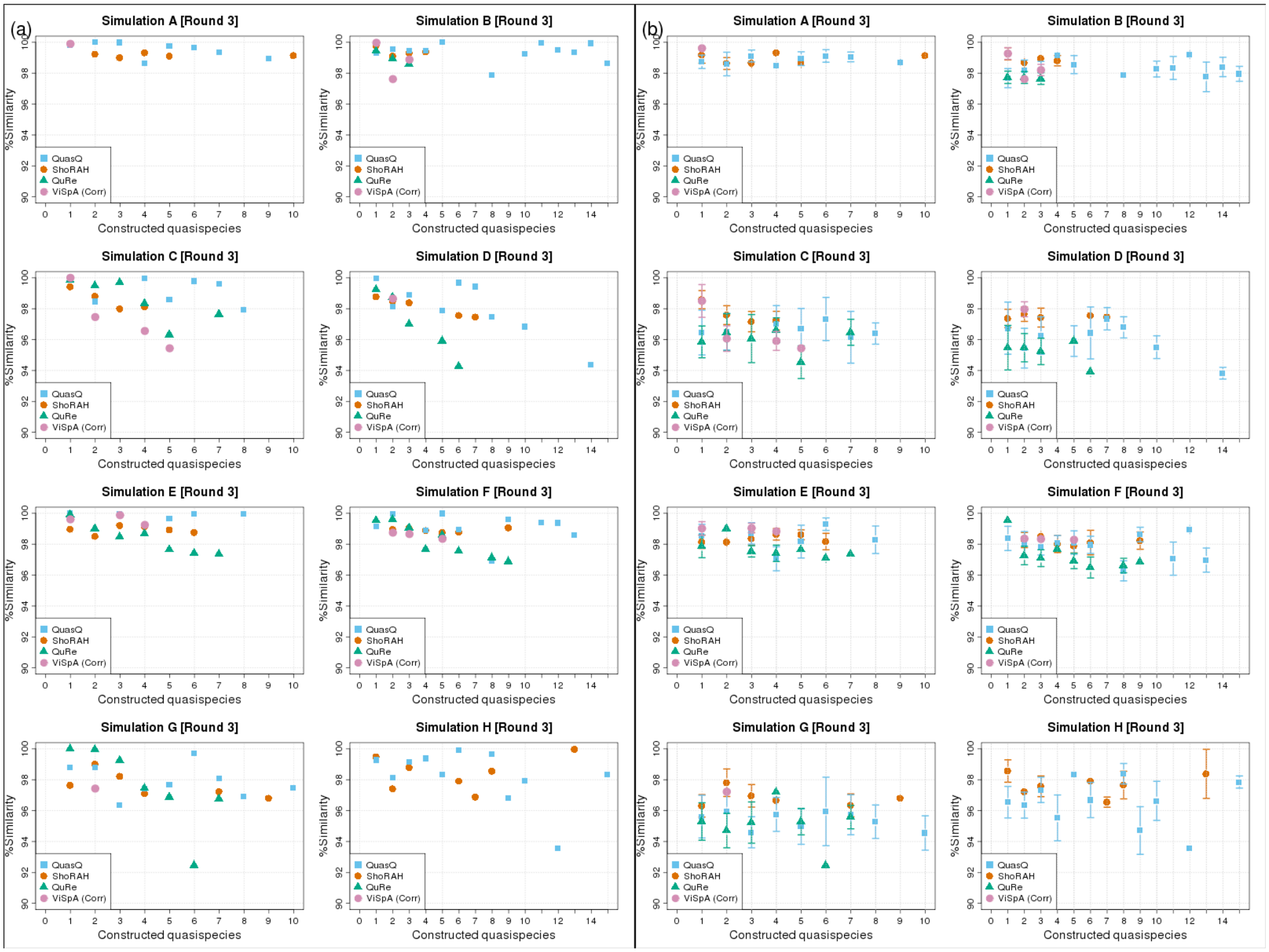

Supplementary Figure 7. (a) Degree of similarity between the best reconstructed haplotype sequences from QuasQ (blue squares), ShoRAH (red diamonds), QuRe (green triangles) and ViSpA with ShoRAH corrected reads (pink circle) with the actual simulated sequences. Here we identified the most similar reconstructed sequence to the simulated variants. (b) This shows the mean and standard deviation of the similarity for the reconstructed sequences that are mapped to a particular simulated variant. The above figures are based on the data from the third round of the simulations.

Supplementary Figure 8.

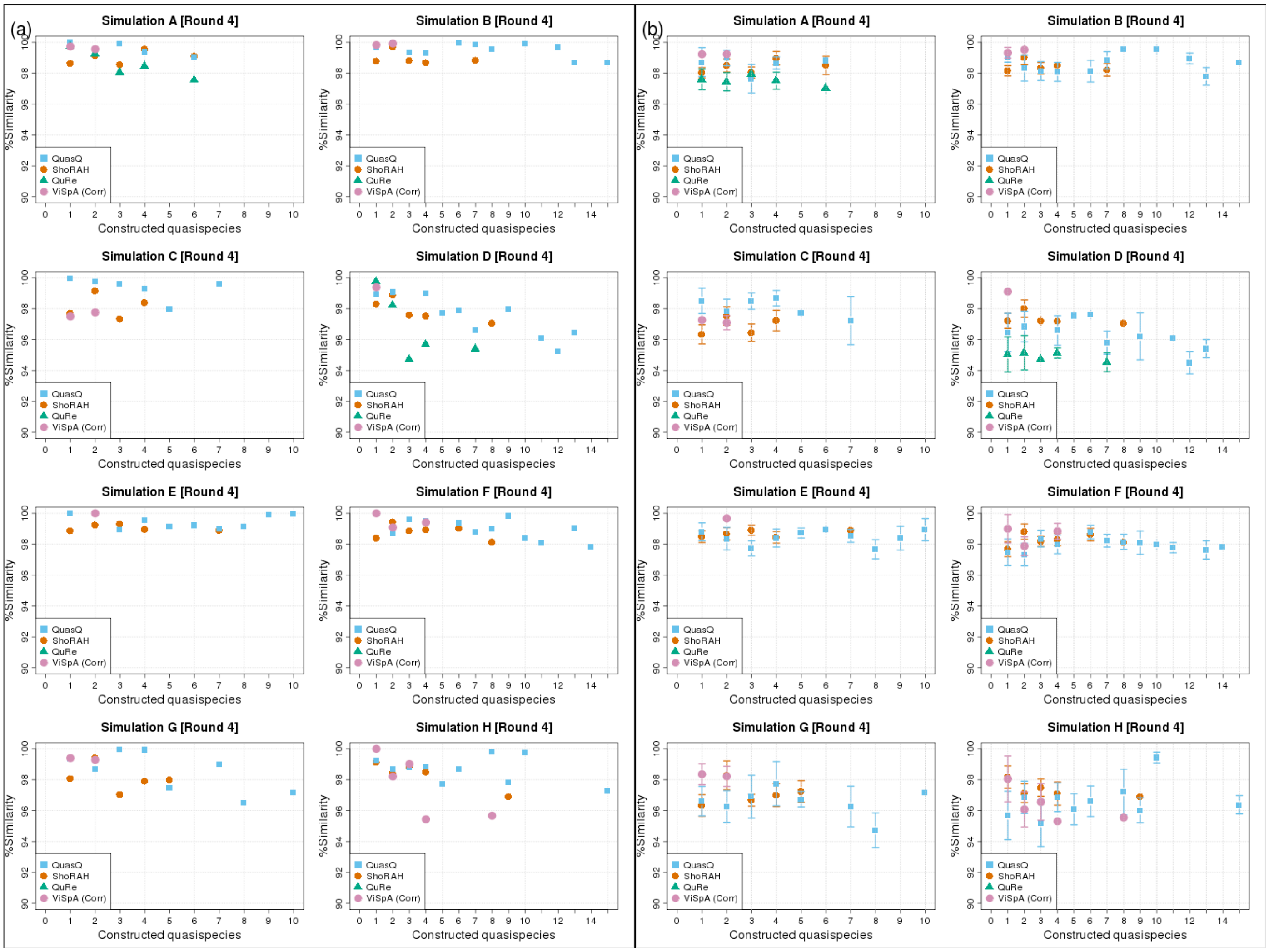

Supplementary Figure 8. (a) Degree of similarity between the best reconstructed haplotype sequences from QuasQ (blue squares), ShoRAH (red diamonds), QuRe (green triangles) and ViSpA with ShoRAH corrected reads (pink circle) with the actual simulated sequences. Here we identified the most similar reconstructed sequence to the simulated variants. (b) This shows the mean and standard deviation of the similarity for the reconstructed sequences that are mapped to a particular simulated variant. The above figures are based on the data from the fourth round of the simulations.

Supplementary Figure 9.

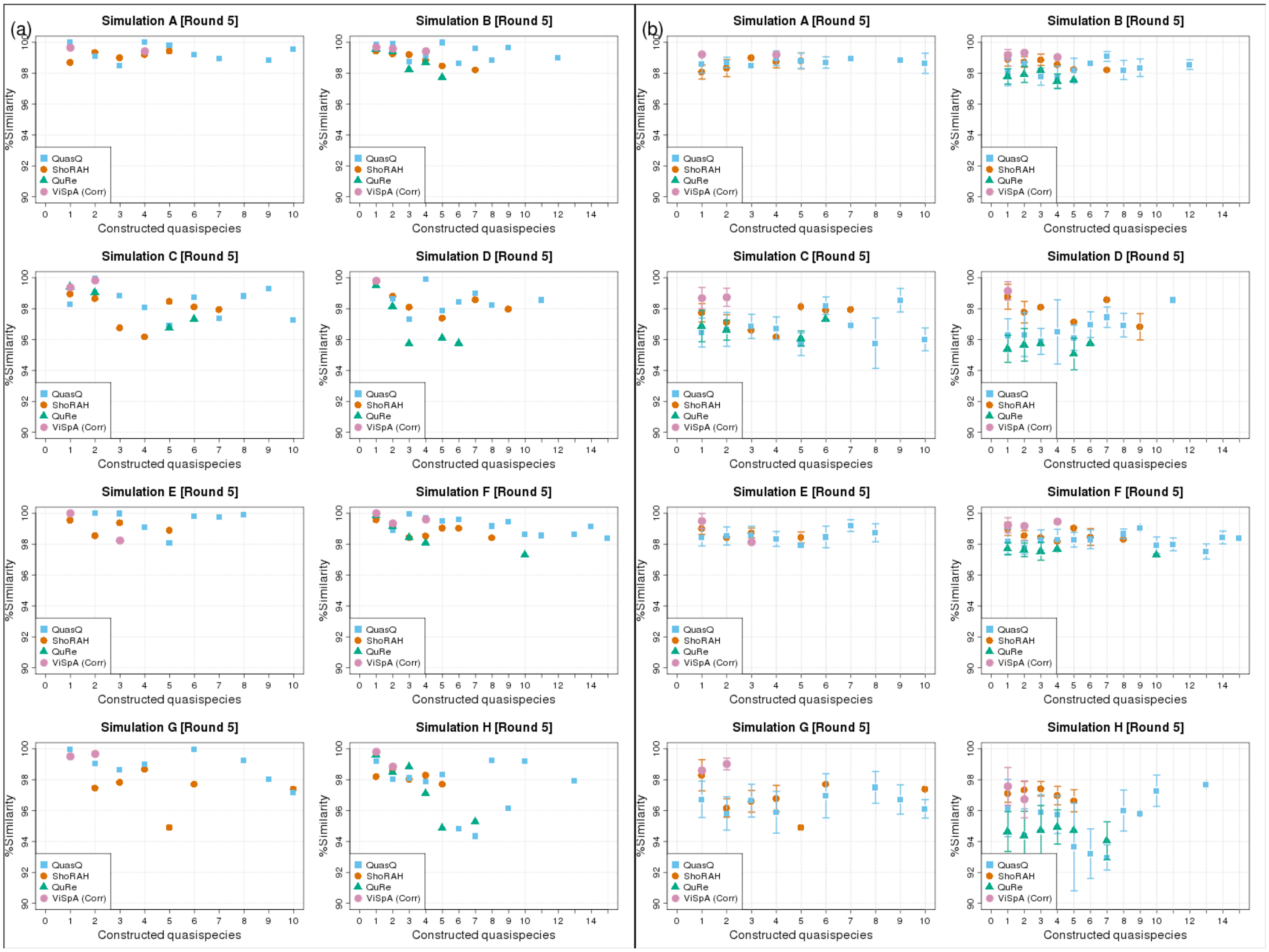

Supplementary Figure 9. (a) Degree of similarity between the best reconstructed haplotype sequences from QuasQ (blue squares), ShoRAH (red diamonds), QuRe (green triangles) and ViSpA with ShoRAH corrected reads (pink circle) with the actual simulated sequences. Here we identified the most similar reconstructed sequence to the simulated variants. (b) This shows the mean and standard deviation of the similarity for the reconstructed sequences that are mapped to a particular simulated variant. The above figures are based on the data from the fifth round of the simulations.

Supplementary Figure 10.

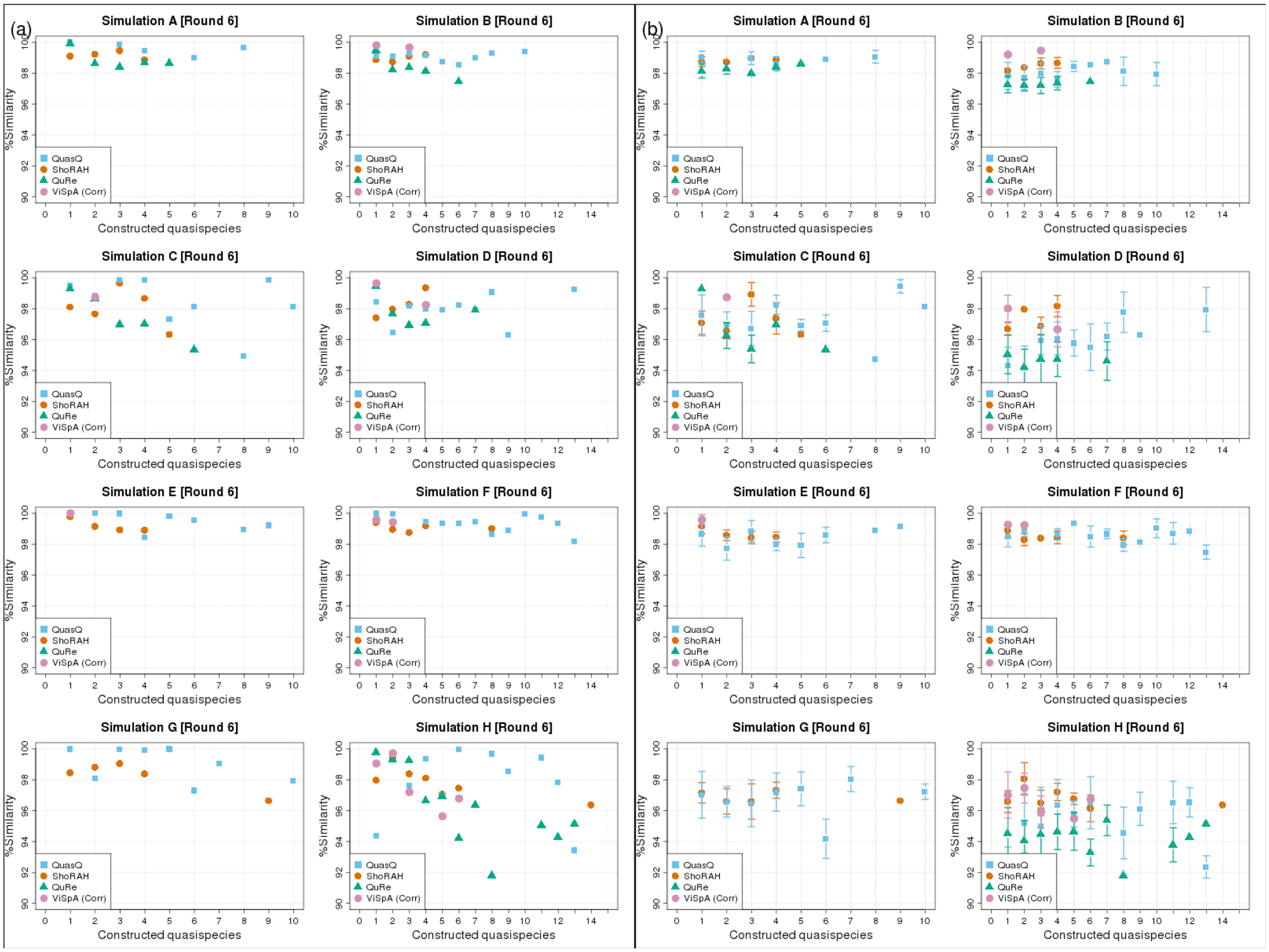

Supplementary Figure 10. (a) Degree of similarity between the best reconstructed haplotype sequences from QuasQ (blue squares), ShoRAH (red diamonds), QuRe (green triangles) and ViSpA with ShoRAH corrected reads (pink circle) with the actual simulated sequences. Here we identified the most similar reconstructed sequence to the simulated variants. (b) This shows the mean and standard deviation of the similarity for the reconstructed sequences that are mapped to a particular simulated variant. The above figures are based on the data from the sixth round of the simulations.

Supplementary Figure 11.

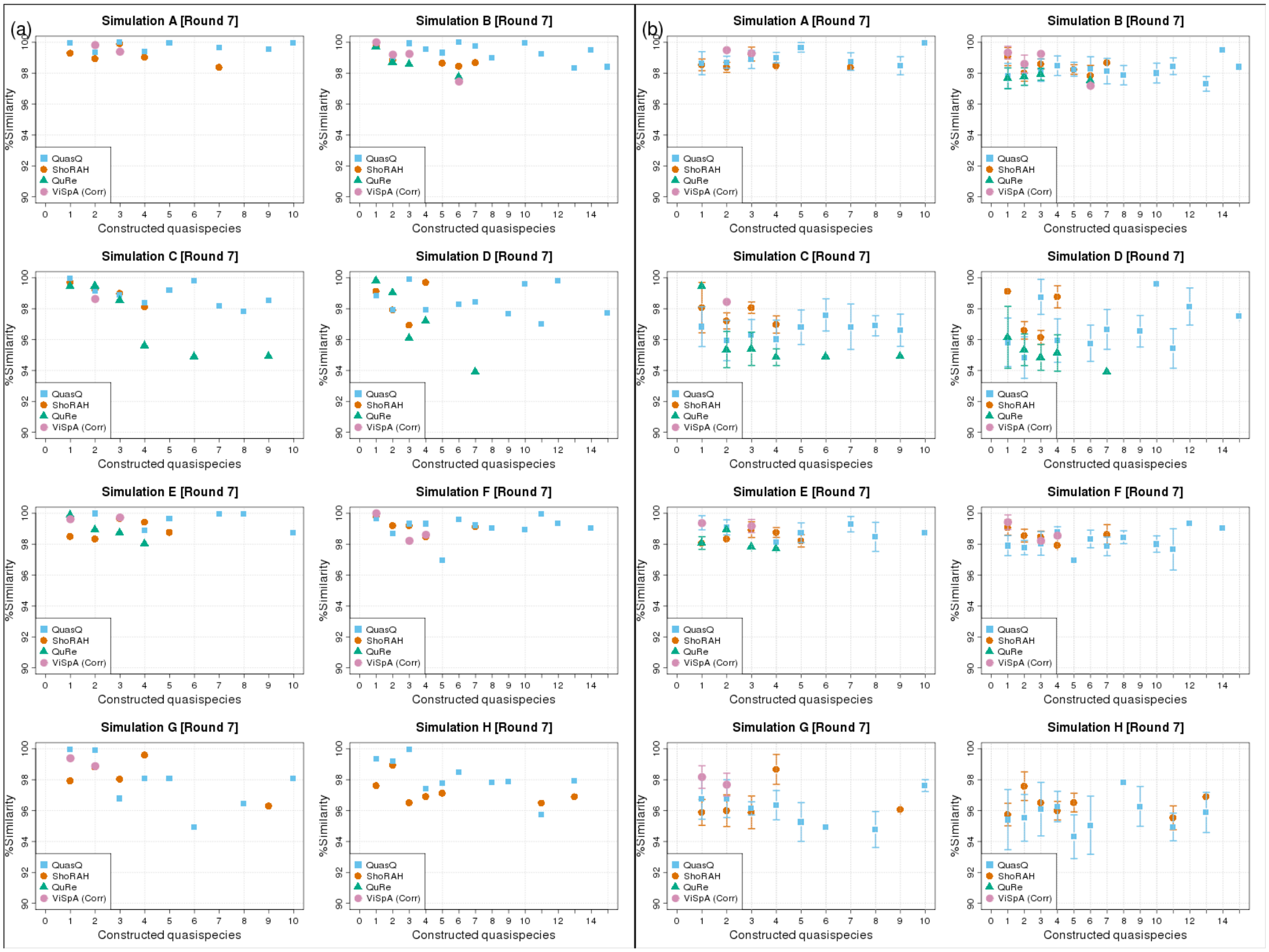

Supplementary Figure 11. (a) Degree of similarity between the best reconstructed haplotype sequences from QuasQ (blue squares), ShoRAH (red diamonds), QuRe (green triangles) and ViSpA with ShoRAH corrected reads (pink circle) with the actual simulated sequences. Here we identified the most similar reconstructed sequence to the simulated variants. (b) This shows the mean and standard deviation of the similarity for the reconstructed sequences that are mapped to a particular simulated variant. The above figures are based on the data from the seventh round of the simulations.

Supplementary Figure 12.

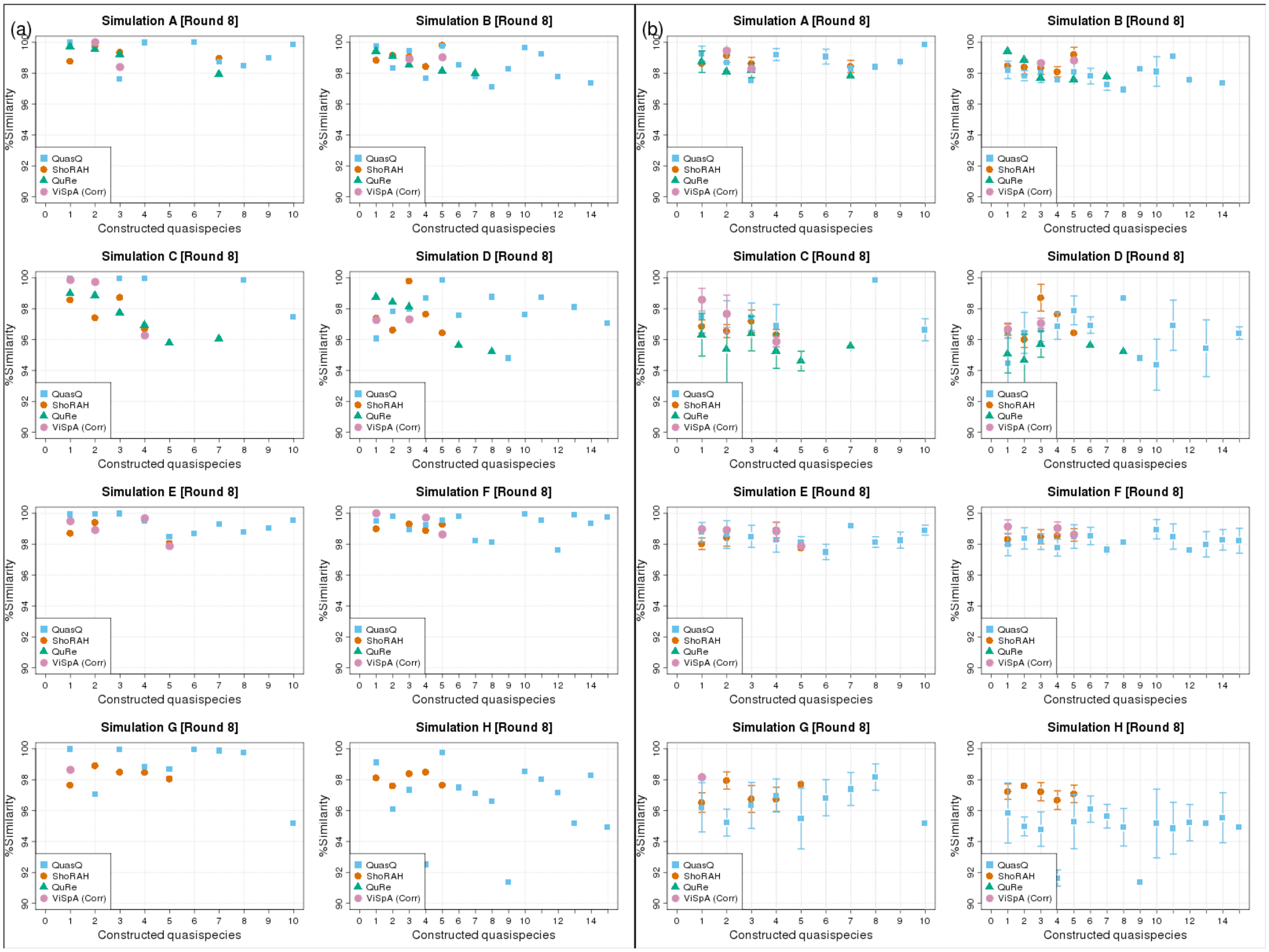

Supplementary Figure 12. (a) Degree of similarity between the best reconstructed haplotype sequences from QuasQ (blue squares), ShoRAH (red diamonds), QuRe (green triangles) and ViSpA with ShoRAH corrected reads (pink circle) with the actual simulated sequences. Here we identified the most similar reconstructed sequence to the simulated variants. (b) This shows the mean and standard deviation of the similarity for the reconstructed sequences that are mapped to a particular simulated variant. The above figures are based on the data from the eighth round of the simulations.

Supplementary Figure 13.

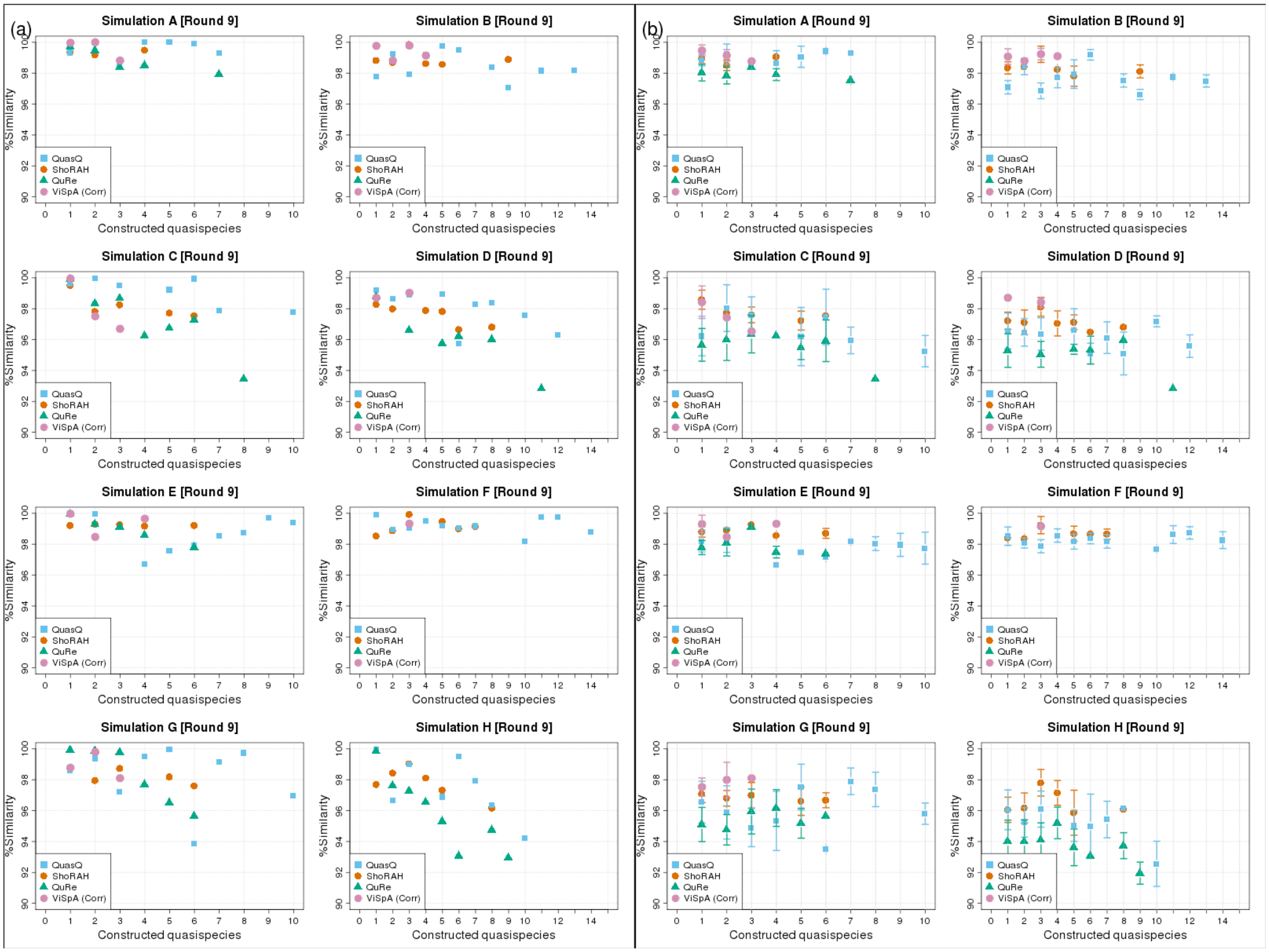

Supplementary Figure 13. (a) Degree of similarity between the best reconstructed haplotype sequences from QuasQ (blue squares), ShoRAH (red diamonds), QuRe (green triangles) and ViSpA with ShoRAH corrected reads (pink circle) with the actual simulated sequences. Here we identified the most similar reconstructed sequence to the simulated variants. (b) This shows the mean and standard deviation of the similarity for the reconstructed sequences that are mapped to a particular simulated variant. The above figures are based on the data from the ninth round of the simulations.

Supplementary Figure 14.

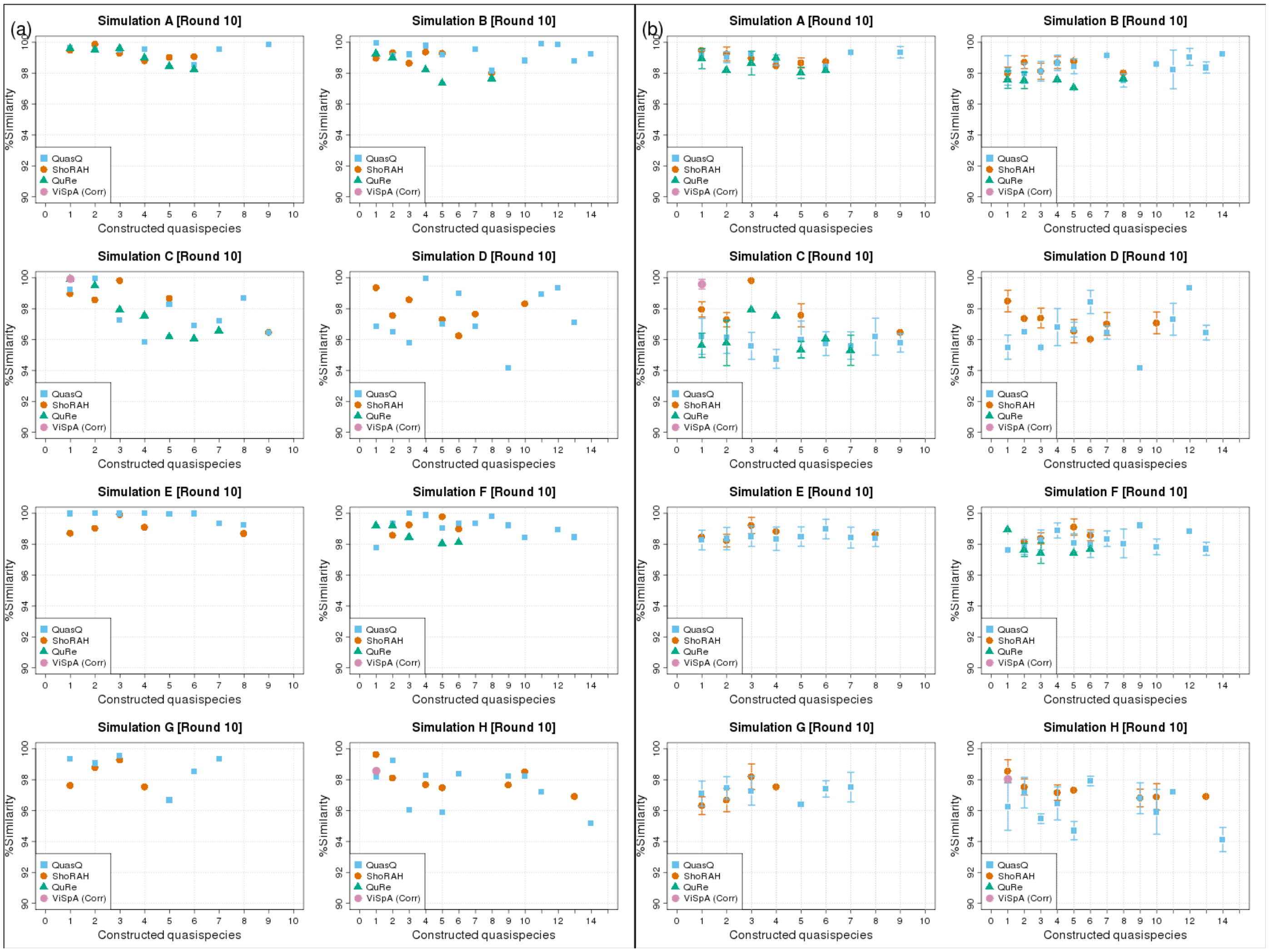

Supplementary Figure 14. (a) Degree of similarity between the best reconstructed haplotype sequences from QuasQ (blue squares), ShoRAH (red diamonds), QuRe (green triangles) and ViSpA with ShoRAH corrected reads (pink circle) with the actual simulated sequences. Here we identified the most similar reconstructed sequence to the simulated variants. (b) This shows the mean and standard deviation of the similarity for the reconstructed sequences that are mapped to a particular simulated variant. The above figures are based on the data from the tenth round of the simulations.

Supplementary Figure 15.

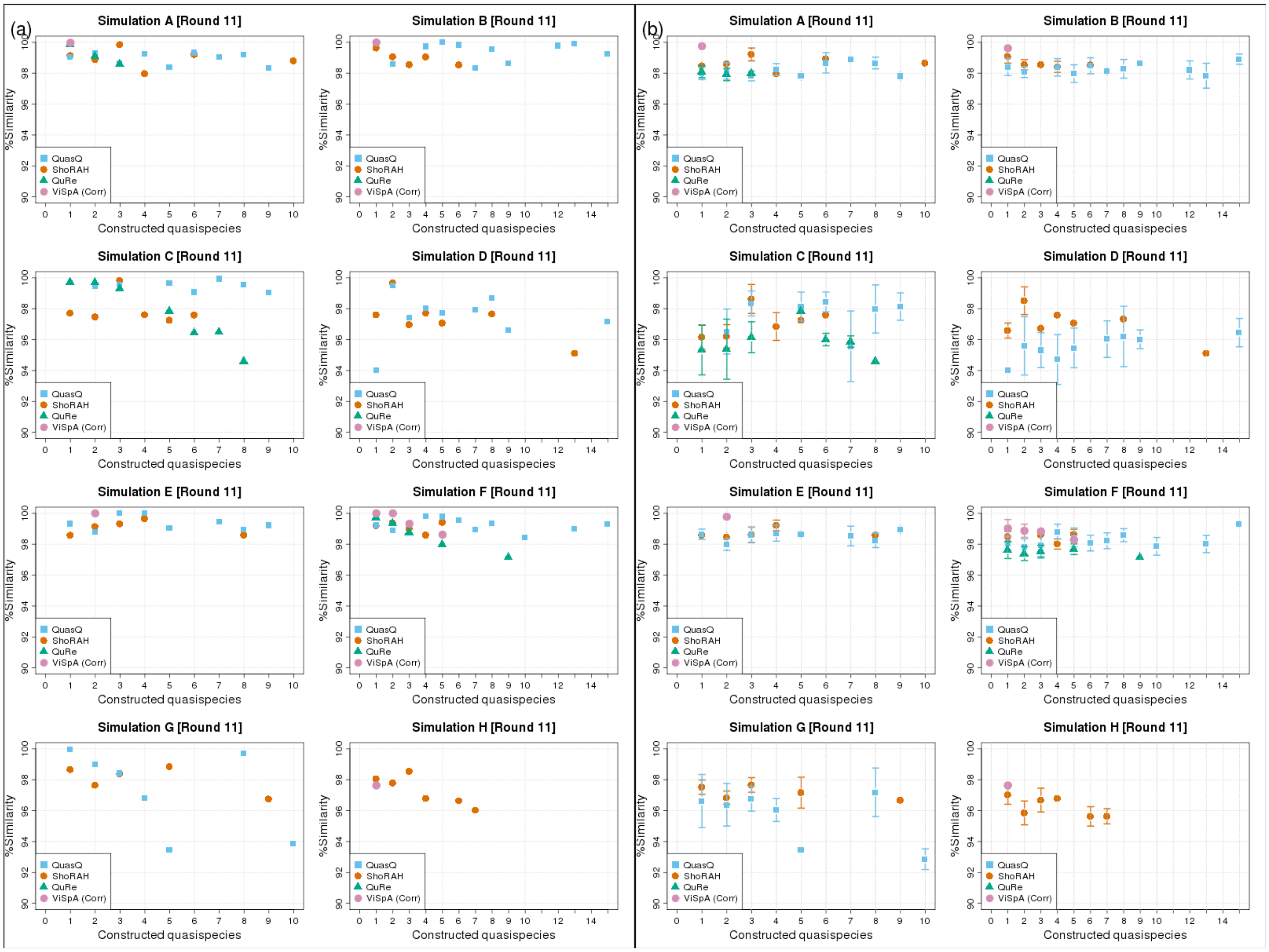

Supplementary Figure 15. (a) Degree of similarity between the best reconstructed haplotype sequences from QuasQ (blue squares), ShoRAH (red diamonds), QuRe (green triangles) and ViSpA with ShoRAH corrected reads (pink circle) with the actual simulated sequences. Here we identified the most similar reconstructed sequence to the simulated variants. (b) This shows the mean and standard deviation of the similarity for the reconstructed sequences that are mapped to a particular simulated variant. The above figures are based on the data from the 11th round of the simulations.

Supplementary Figure 16.

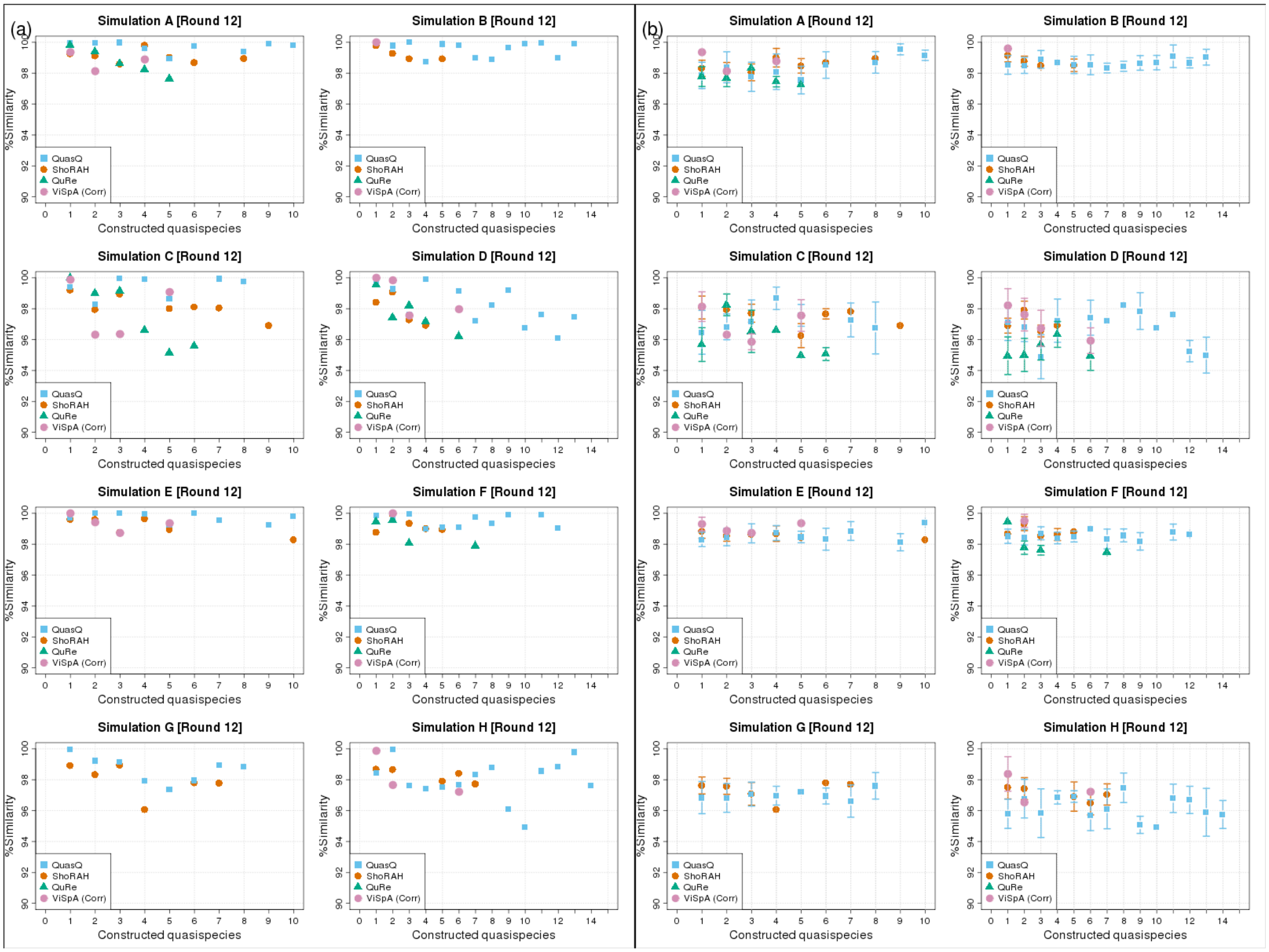

Supplementary Figure 16. (a) Degree of similarity between the best reconstructed haplotype sequences from QuasQ (blue squares), ShoRAH (red diamonds), QuRe (green triangles) and ViSpA with ShoRAH corrected reads (pink circle) with the actual simulated sequences. Here we identified the most similar reconstructed sequence to the simulated variants. (b) This shows the mean and standard deviation of the similarity for the reconstructed sequences that are mapped to a particular simulated variant. The above figures are based on the data from the 12th round of the simulations.

Supplementary Figure 17.

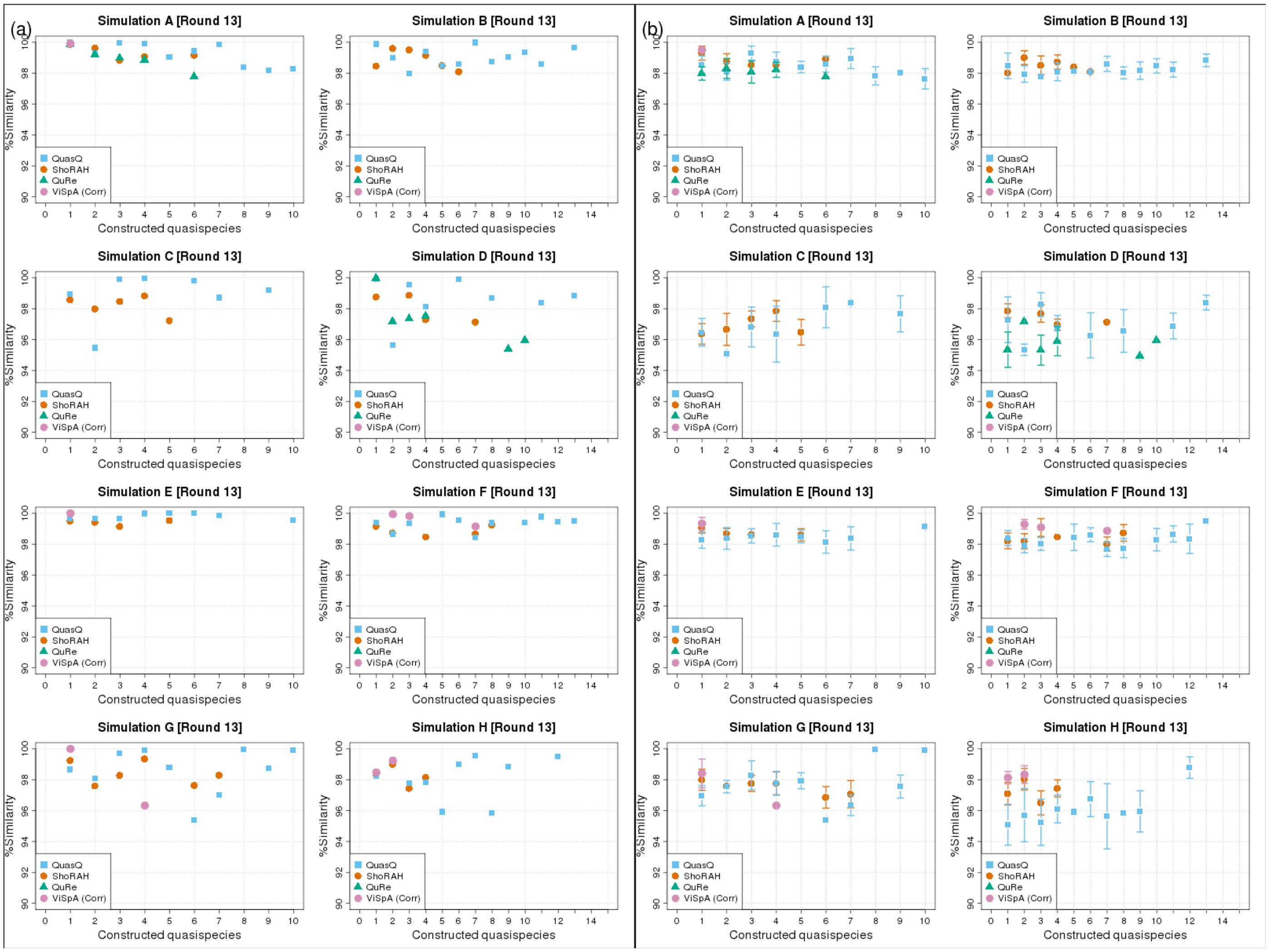

Supplementary Figure 17. (a) Degree of similarity between the best reconstructed haplotype sequences from QuasQ (blue squares), ShoRAH (red diamonds), QuRe (green triangles) and ViSpA with ShoRAH corrected reads (pink circle) with the actual simulated sequences. Here we identified the most similar reconstructed sequence to the simulated variants. (b) This shows the mean and standard deviation of the similarity for the reconstructed sequences that are mapped to a particular simulated variant. The above figures are based on the data from the 13th round of the simulations.

Supplementary Figure 18.

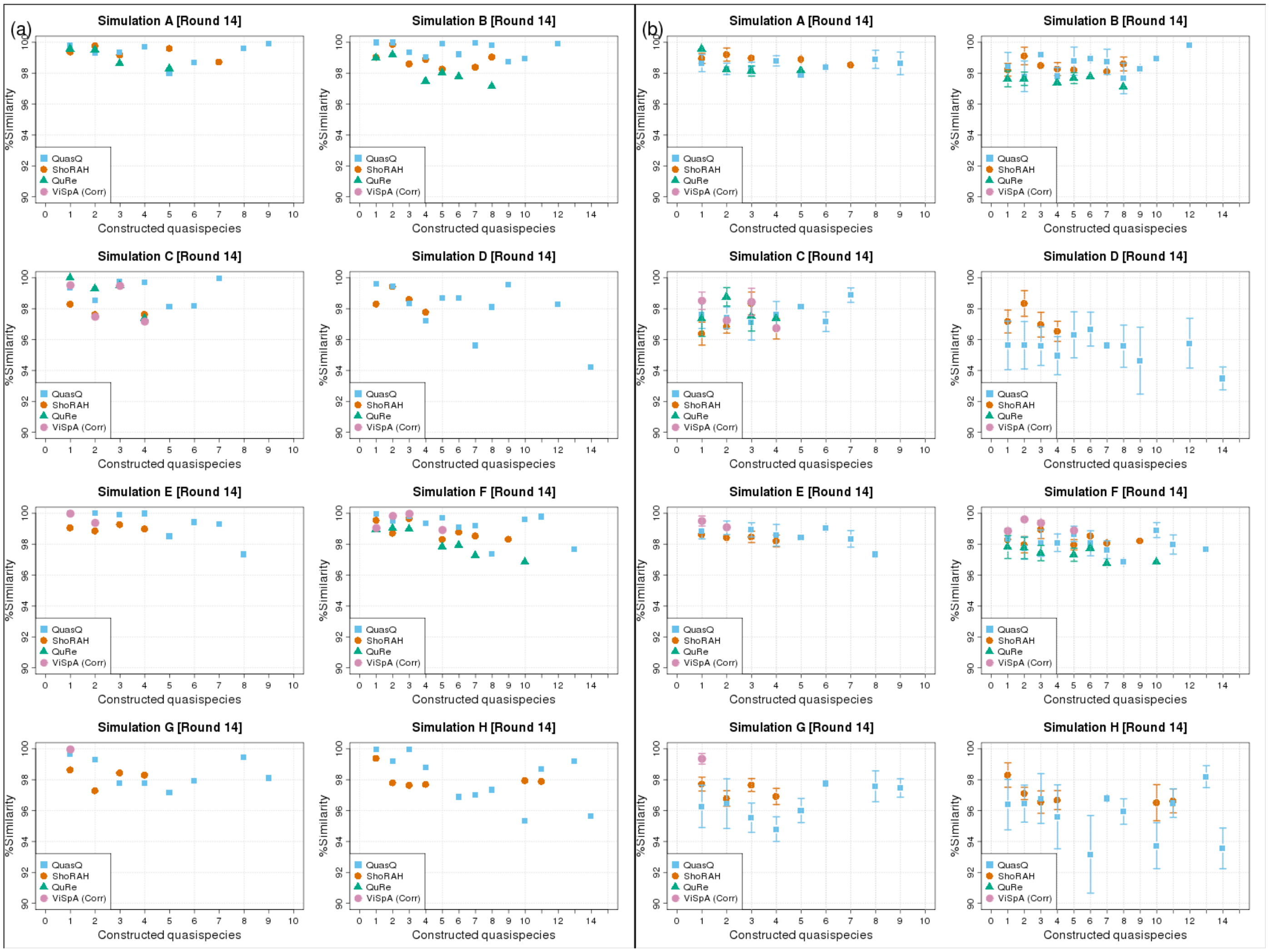

Supplementary Figure 18. (a) Degree of similarity between the best reconstructed haplotype sequences from QuasQ (blue squares), ShoRAH (red diamonds), QuRe (green triangles) and ViSpA with ShoRAH corrected reads (pink circle) with the actual simulated sequences. Here we identified the most similar reconstructed sequence to the simulated variants. (b) This shows the mean and standard deviation of the similarity for the reconstructed sequences that are mapped to a particular simulated variant. The above figures are based on the data from the 14th round of the simulations.

Supplementary Figure 19.

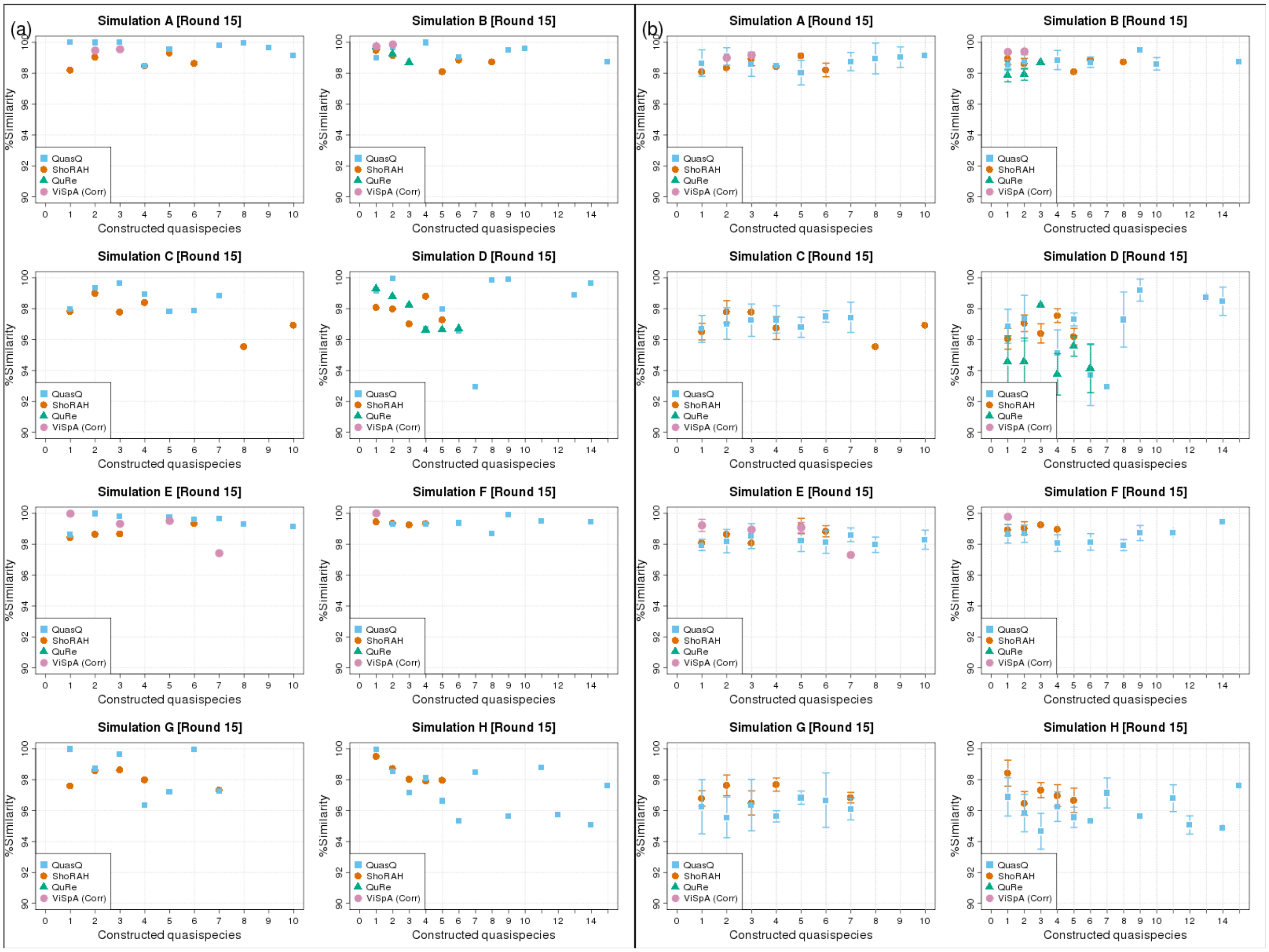

Supplementary Figure 19. (a) Degree of similarity between the best reconstructed haplotype sequences from QuasQ (blue squares), ShoRAH (red diamonds), QuRe (green triangles) and ViSpA with ShoRAH corrected reads (pink circle) with the actual simulated sequences. Here we identified the most similar reconstructed sequence to the simulated variants. (b) This shows the mean and standard deviation of the similarity for the reconstructed sequences that are mapped to a particular simulated variant. The above figures are based on the data from the 15th round of the simulations.

Supplementary Figure 20.

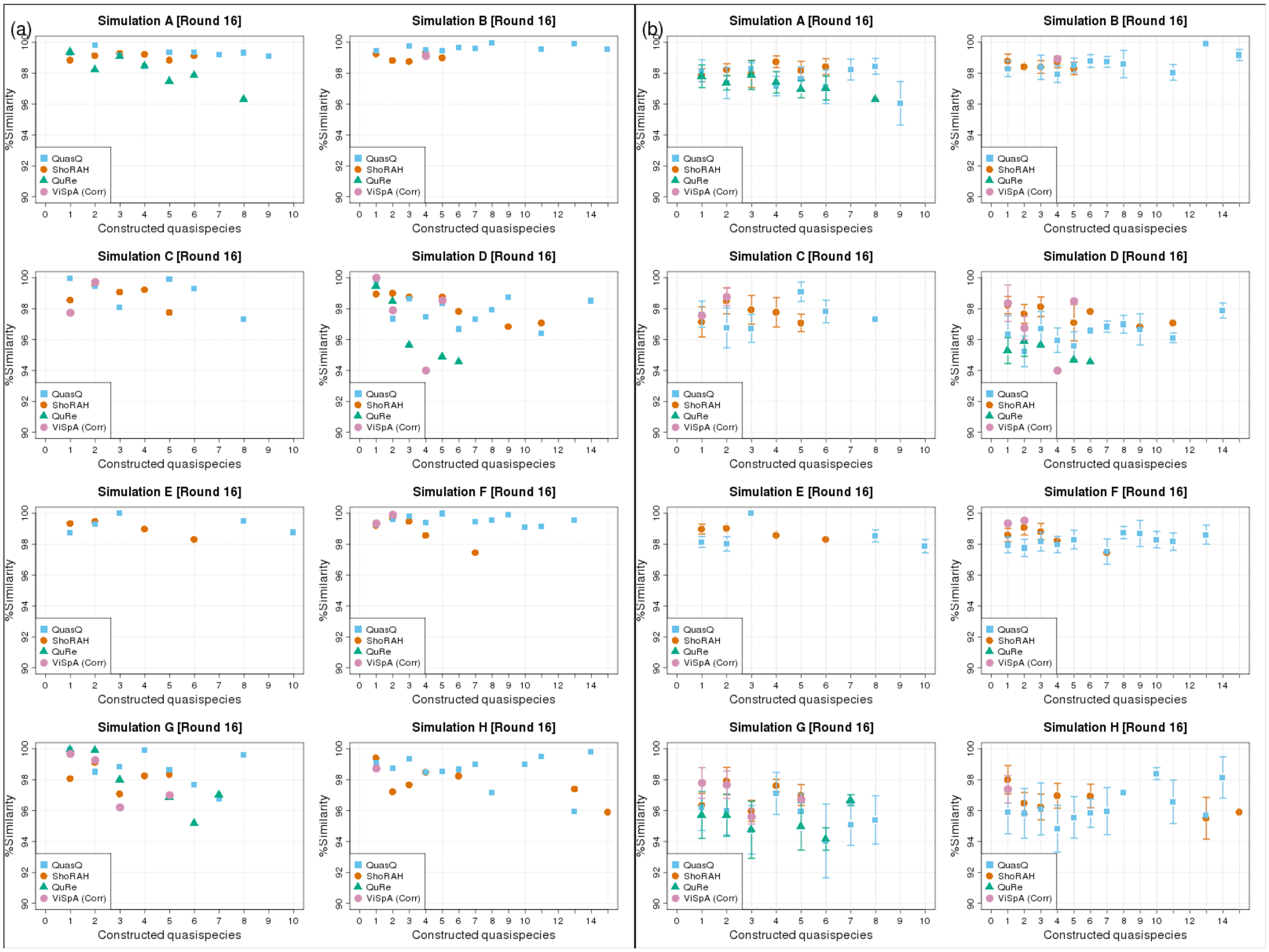

Supplementary Figure 20. (a) Degree of similarity between the best reconstructed haplotype sequences from QuasQ (blue squares), ShoRAH (red diamonds), QuRe (green triangles) and ViSpA with ShoRAH corrected reads (pink circle) with the actual simulated sequences. Here we identified the most similar reconstructed sequence to the simulated variants. (b) This shows the mean and standard deviation of the similarity for the reconstructed sequences that are mapped to a particular simulated variant. The above figures are based on the data from the 16th round of the simulations.

Supplementary Figure 21.

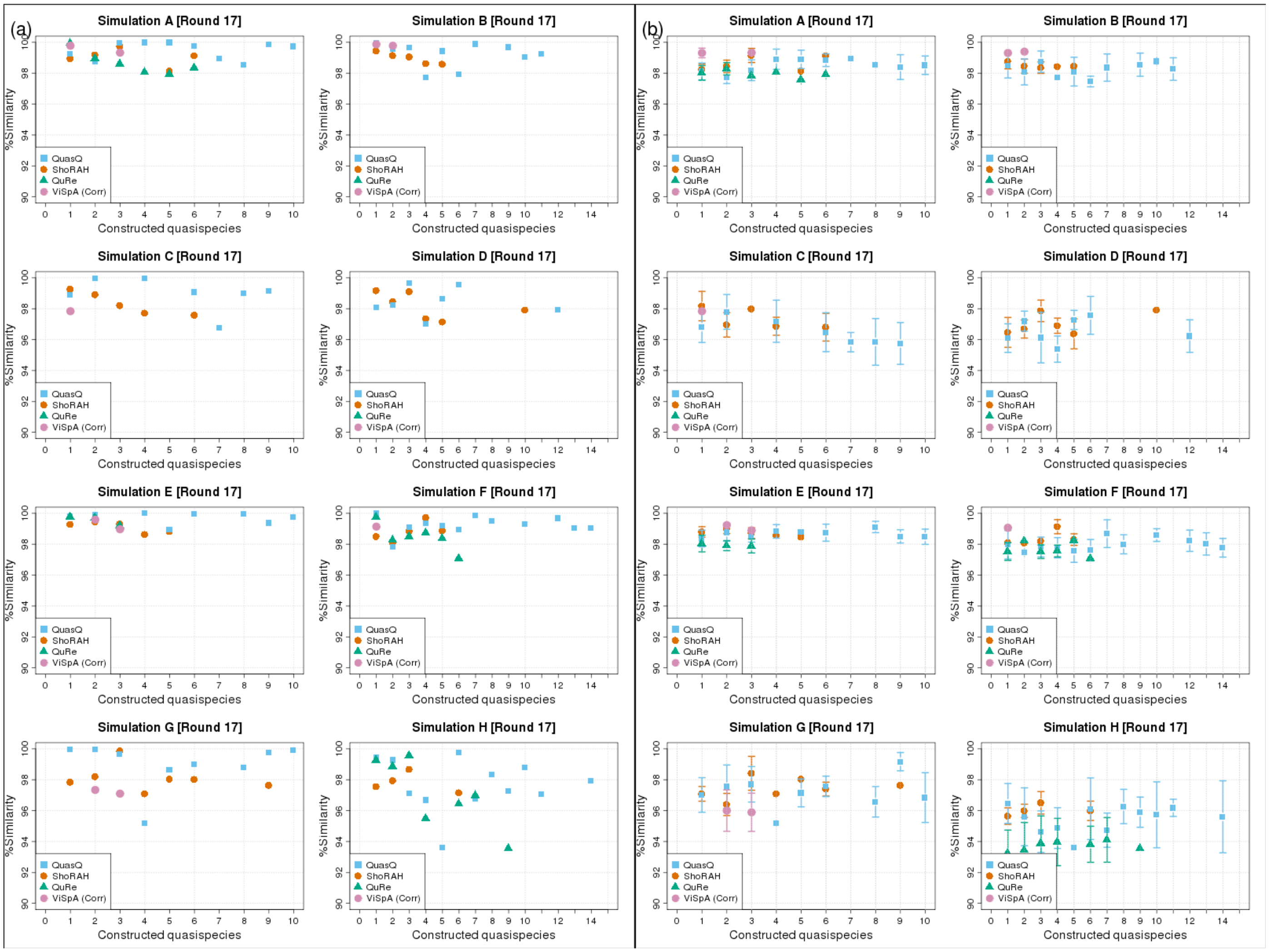

Supplementary Figure 21. (a) Degree of similarity between the best reconstructed haplotype sequences from QuasQ (blue squares), ShoRAH (red diamonds), QuRe (green triangles) and ViSpA with ShoRAH corrected reads (pink circle) with the actual simulated sequences. Here we identified the most similar reconstructed sequence to the simulated variants. (b) This shows the mean and standard deviation of the similarity for the reconstructed sequences that are mapped to a particular simulated variant. The above figures are based on the data from the 17th round of the simulations.

Supplementary Figure 22.

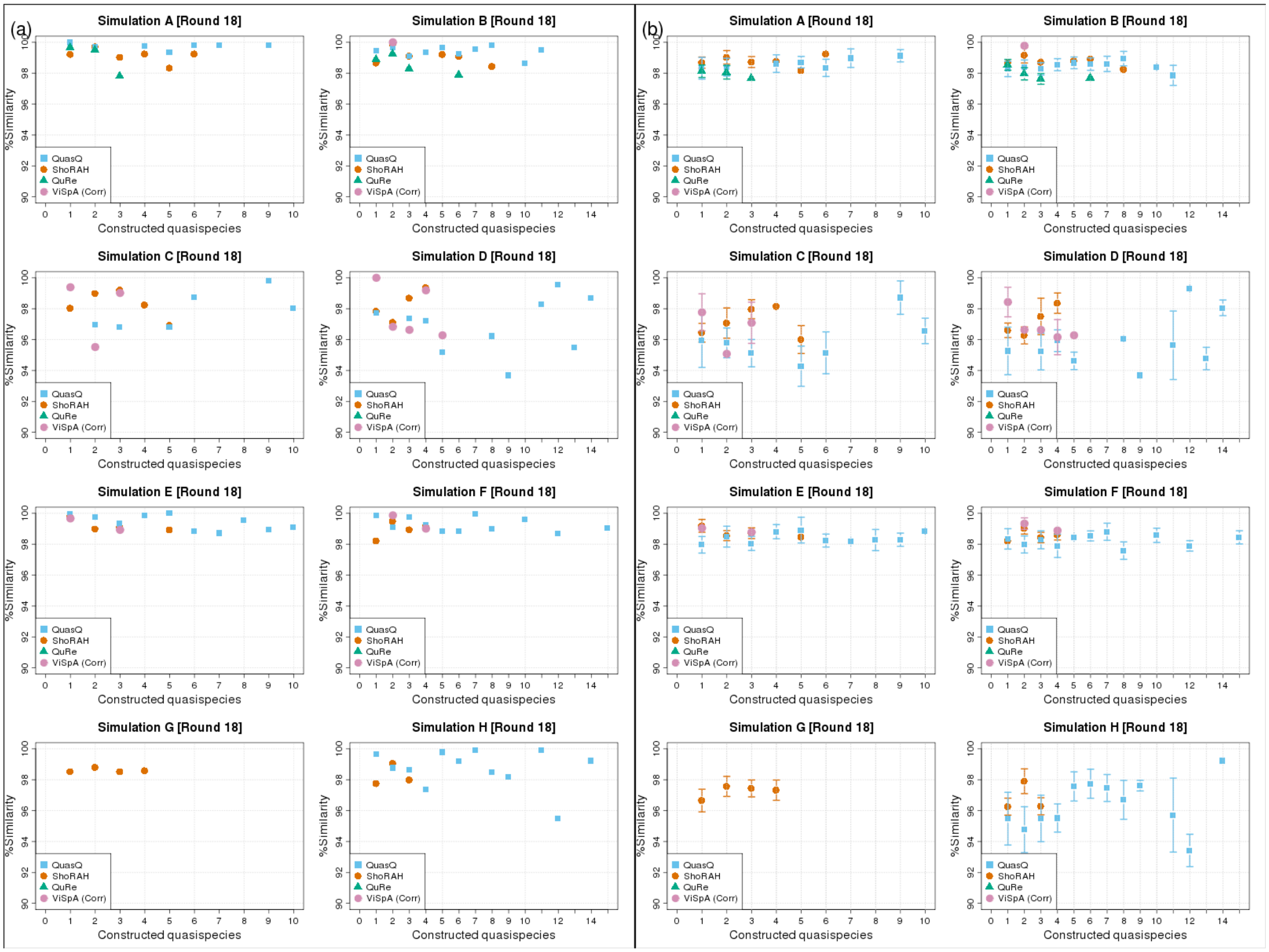

Supplementary Figure 22. (a) Degree of similarity between the best reconstructed haplotype sequences from QuasQ (blue squares), ShoRAH (red diamonds), QuRe (green triangles) and ViSpA with ShoRAH corrected reads (pink circle) with the actual simulated sequences. Here we identified the most similar reconstructed sequence to the simulated variants. (b) This shows the mean and standard deviation of the similarity for the reconstructed sequences that are mapped to a particular simulated variant. The above figures are based on the data from the 18th round of the simulations.

Supplementary Figure 23.

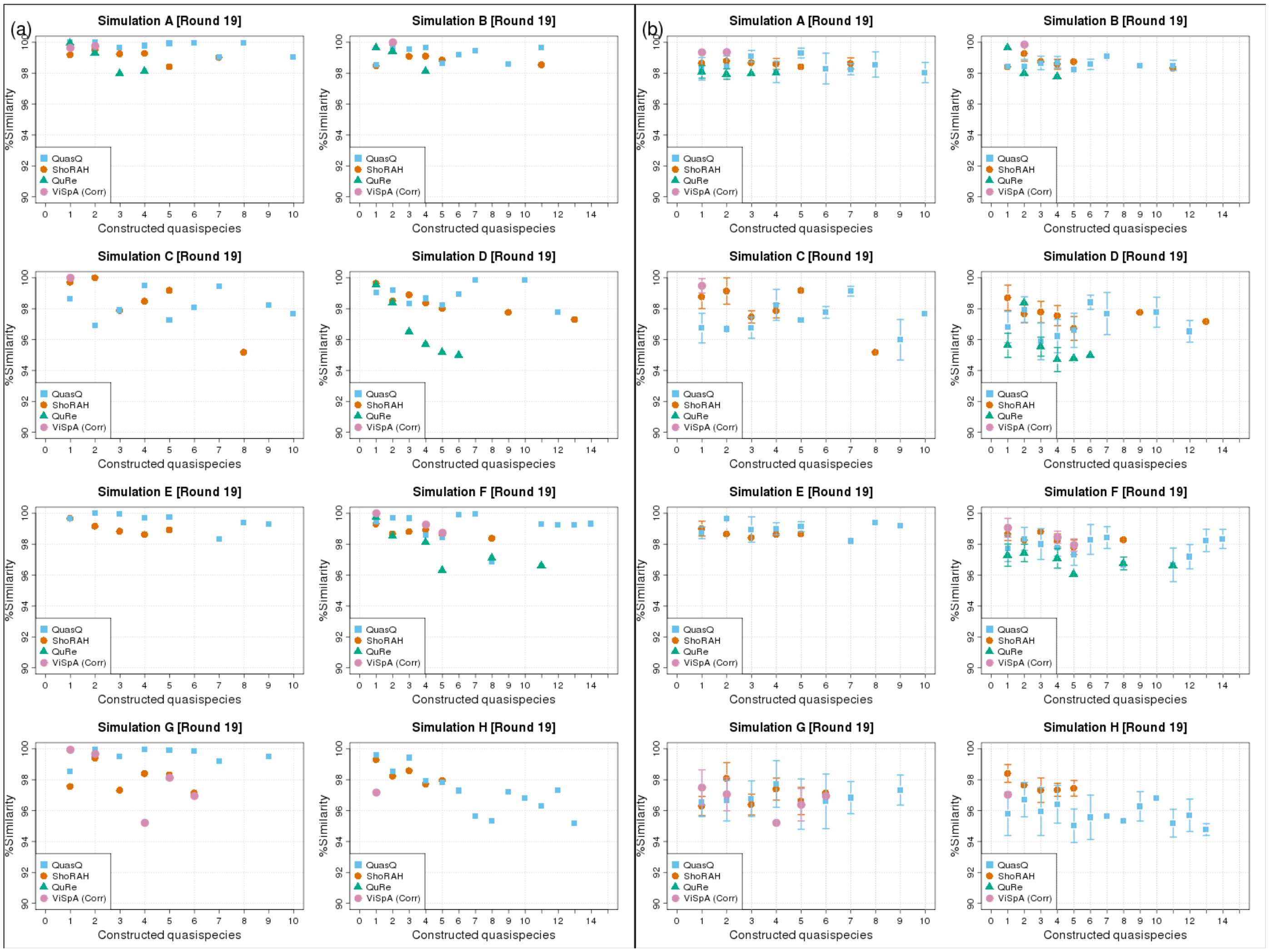

Supplementary Figure 23. (a) Degree of similarity between the best reconstructed haplotype sequences from QuasQ (blue squares), ShoRAH (red diamonds), QuRe (green triangles) and ViSpA with ShoRAH corrected reads (pink circle) with the actual simulated sequences. Here we identified the most similar reconstructed sequence to the simulated variants. (b) This shows the mean and standard deviation of the similarity for the reconstructed sequences that are mapped to a particular simulated variant. The above figures are based on the data from the 19th round of the simulations.

Supplementary Figure 24.

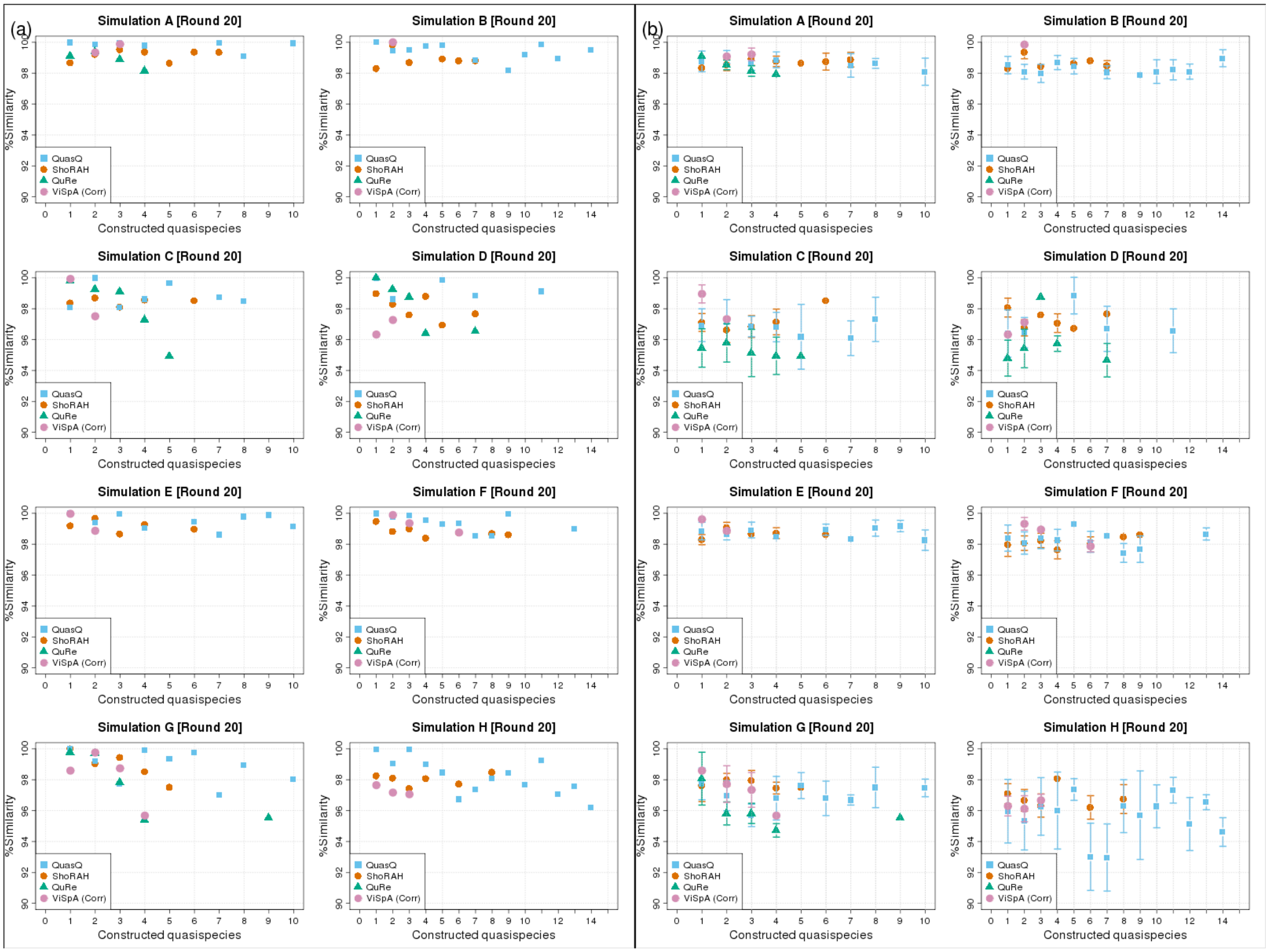

Supplementary Figure 24. (a) Degree of similarity between the best reconstructed haplotype sequences from QuasQ (blue squares), ShoRAH (red diamonds), QuRe (green triangles) and ViSpA with ShoRAH corrected reads (pink circle) with the actual simulated sequences. Here we identified the most similar reconstructed sequence to the simulated variants. (b) This shows the mean and standard deviation of the similarity for the reconstructed sequences that are mapped to a particular simulated variant. The above figures are based on the data from the 20th round of the simulations.

Supplementary Figure 25.

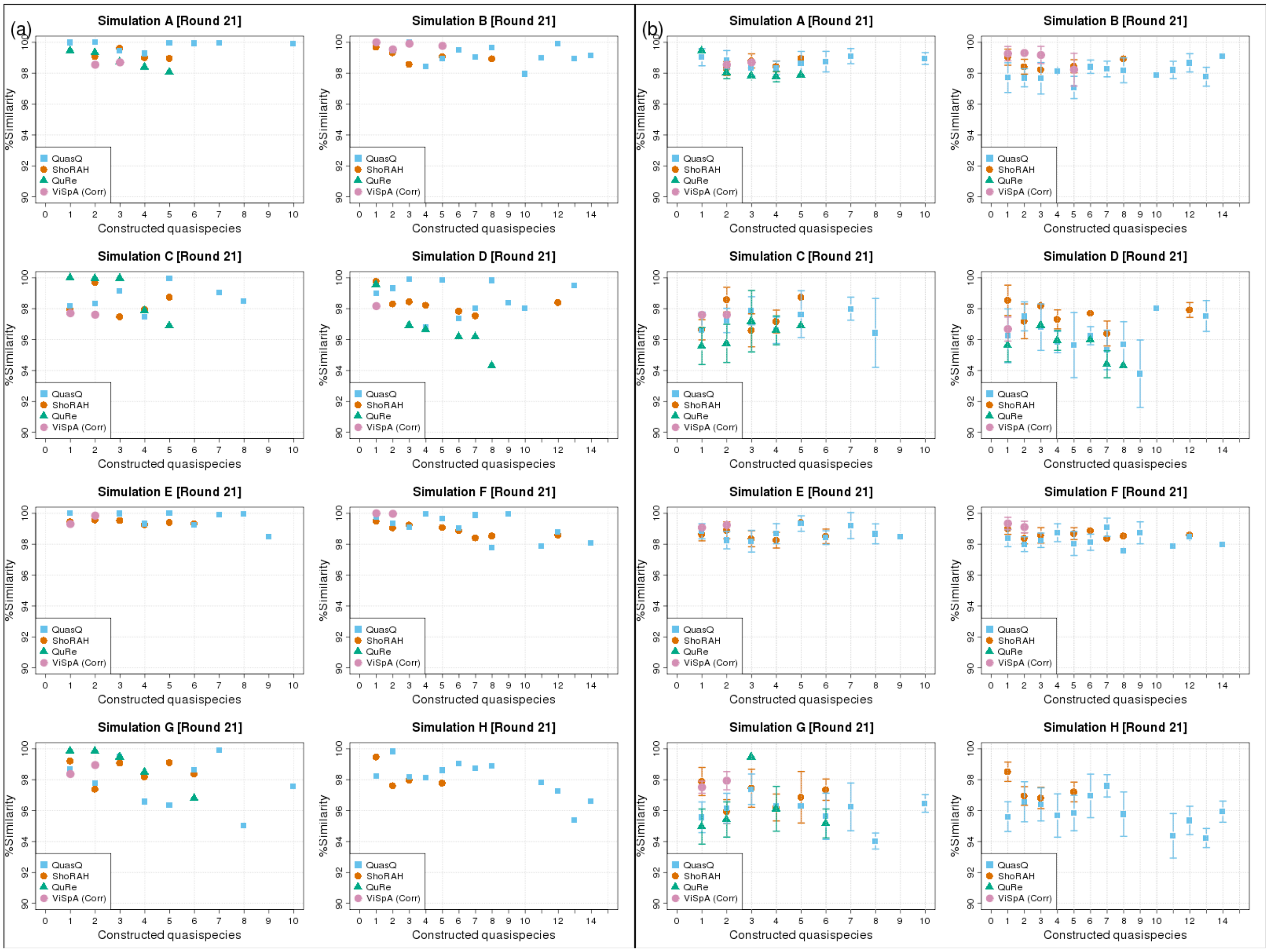

Supplementary Figure 25. (a) Degree of similarity between the best reconstructed haplotype sequences from QuasQ (blue squares), ShoRAH (red diamonds), QuRe (green triangles) and ViSpA with ShoRAH corrected reads (pink circle) with the actual simulated sequences. Here we identified the most similar reconstructed sequence to the simulated variants. (b) This shows the mean and standard deviation of the similarity for the reconstructed sequences that are mapped to a particular simulated variant. The above figures are based on the data from the 21st round of the simulations.

Supplementary Figure 26.

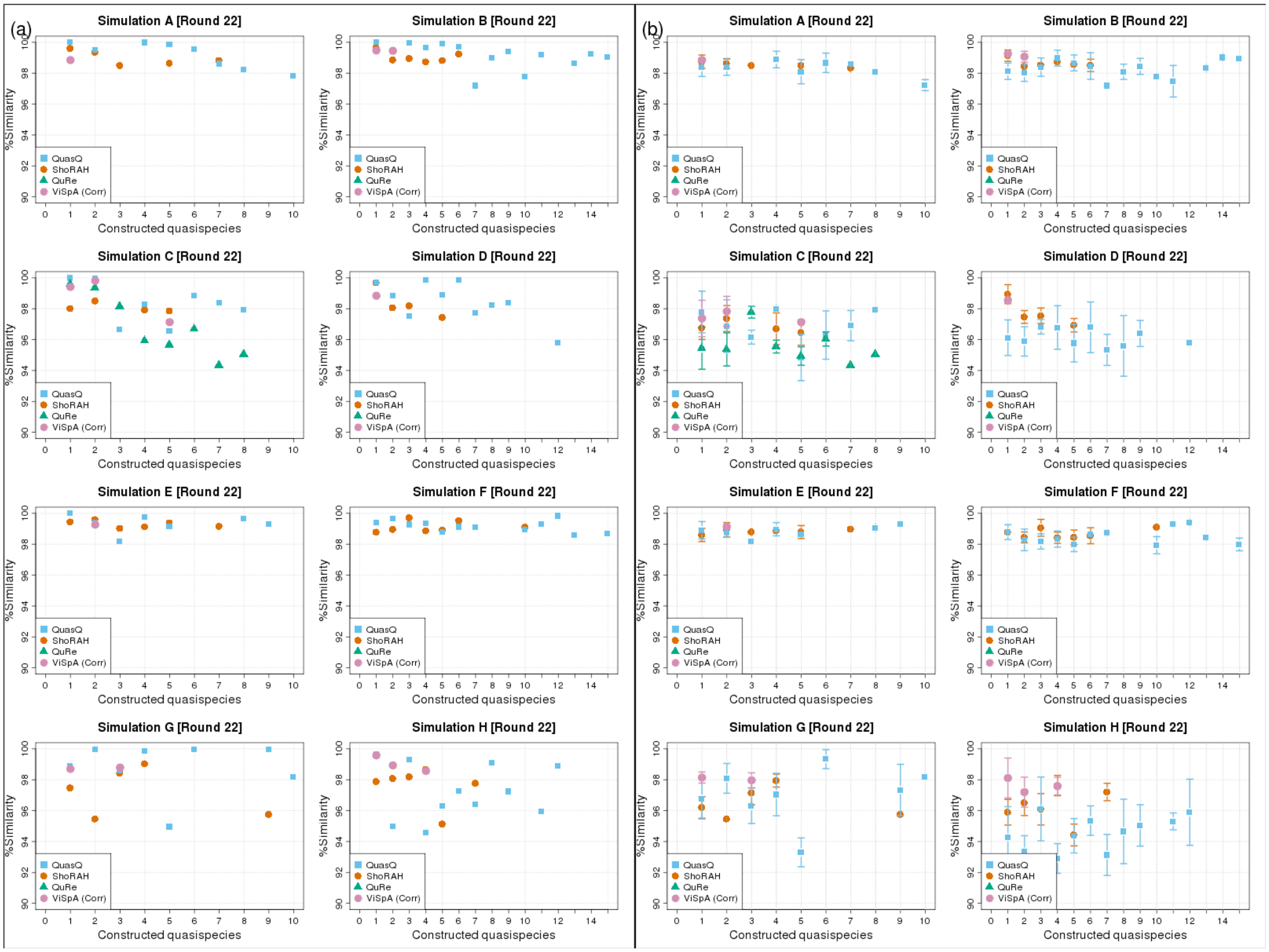

Supplementary Figure 26. (a) Degree of similarity between the best reconstructed haplotype sequences from QuasQ (blue squares), ShoRAH (red diamonds), QuRe (green triangles) and ViSpA with ShoRAH corrected reads (pink circle) with the actual simulated sequences. Here we identified the most similar reconstructed sequence to the simulated variants. (b) This shows the mean and standard deviation of the similarity for the reconstructed sequences that are mapped to a particular simulated variant. The above figures are based on the data from the 22nd round of the simulations.

Supplementary Figure 27.

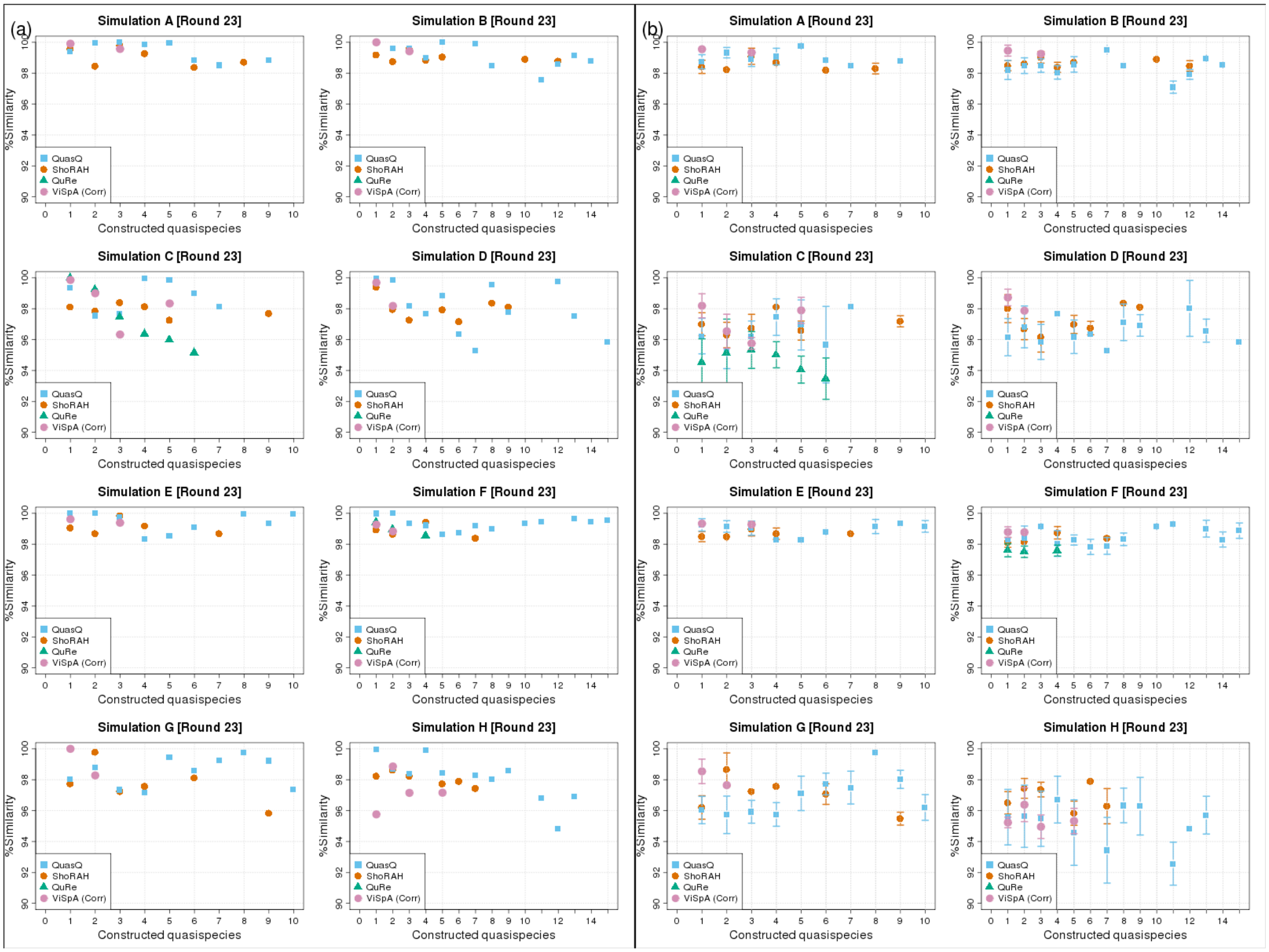

Supplementary Figure 27. (a) Degree of similarity between the best reconstructed haplotype sequences from QuasQ (blue squares), ShoRAH (red diamonds), QuRe (green triangles) and ViSpA with ShoRAH corrected reads (pink circle) with the actual simulated sequences. Here we identified the most similar reconstructed sequence to the simulated variants. (b) This shows the mean and standard deviation of the similarity for the reconstructed sequences that are mapped to a particular simulated variant. The above figures are based on the data from the 23rd round of the simulations.

Supplementary Figure 28.

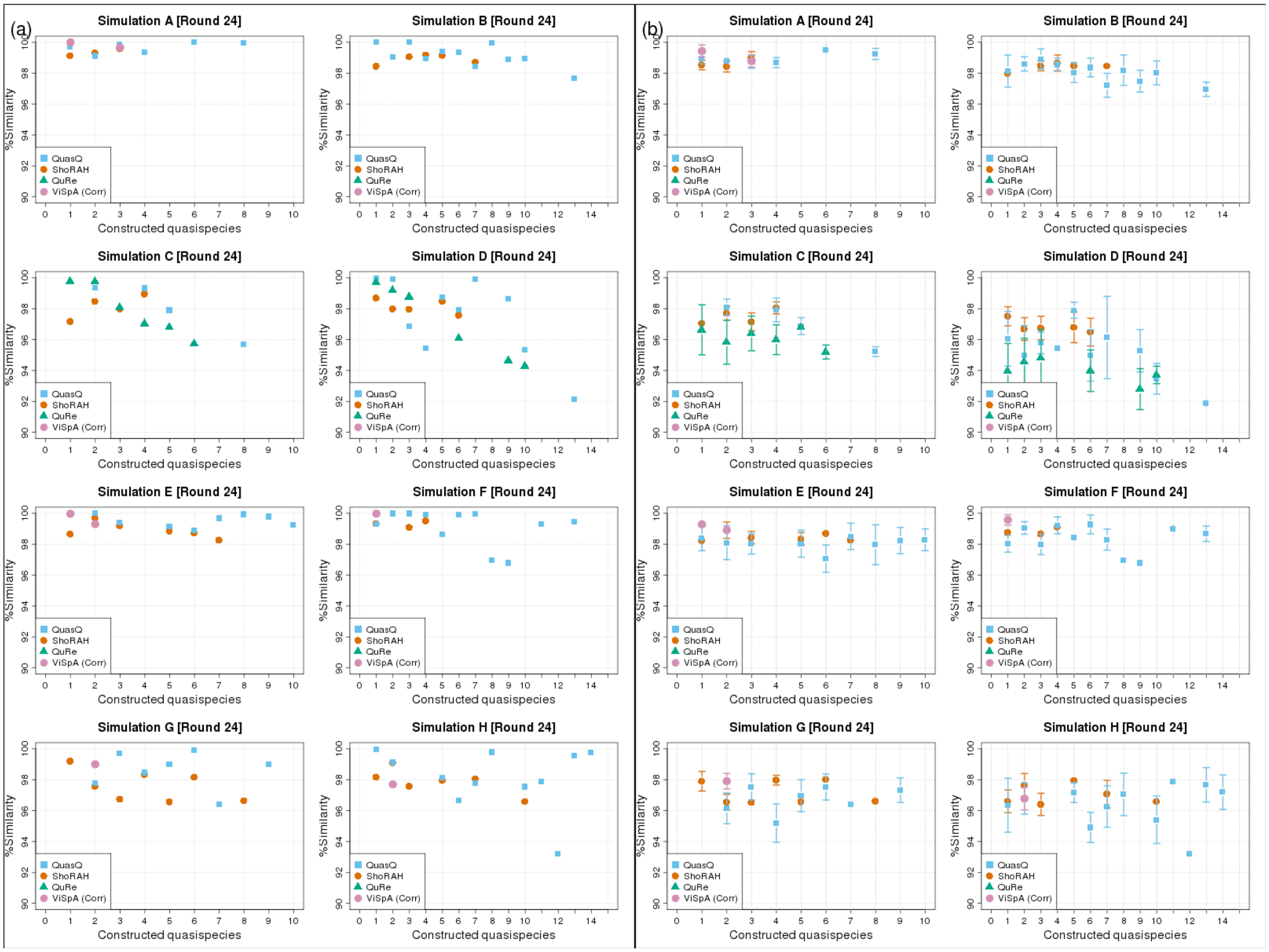

Supplementary Figure 28. (a) Degree of similarity between the best reconstructed haplotype sequences from QuasQ (blue squares), ShoRAH (red diamonds), QuRe (green triangles) and ViSpA with ShoRAH corrected reads (pink circle) with the actual simulated sequences. Here we identified the most similar reconstructed sequence to the simulated variants. (b) This shows the mean and standard deviation of the similarity for the reconstructed sequences that are mapped to a particular simulated variant. The above figures are based on the data from the 24th round of the simulations.

Supplementary Figure 29.

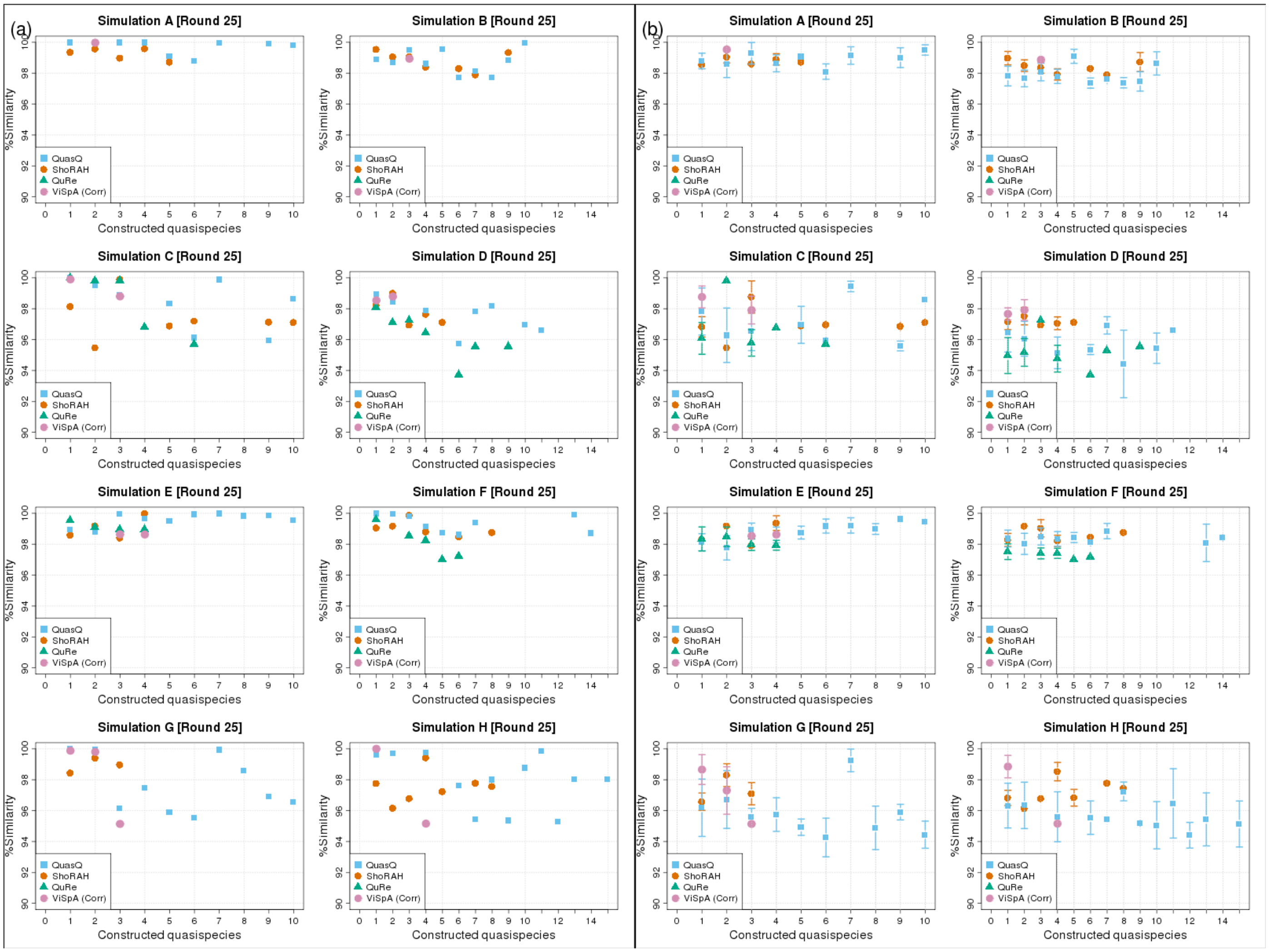

Supplementary Figure 29. (a) Degree of similarity between the best reconstructed haplotype sequences from QuasQ (blue squares), ShoRAH (red diamonds), QuRe (green triangles) and ViSpA with ShoRAH corrected reads (pink circle) with the actual simulated sequences. Here we identified the most similar reconstructed sequence to the simulated variants. (b) This shows the mean and standard deviation of the similarity for the reconstructed sequences that are mapped to a particular simulated variant. The above figures are based on the data from the 25th round of the simulations.

Supplementary Figure 30.

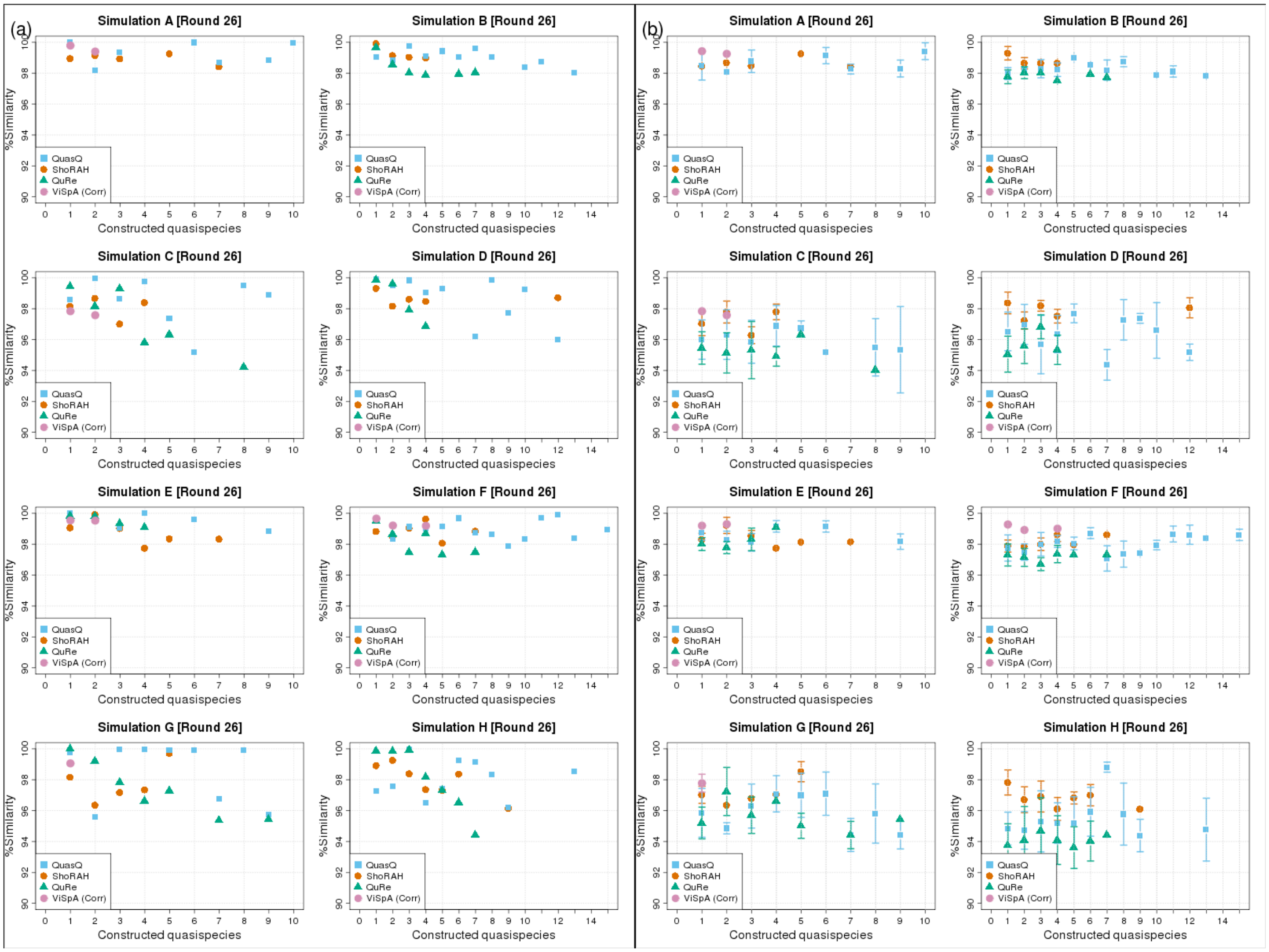

Supplementary Figure 30. (a) Degree of similarity between the best reconstructed haplotype sequences from QuasQ (blue squares), ShoRAH (red diamonds), QuRe (green triangles) and ViSpA with ShoRAH corrected reads (pink circle) with the actual simulated sequences. Here we identified the most similar reconstructed sequence to the simulated variants. (b) This shows the mean and standard deviation of the similarity for the reconstructed sequences that are mapped to a particular simulated variant. The above figures are based on the data from the 26th round of the simulations.

Supplementary Figure 31.

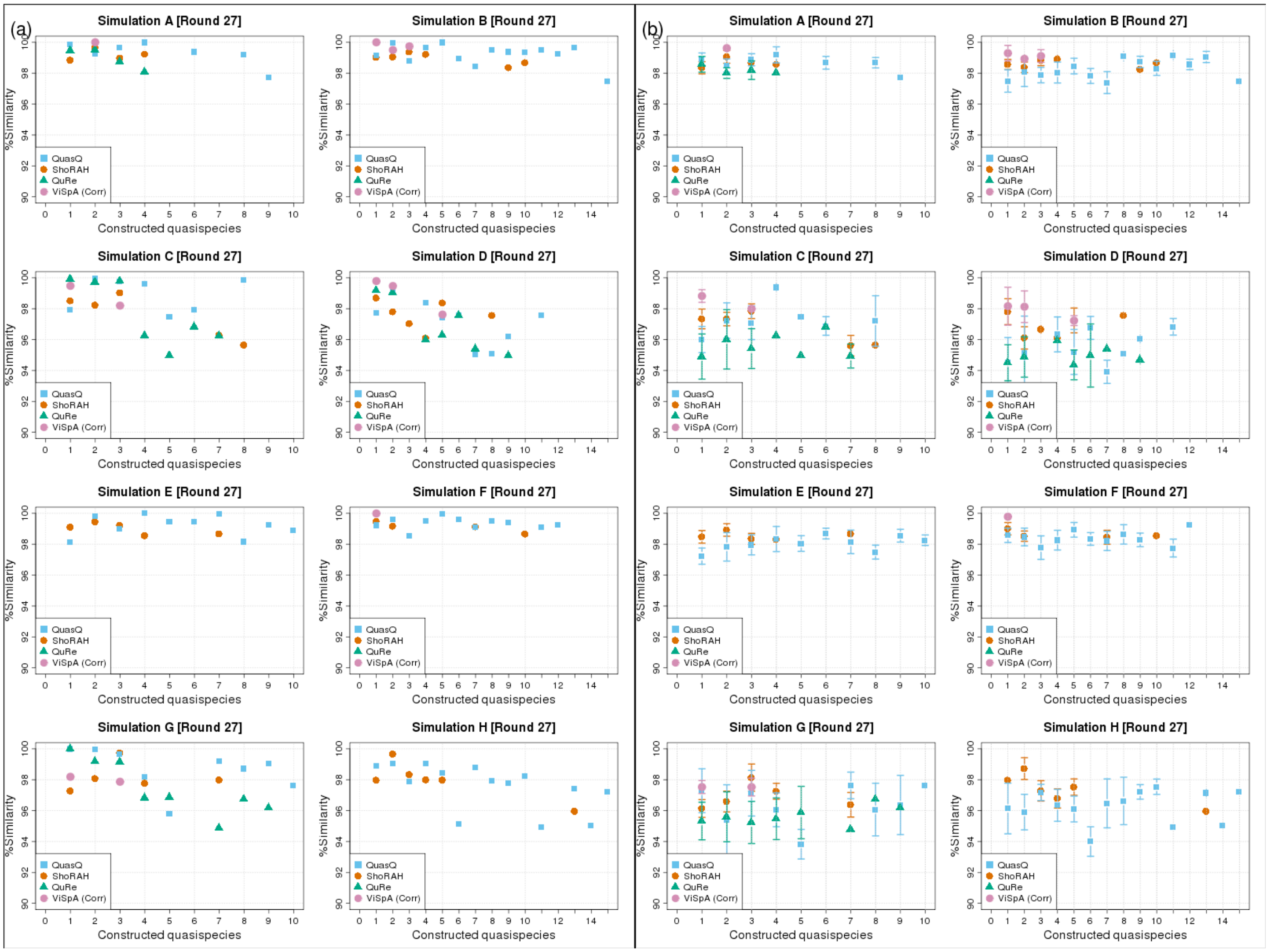

Supplementary Figure 31. (a) Degree of similarity between the best reconstructed haplotype sequences from QuasQ (blue squares), ShoRAH (red diamonds), QuRe (green triangles) and ViSpA with ShoRAH corrected reads (pink circle) with the actual simulated sequences. Here we identified the most similar reconstructed sequence to the simulated variants. (b) This shows the mean and standard deviation of the similarity for the reconstructed sequences that are mapped to a particular simulated variant. The above figures are based on the data from the 27th round of the simulations.

Supplementary Figure 32.

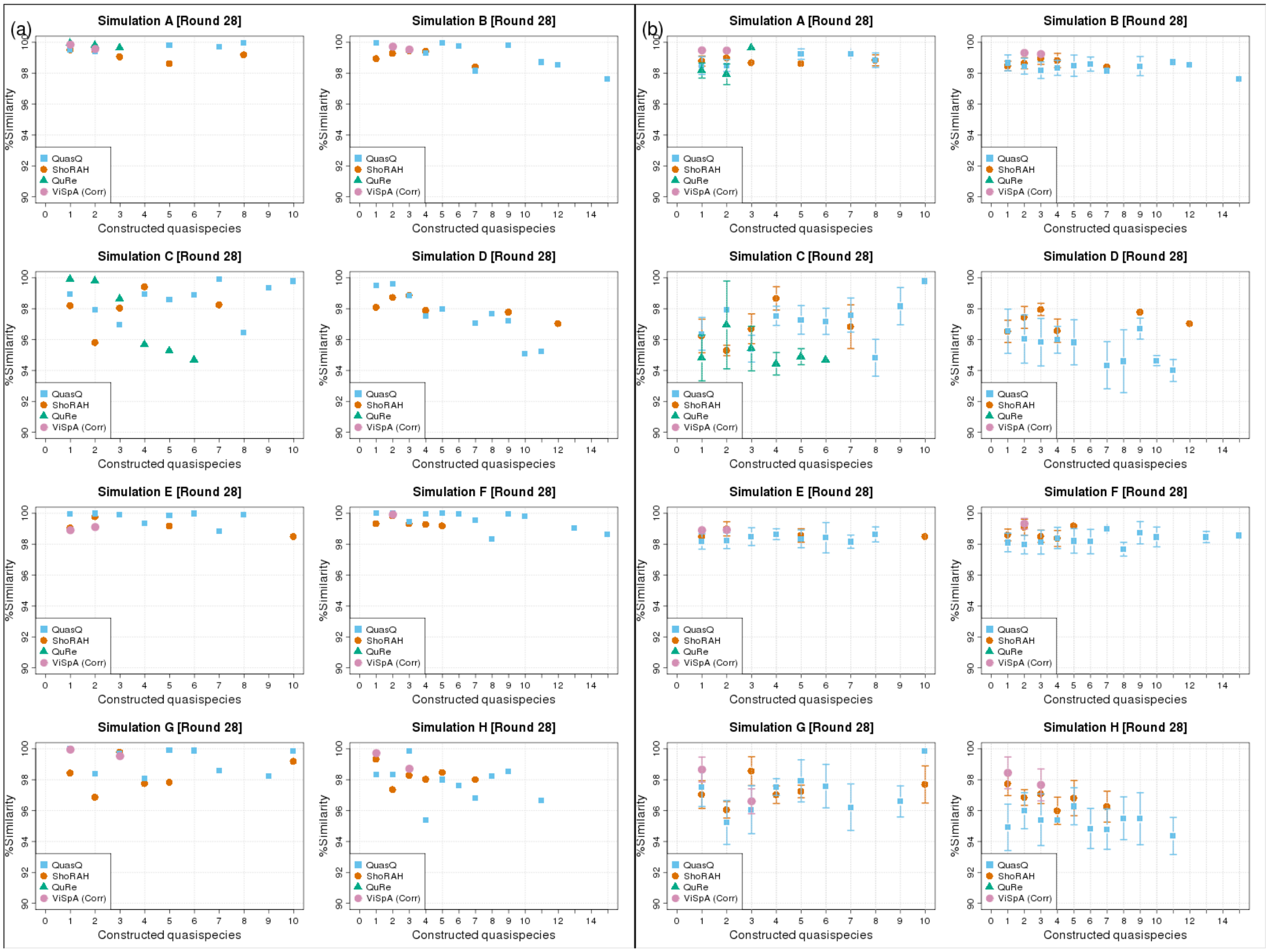

Supplementary Figure 32. (a) Degree of similarity between the best reconstructed haplotype sequences from QuasQ (blue squares), ShoRAH (red diamonds), QuRe (green triangles) and ViSpA with ShoRAH corrected reads (pink circle) with the actual simulated sequences. Here we identified the most similar reconstructed sequence to the simulated variants. (b) This shows the mean and standard deviation of the similarity for the reconstructed sequences that are mapped to a particular simulated variant. The above figures are based on the data from the 28th round of the simulations.

Supplementary Figure 33.

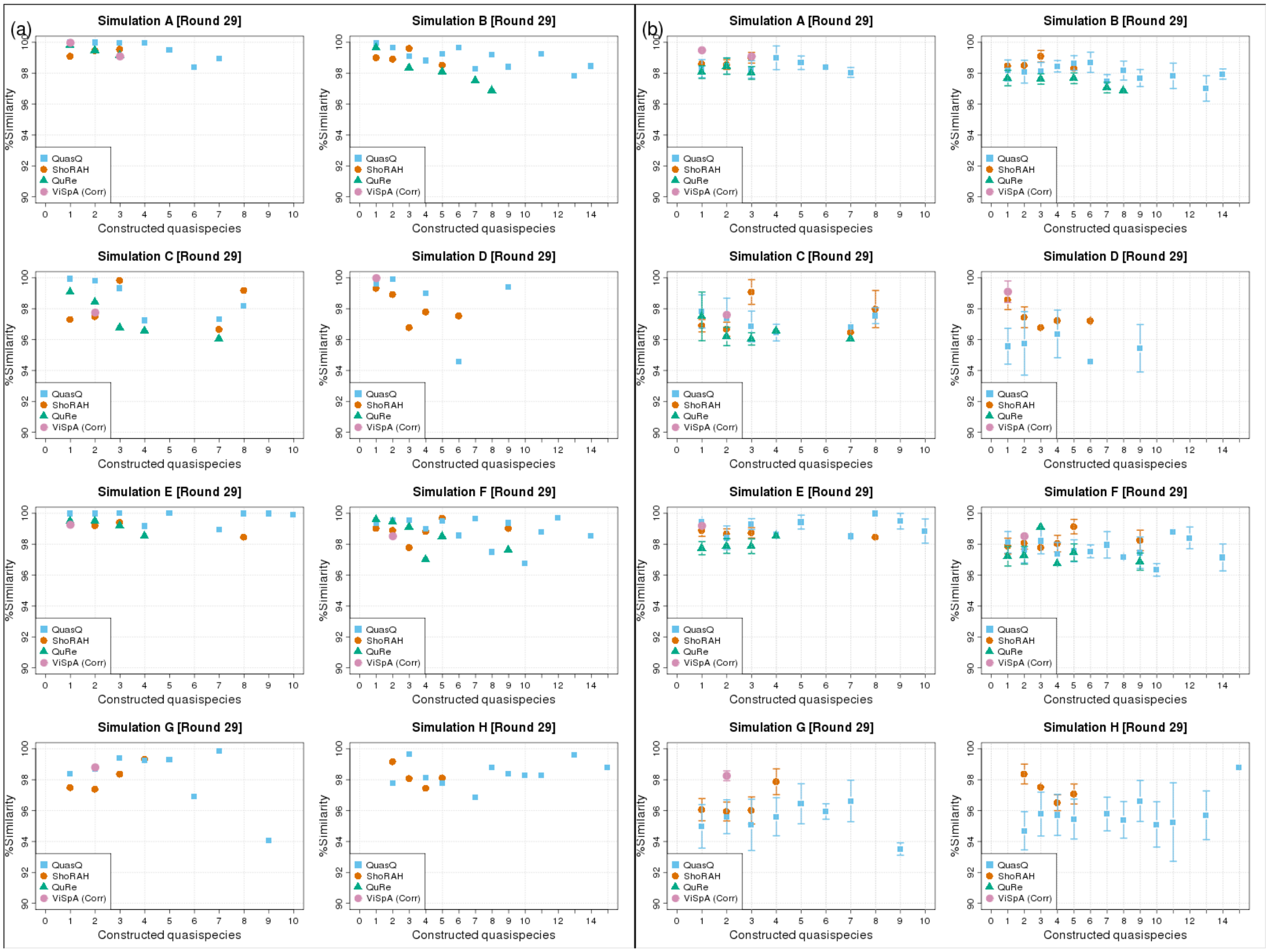

Supplementary Figure 33. (a) Degree of similarity between the best reconstructed haplotype sequences from QuasQ (blue squares), ShoRAH (red diamonds), QuRe (green triangles) and ViSpA with ShoRAH corrected reads (pink circle) with the actual simulated sequences. Here we identified the most similar reconstructed sequence to the simulated variants. (b) This shows the mean and standard deviation of the similarity for the reconstructed sequences that are mapped to a particular simulated variant. The above figures are based on the data from the 29th round of the simulations.

Supplementary Figure 34.

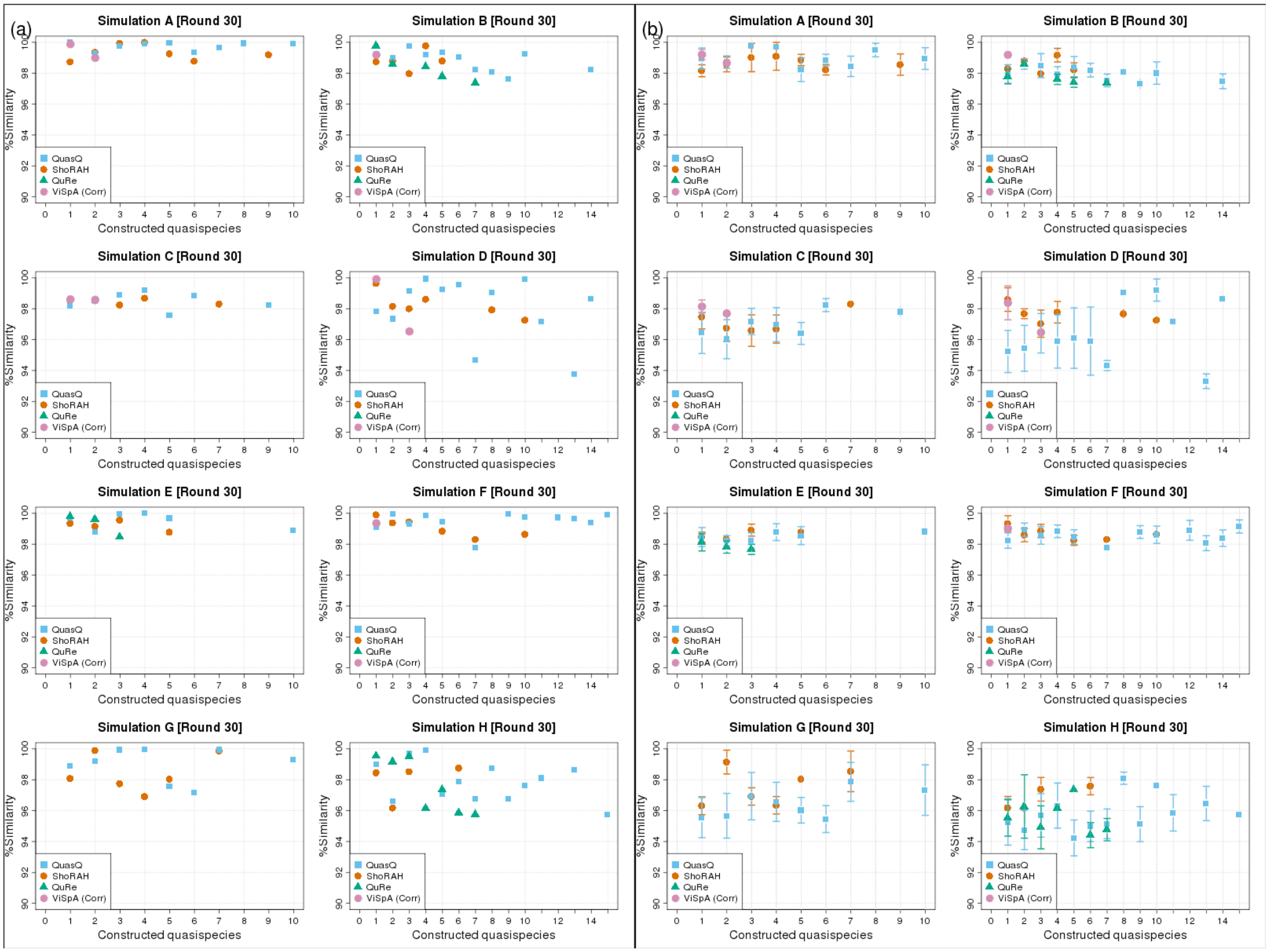

Supplementary Figure 34. (a) Degree of similarity between the best reconstructed haplotype sequences from QuasQ (blue squares), ShoRAH (red diamonds), QuRe (green triangles) and ViSpA with ShoRAH corrected reads (pink circle) with the actual simulated sequences. Here we identified the most similar reconstructed sequence to the simulated variants. (b) This shows the mean and standard deviation of the similarity for the reconstructed sequences that are mapped to a particular simulated variant. The above figures are based on the data from the 30th round of the simulations.

Supplementary Figure 35.

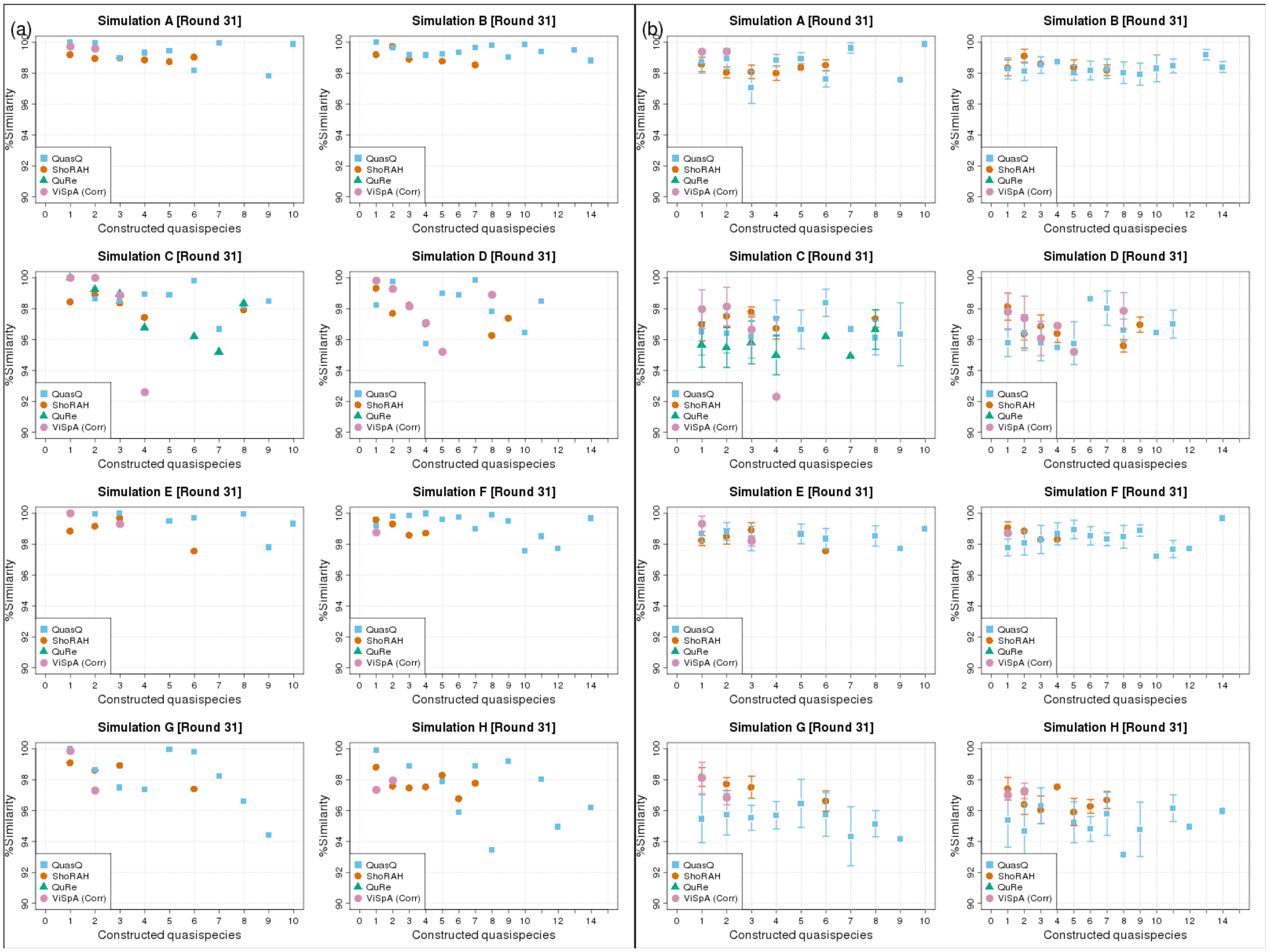

Supplementary Figure 35. (a) Degree of similarity between the best reconstructed haplotype sequences from QuasQ (blue squares), ShoRAH (red diamonds), QuRe (green triangles) and ViSpA with ShoRAH corrected reads (pink circle) with the actual simulated sequences. Here we identified the most similar reconstructed sequence to the simulated variants. (b) This shows the mean and standard deviation of the similarity for the reconstructed sequences that are mapped to a particular simulated variant. The above figures are based on the data from the 31st round of the simulations.

Supplementary Figure 36.

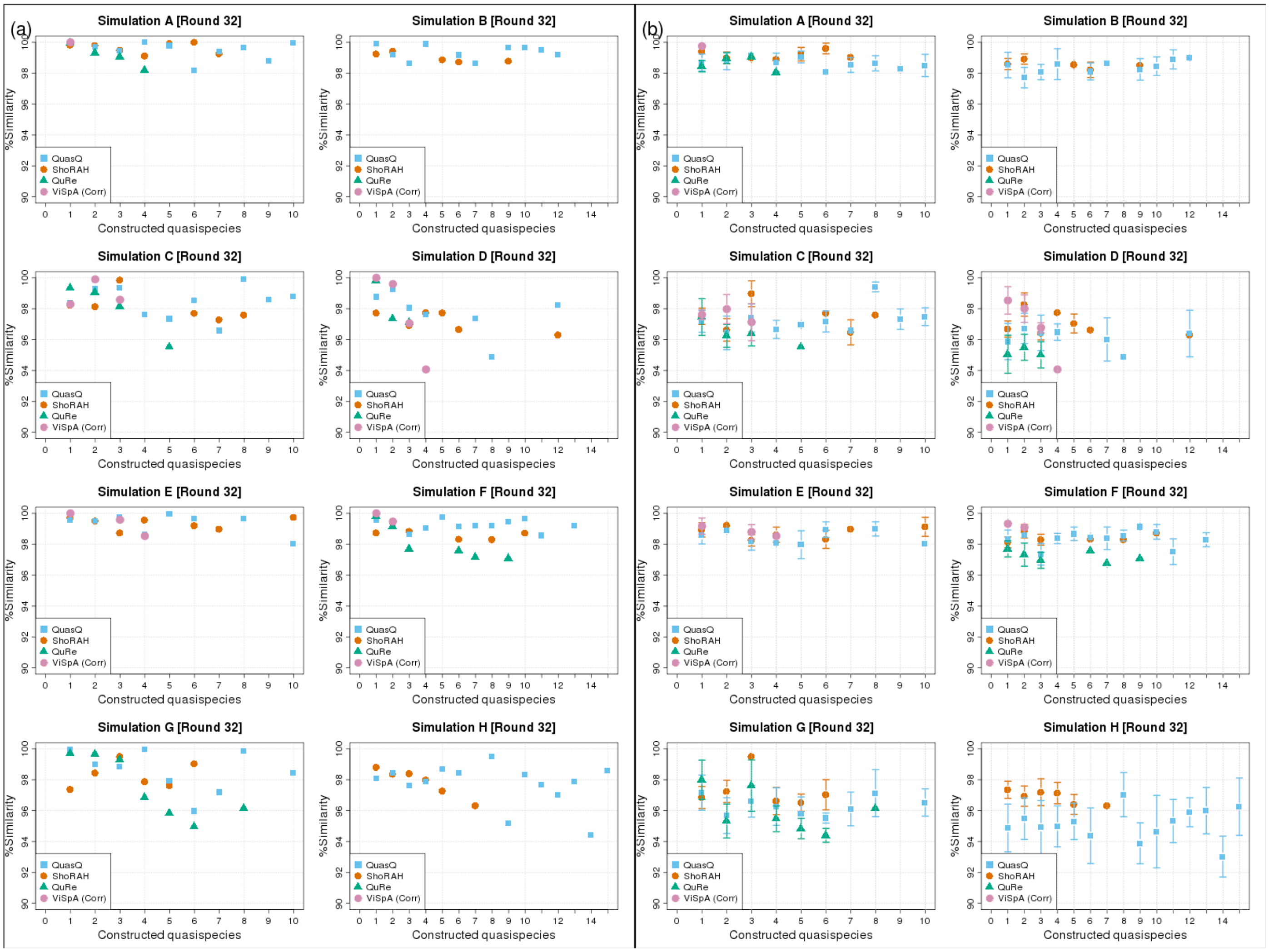

Supplementary Figure 36. (a) Degree of similarity between the best reconstructed haplotype sequences from QuasQ (blue squares), ShoRAH (red diamonds), QuRe (green triangles) and ViSpA with ShoRAH corrected reads (pink circle) with the actual simulated sequences. Here we identified the most similar reconstructed sequence to the simulated variants. (b) This shows the mean and standard deviation of the similarity for the reconstructed sequences that are mapped to a particular simulated variant. The above figures are based on the data from the 32nd round of the simulations.

Supplementary Figure 37.

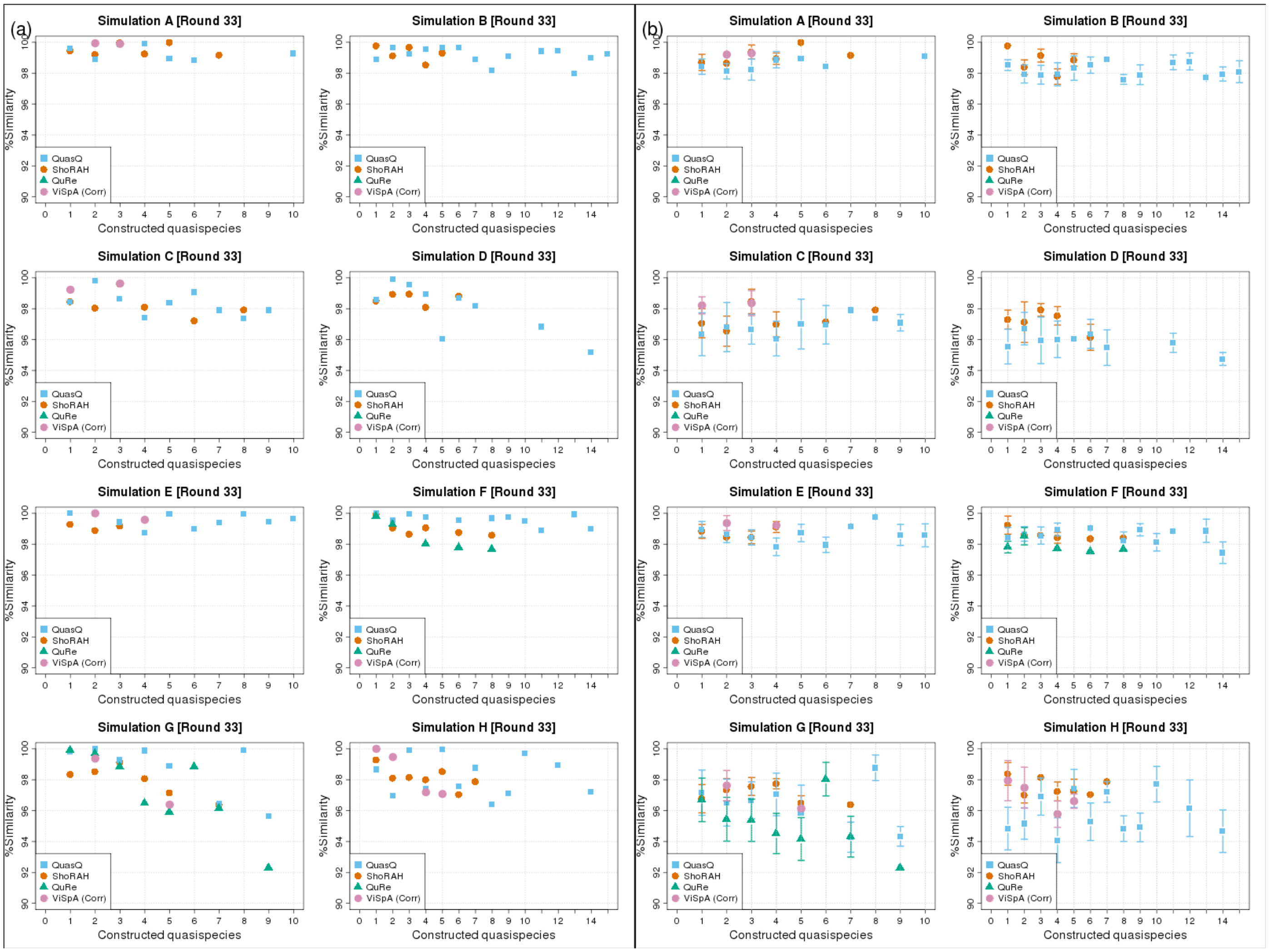

Supplementary Figure 37. (a) Degree of similarity between the best reconstructed haplotype sequences from QuasQ (blue squares), ShoRAH (red diamonds), QuRe (green triangles) and ViSpA with ShoRAH corrected reads (pink circle) with the actual simulated sequences. Here we identified the most similar reconstructed sequence to the simulated variants. (b) This shows the mean and standard deviation of the similarity for the reconstructed sequences that are mapped to a particular simulated variant. The above figures are based on the data from the 33rd round of the simulations.

Supplementary Figure 38.

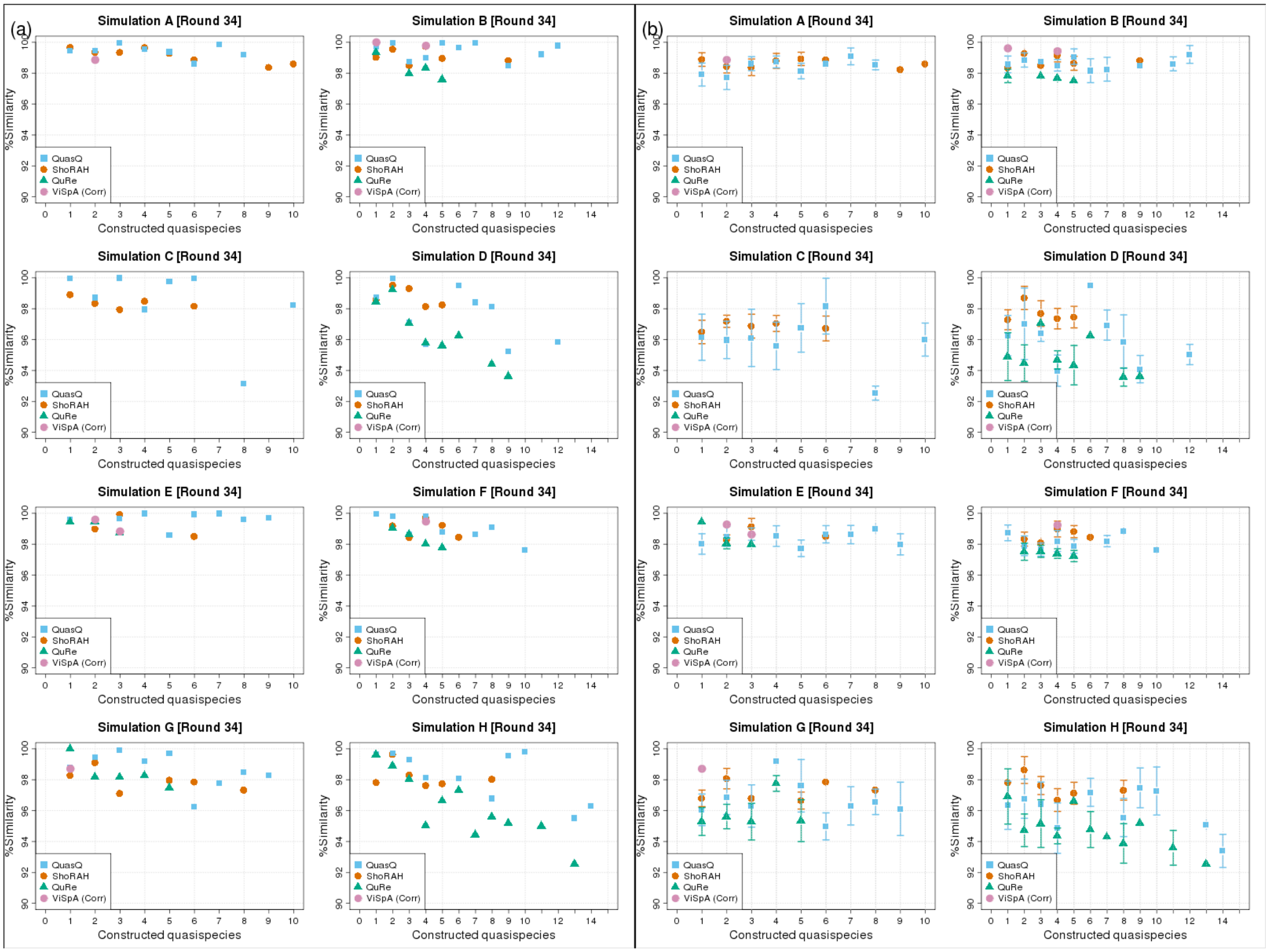

Supplementary Figure 38. (a) Degree of similarity between the best reconstructed haplotype sequences from QuasQ (blue squares), ShoRAH (red diamonds), QuRe (green triangles) and ViSpA with ShoRAH corrected reads (pink circle) with the actual simulated sequences. Here we identified the most similar reconstructed sequence to the simulated variants. (b) This shows the mean and standard deviation of the similarity for the reconstructed sequences that are mapped to a particular simulated variant. The above figures are based on the data from the 34th round of the simulations.

Supplementary Figure 39.

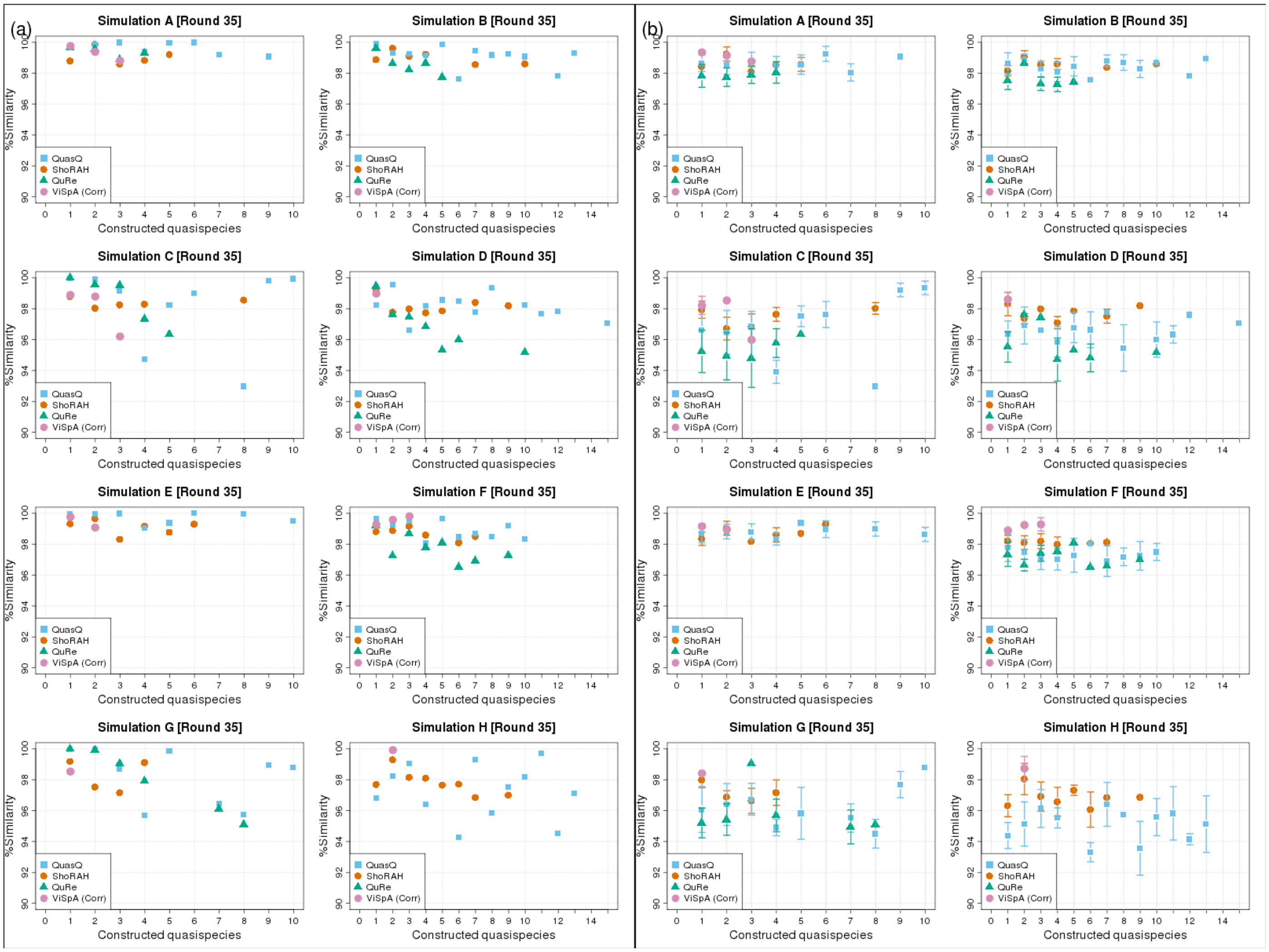

Supplementary Figure 39. (a) Degree of similarity between the best reconstructed haplotype sequences from QuasQ (blue squares), ShoRAH (red diamonds), QuRe (green triangles) and ViSpA with ShoRAH corrected reads (pink circle) with the actual simulated sequences. Here we identified the most similar reconstructed sequence to the simulated variants. (b) This shows the mean and standard deviation of the similarity for the reconstructed sequences that are mapped to a particular simulated variant. The above figures are based on the data from the 35th round of the simulations.

Supplementary Figure 40.

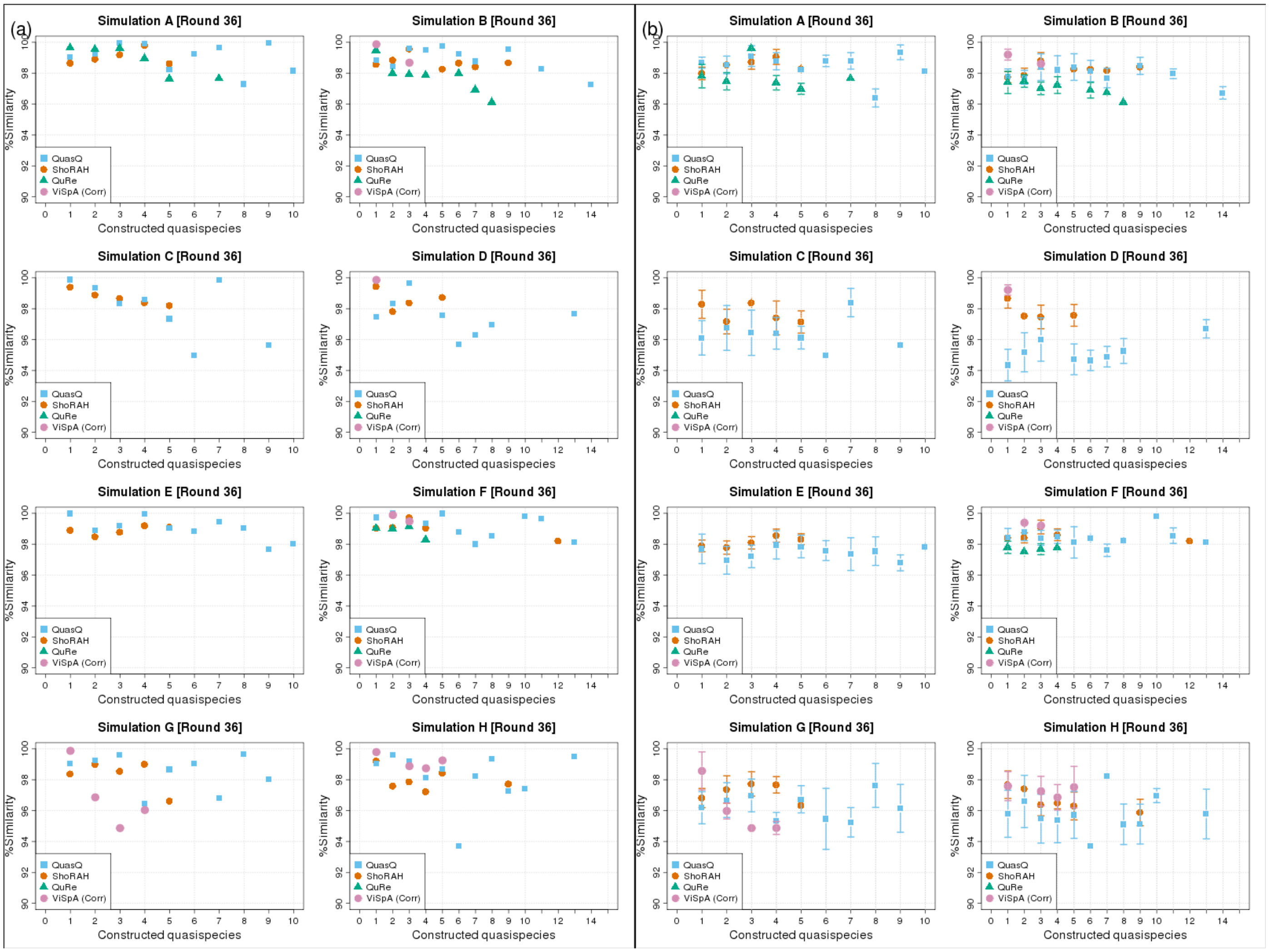

Supplementary Figure 40. (a) Degree of similarity between the best reconstructed haplotype sequences from QuasQ (blue squares), ShoRAH (red diamonds), QuRe (green triangles) and ViSpA with ShoRAH corrected reads (pink circle) with the actual simulated sequences. Here we identified the most similar reconstructed sequence to the simulated variants. (b) This shows the mean and standard deviation of the similarity for the reconstructed sequences that are mapped to a particular simulated variant. The above figures are based on the data from the 36th round of the simulations.

Supplementary Figure 41.

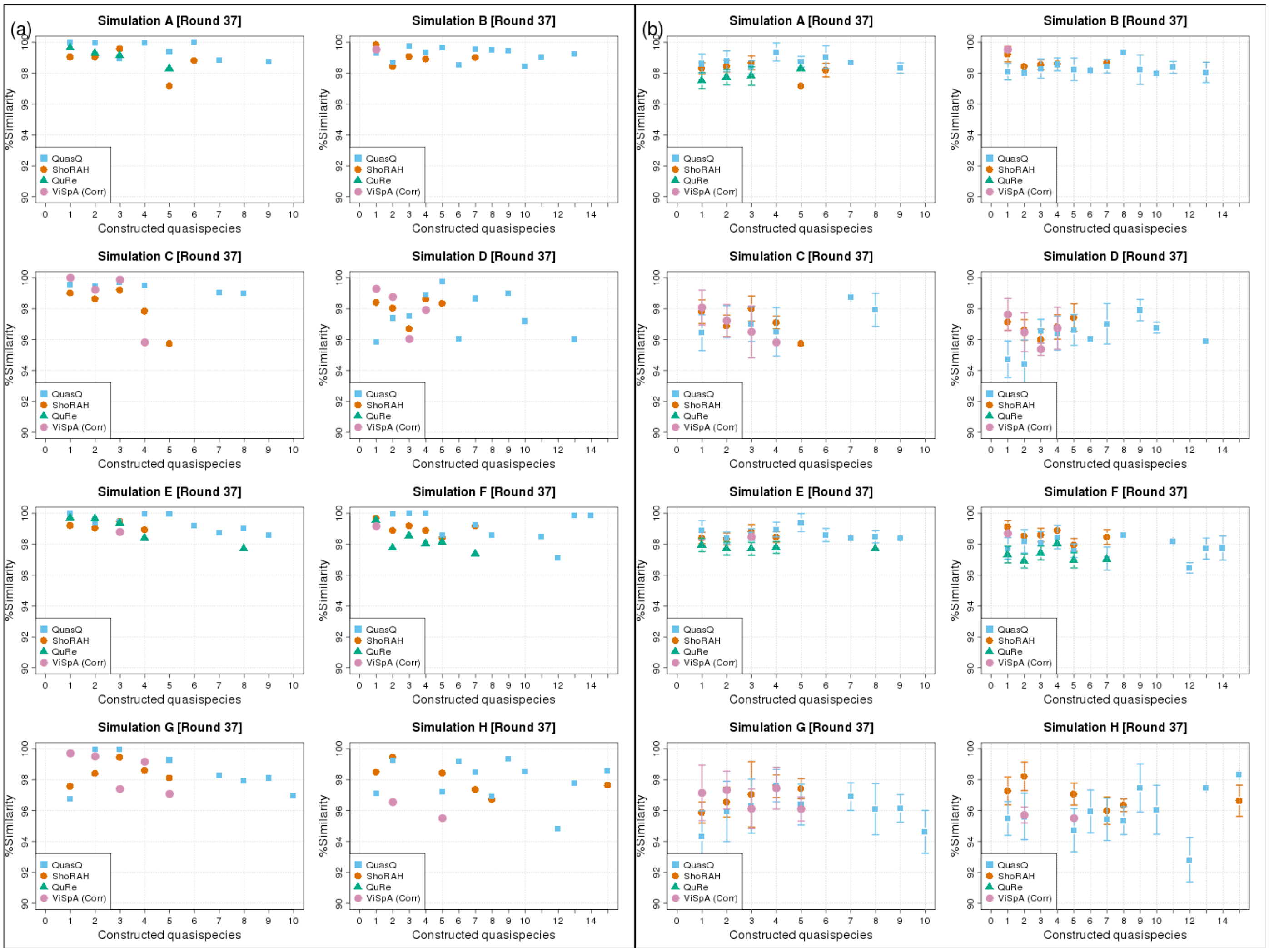

Supplementary Figure 41. (a) Degree of similarity between the best reconstructed haplotype sequences from QuasQ (blue squares), ShoRAH (red diamonds), QuRe (green triangles) and ViSpA with ShoRAH corrected reads (pink circle) with the actual simulated sequences. Here we identified the most similar reconstructed sequence to the simulated variants. (b) This shows the mean and standard deviation of the similarity for the reconstructed sequences that are mapped to a particular simulated variant. The above figures are based on the data from the 37th round of the simulations.

Supplementary Figure 42.

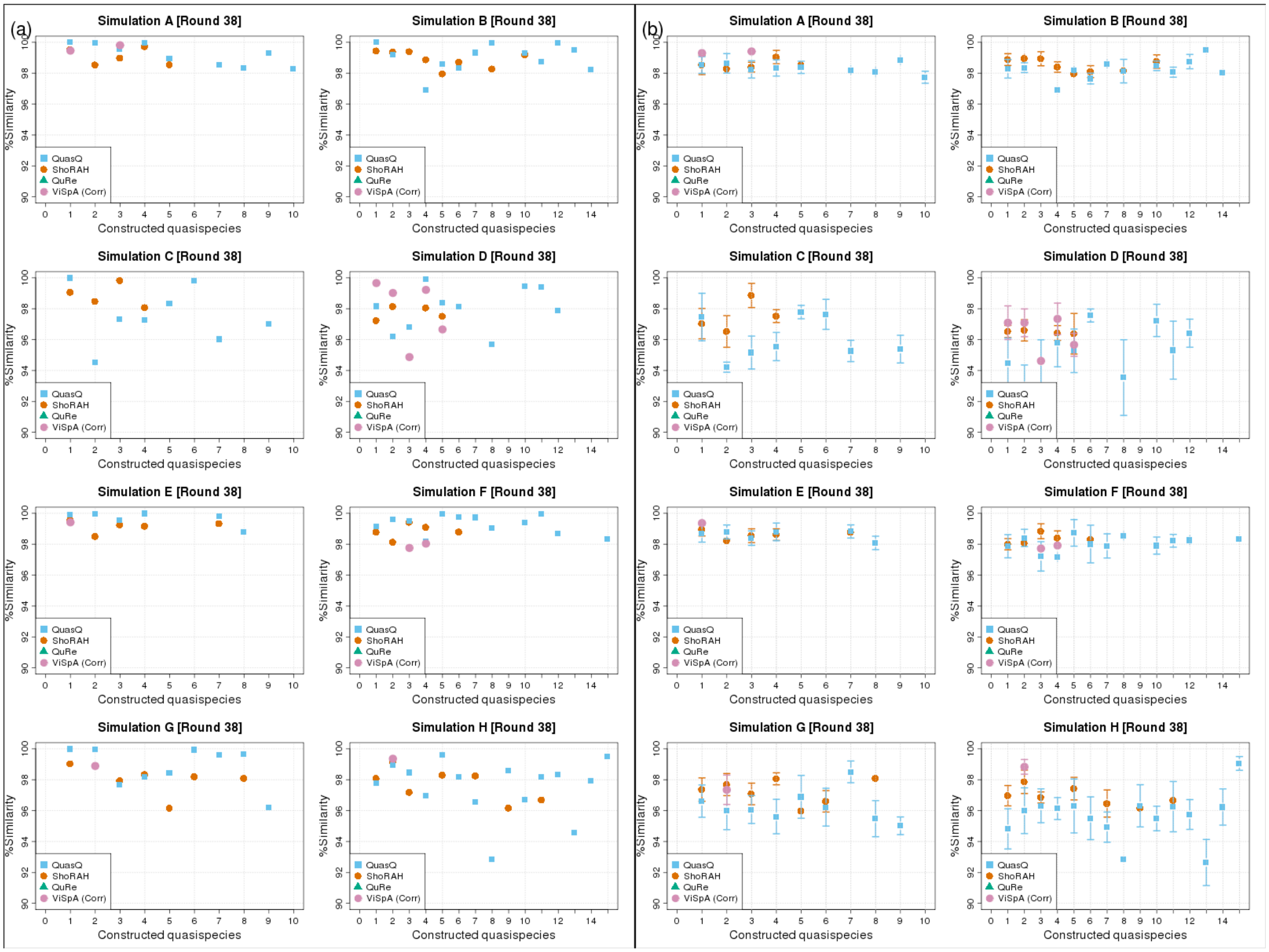

Supplementary Figure 42. (a) Degree of similarity between the best reconstructed haplotype sequences from QuasQ (blue squares), ShoRAH (red diamonds), QuRe (green triangles) and ViSpA with ShoRAH corrected reads (pink circle) with the actual simulated sequences. Here we identified the most similar reconstructed sequence to the simulated variants. (b) This shows the mean and standard deviation of the similarity for the reconstructed sequences that are mapped to a particular simulated variant. The above figures are based on the data from the 38th round of the simulations.

Supplementary Figure 43.

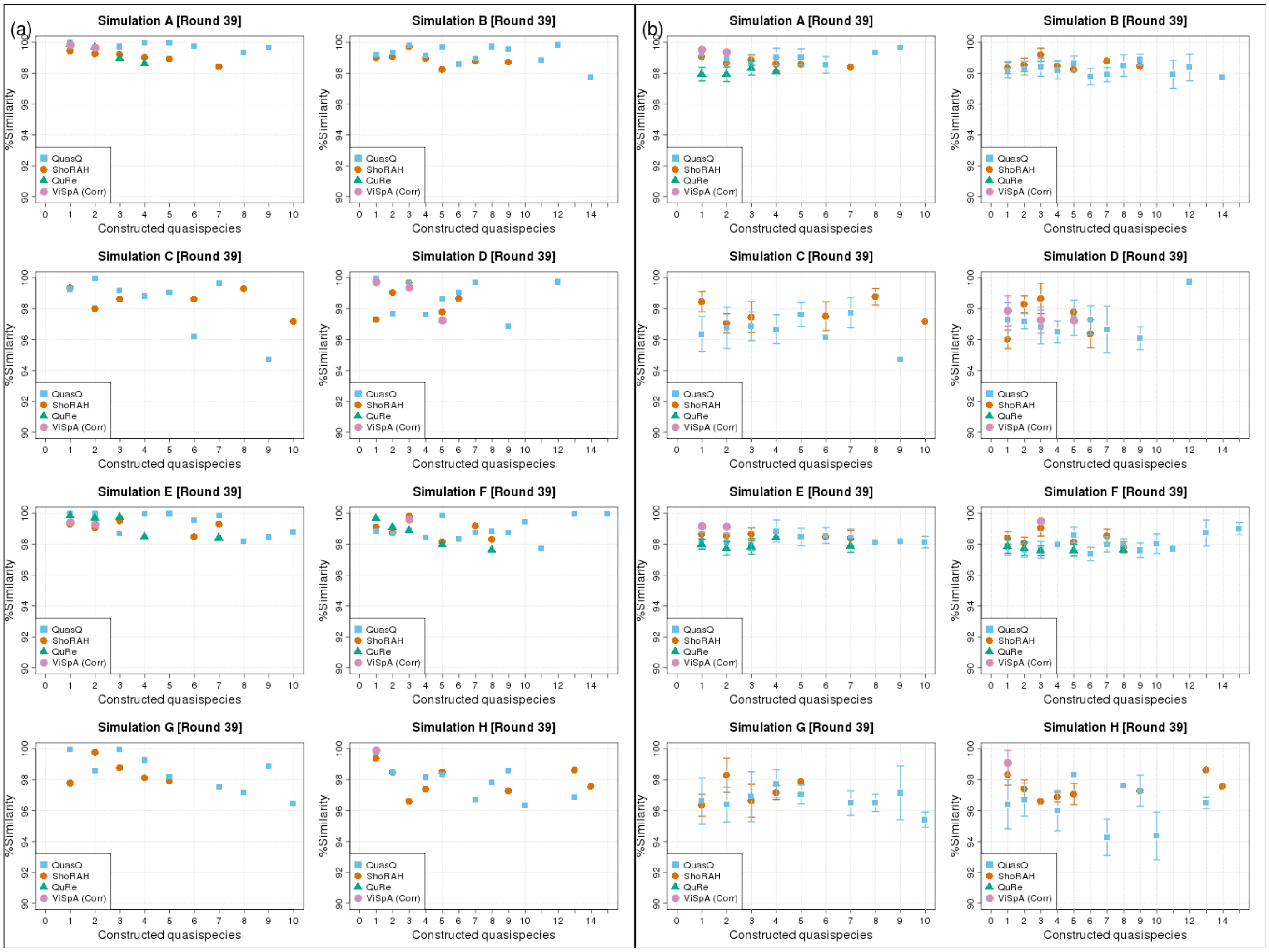

Supplementary Figure 43. (a) Degree of similarity between the best reconstructed haplotype sequences from QuasQ (blue squares), ShoRAH (red diamonds), QuRe (green triangles) and ViSpA with ShoRAH corrected reads (pink circle) with the actual simulated sequences. Here we identified the most similar reconstructed sequence to the simulated variants. (b) This shows the mean and standard deviation of the similarity for the reconstructed sequences that are mapped to a particular simulated variant. The above figures are based on the data from the 39th round of the simulations.

Supplementary Figure 44.

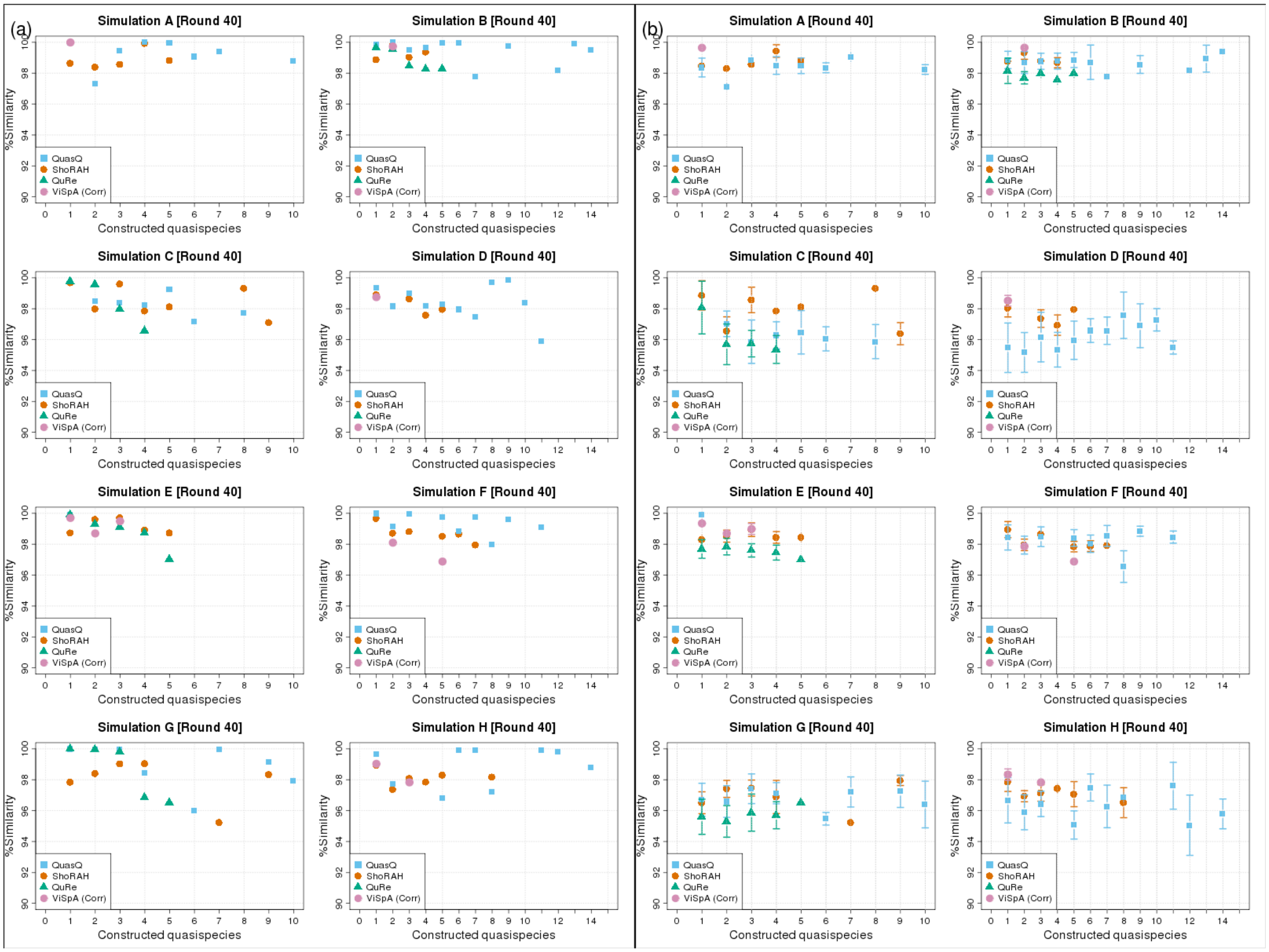

Supplementary Figure 44. (a) Degree of similarity between the best reconstructed haplotype sequences from QuasQ (blue squares), ShoRAH (red diamonds), QuRe (green triangles) and ViSpA with ShoRAH corrected reads (pink circle) with the actual simulated sequences. Here we identified the most similar reconstructed sequence to the simulated variants. (b) This shows the mean and standard deviation of the similarity for the reconstructed sequences that are mapped to a particular simulated variant. The above figures are based on the data from the 40th round of the simulations.

Supplementary Figure 45.

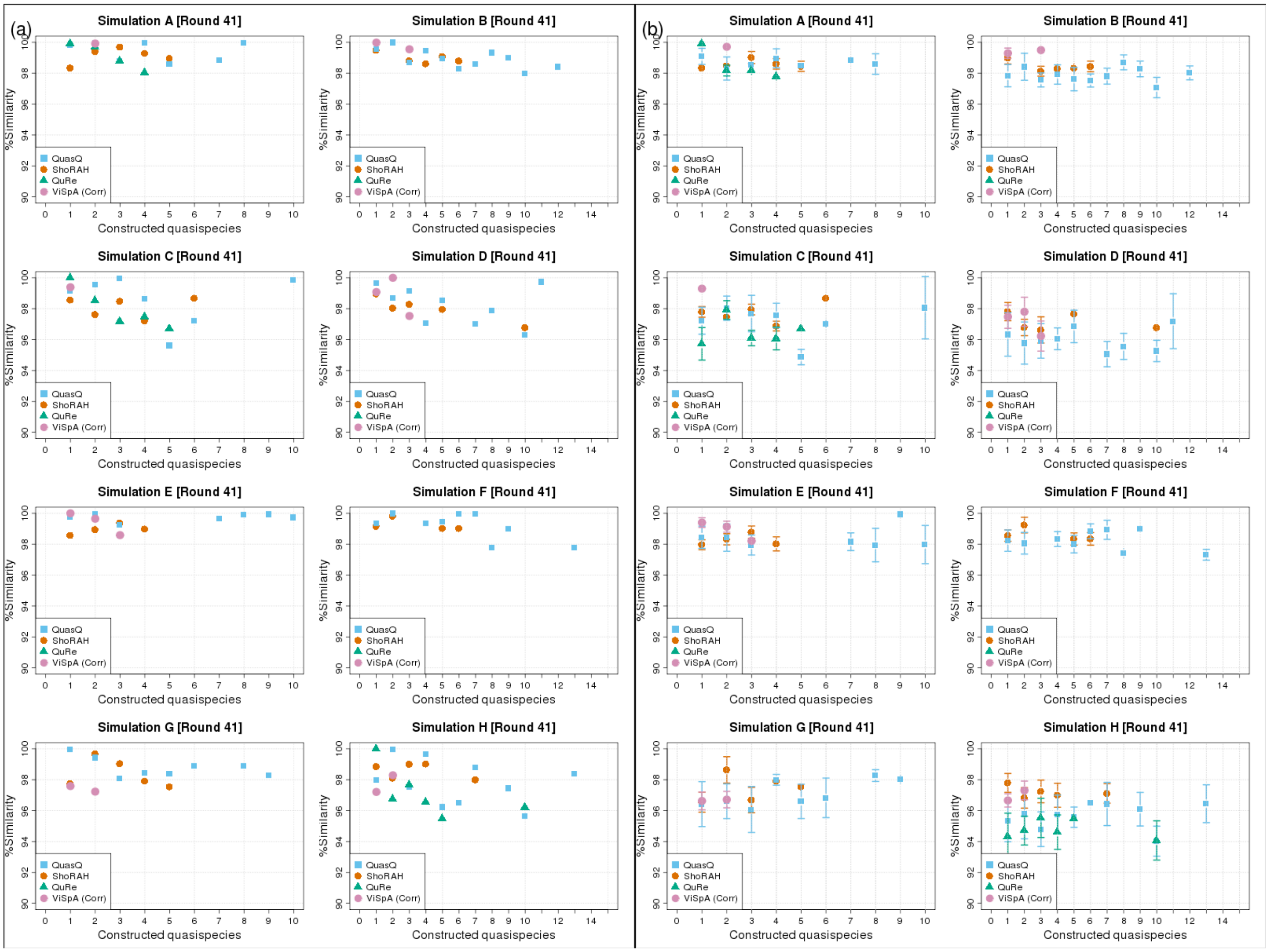

Supplementary Figure 45. (a) Degree of similarity between the best reconstructed haplotype sequences from QuasQ (blue squares), ShoRAH (red diamonds), QuRe (green triangles) and ViSpA with ShoRAH corrected reads (pink circle) with the actual simulated sequences. Here we identified the most similar reconstructed sequence to the simulated variants. (b) This shows the mean and standard deviation of the similarity for the reconstructed sequences that are mapped to a particular simulated variant. The above figures are based on the data from the 41st round of the simulations.

Supplementary Figure 46.

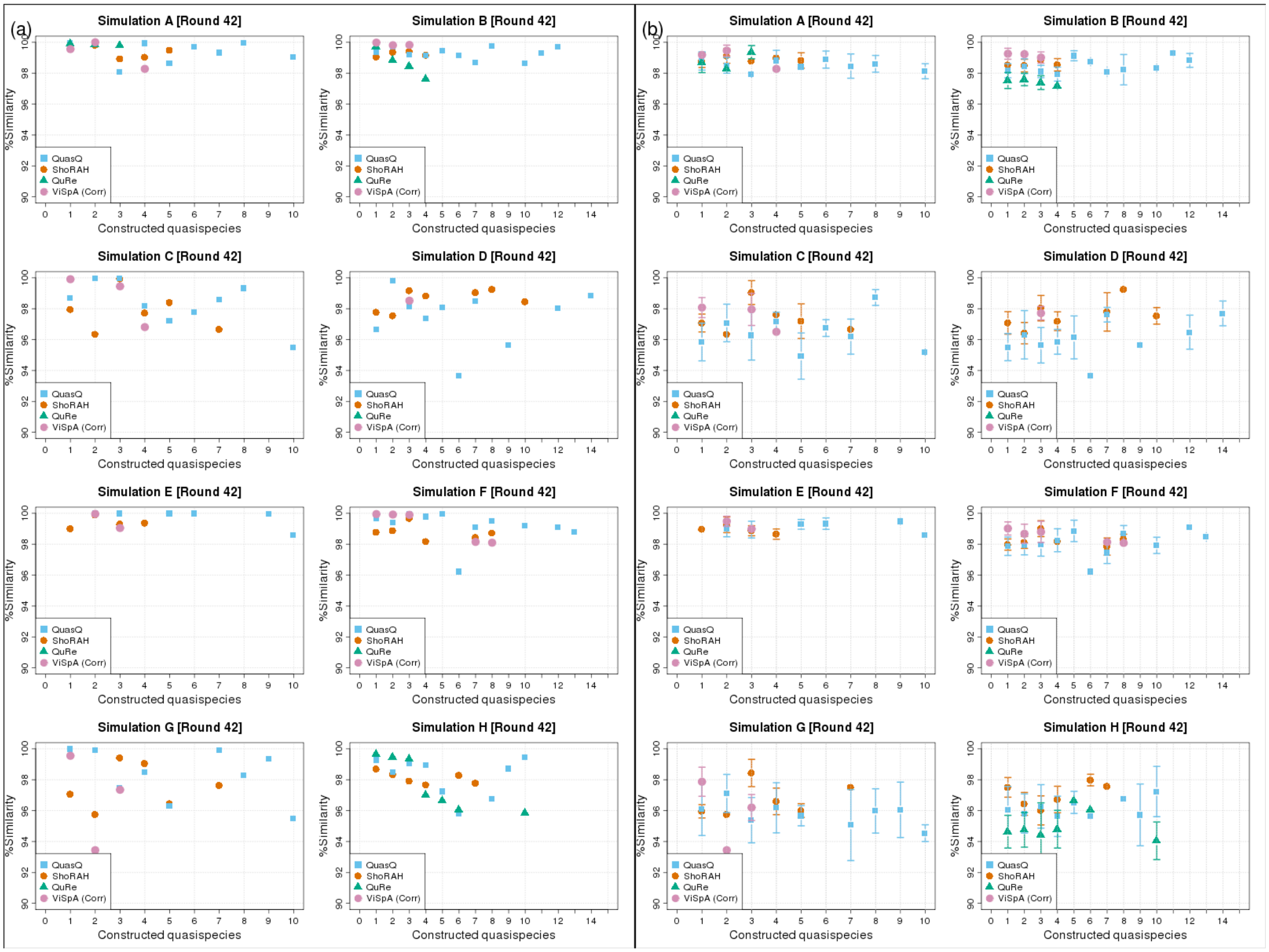

Supplementary Figure 46. (a) Degree of similarity between the best reconstructed haplotype sequences from QuasQ (blue squares), ShoRAH (red diamonds), QuRe (green triangles) and ViSpA with ShoRAH corrected reads (pink circle) with the actual simulated sequences. Here we identified the most similar reconstructed sequence to the simulated variants. (b) This shows the mean and standard deviation of the similarity for the reconstructed sequences that are mapped to a particular simulated variant. The above figures are based on the data from the 42nd round of the simulations.

Supplementary Figure 47.

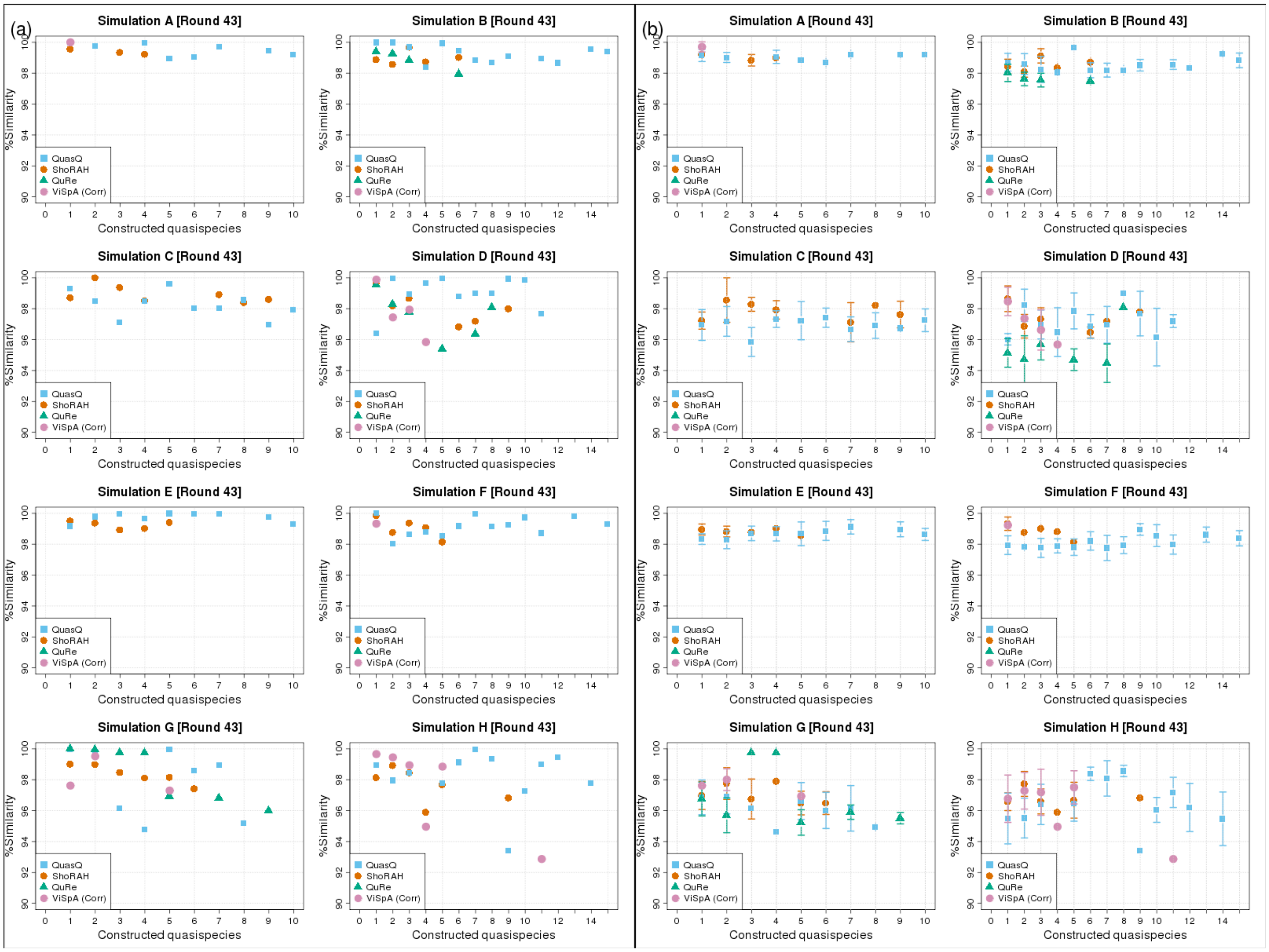

Supplementary Figure 47. (a) Degree of similarity between the best reconstructed haplotype sequences from QuasQ (blue squares), ShoRAH (red diamonds), QuRe (green triangles) and ViSpA with ShoRAH corrected reads (pink circle) with the actual simulated sequences. Here we identified the most similar reconstructed sequence to the simulated variants. (b) This shows the mean and standard deviation of the similarity for the reconstructed sequences that are mapped to a particular simulated variant. The above figures are based on the data from the 43rd round of the simulations.

Supplementary Figure 48.

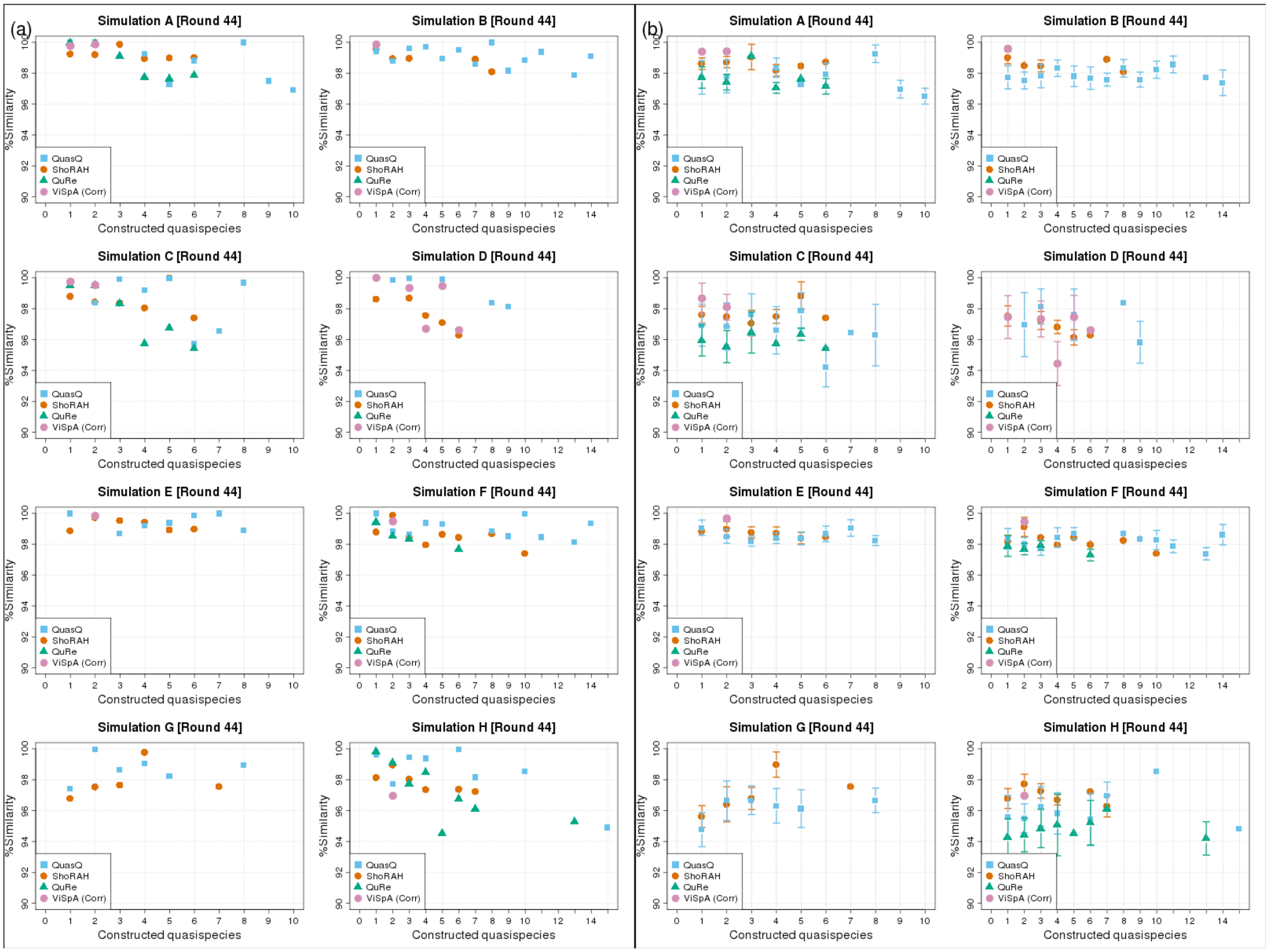

Supplementary Figure 48. (a) Degree of similarity between the best reconstructed haplotype sequences from QuasQ (blue squares), ShoRAH (red diamonds), QuRe (green triangles) and ViSpA with ShoRAH corrected reads (pink circle) with the actual simulated sequences. Here we identified the most similar reconstructed sequence to the simulated variants. (b) This shows the mean and standard deviation of the similarity for the reconstructed sequences that are mapped to a particular simulated variant. The above figures are based on the data from the 44th round of the simulations.

Supplementary Figure 49.

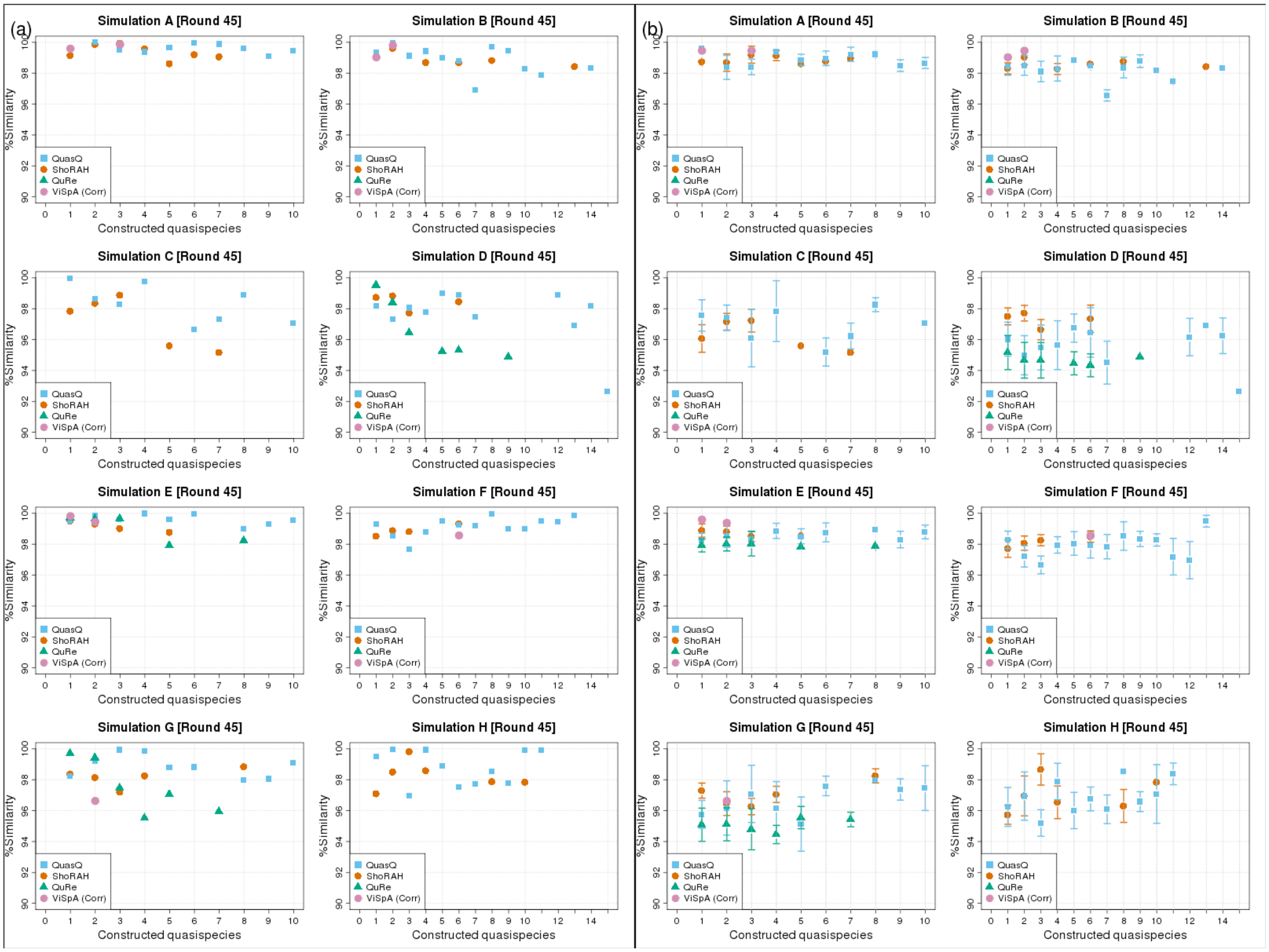

Supplementary Figure 49. (a) Degree of similarity between the best reconstructed haplotype sequences from QuasQ (blue squares), ShoRAH (red diamonds), QuRe (green triangles) and ViSpA with ShoRAH corrected reads (pink circle) with the actual simulated sequences. Here we identified the most similar reconstructed sequence to the simulated variants. (b) This shows the mean and standard deviation of the similarity for the reconstructed sequences that are mapped to a particular simulated variant. The above figures are based on the data from the 45th round of the simulations.

Supplementary Figure 50.

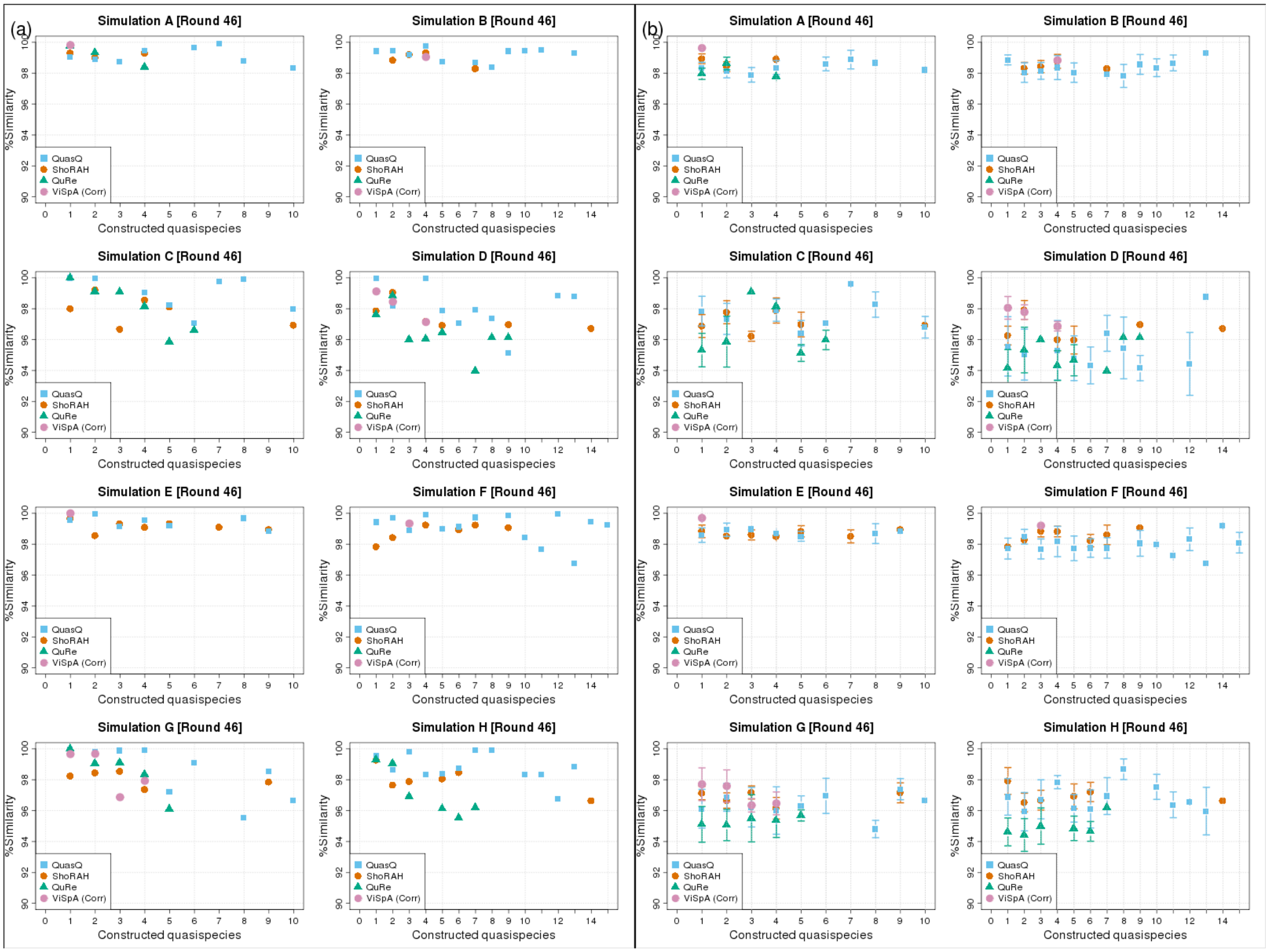

Supplementary Figure 50. (a) Degree of similarity between the best reconstructed haplotype sequences from QuasQ (blue squares), ShoRAH (red diamonds), QuRe (green triangles) and ViSpA with ShoRAH corrected reads (pink circle) with the actual simulated sequences. Here we identified the most similar reconstructed sequence to the simulated variants. (b) This shows the mean and standard deviation of the similarity for the reconstructed sequences that are mapped to a particular simulated variant. The above figures are based on the data from the 46th round of the simulations.

Supplementary Figure 51.

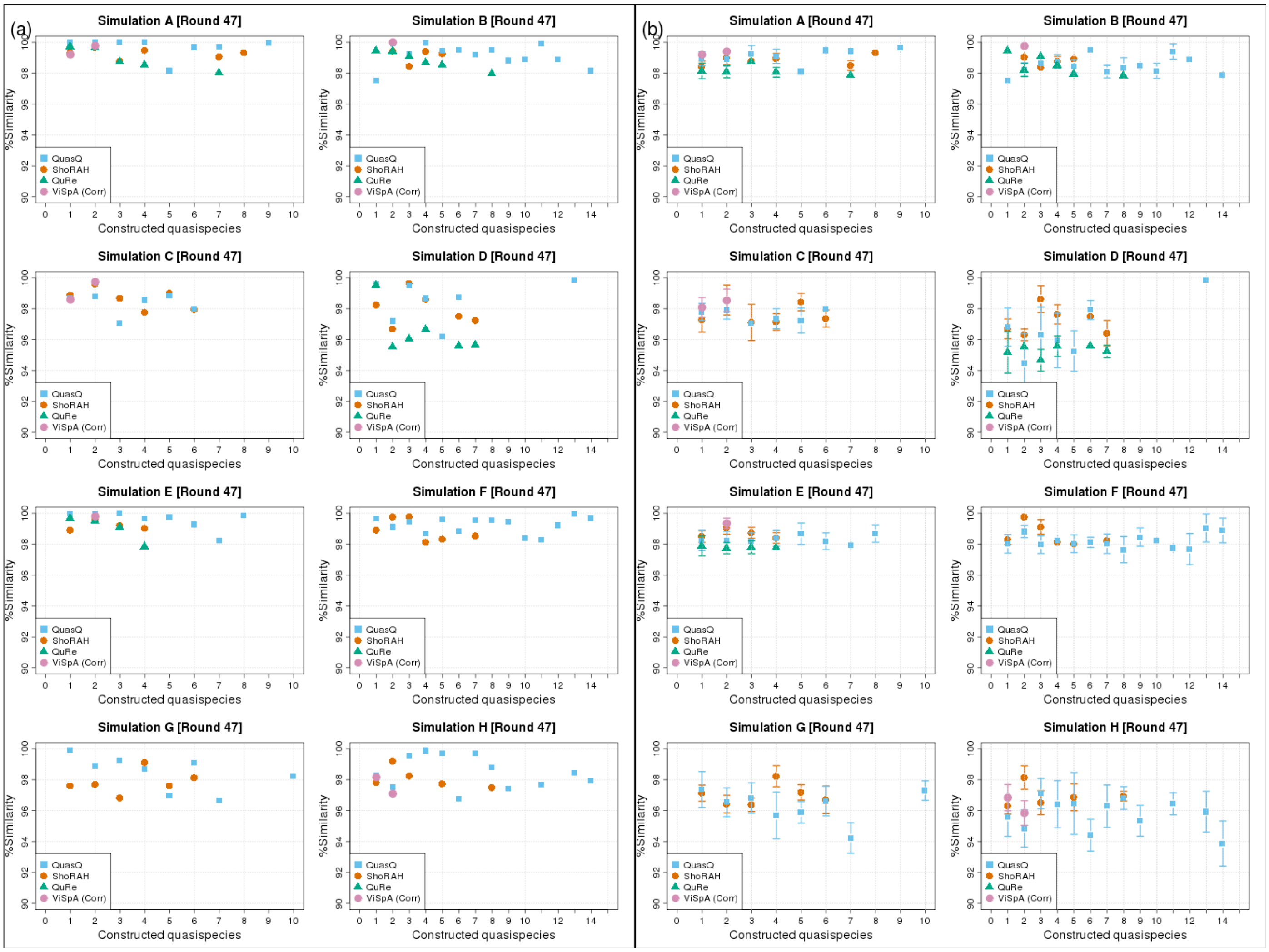

Supplementary Figure 51. (a) Degree of similarity between the best reconstructed haplotype sequences from QuasQ (blue squares), ShoRAH (red diamonds), QuRe (green triangles) and ViSpA with ShoRAH corrected reads (pink circle) with the actual simulated sequences. Here we identified the most similar reconstructed sequence to the simulated variants. (b) This shows the mean and standard deviation of the similarity for the reconstructed sequences that are mapped to a particular simulated variant. The above figures are based on the data from the 47th round of the simulations.

Supplementary Figure 52.

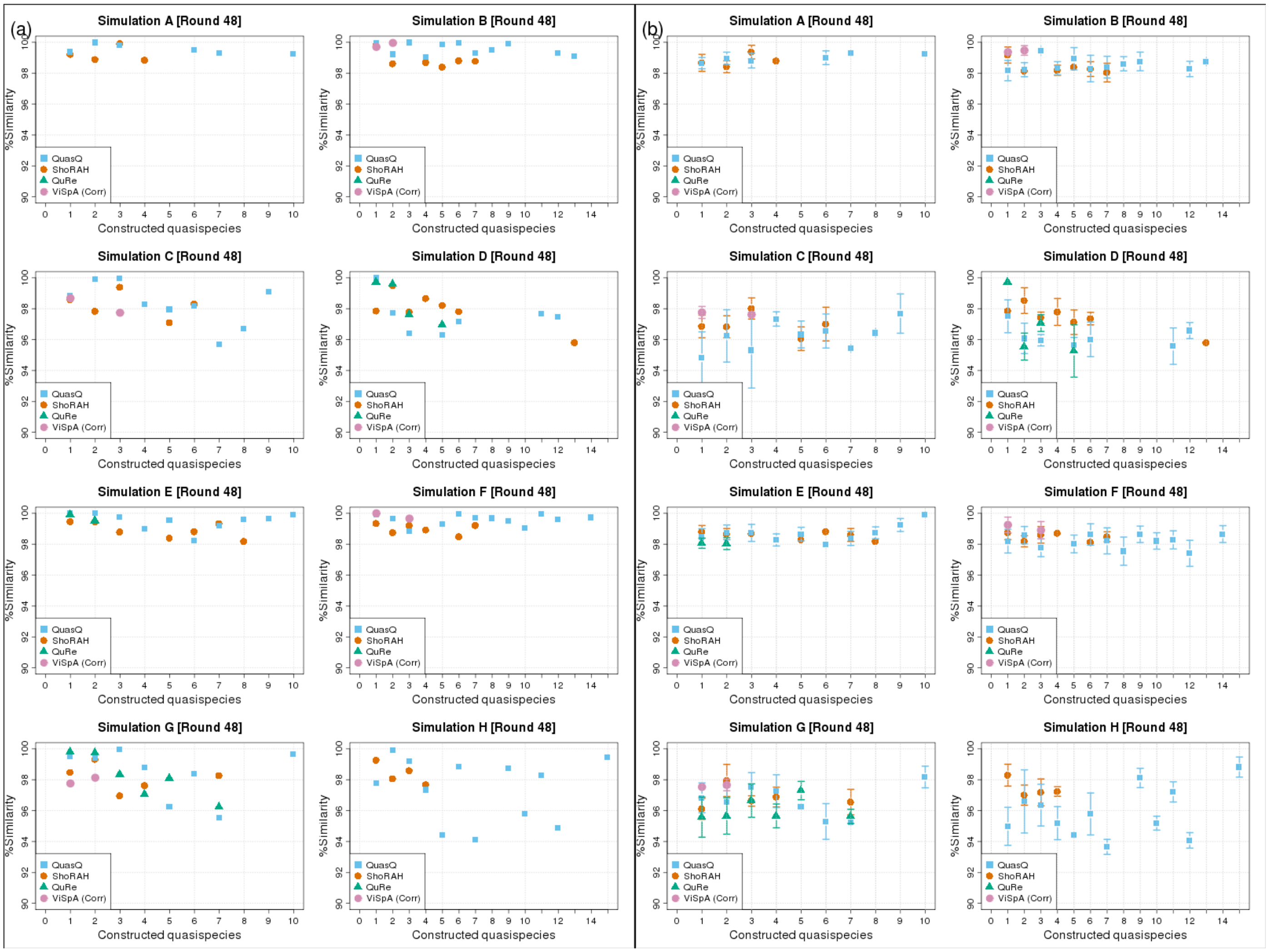

Supplementary Figure 52. (a) Degree of similarity between the best reconstructed haplotype sequences from QuasQ (blue squares), ShoRAH (red diamonds), QuRe (green triangles) and ViSpA with ShoRAH corrected reads (pink circle) with the actual simulated sequences. Here we identified the most similar reconstructed sequence to the simulated variants. (b) This shows the mean and standard deviation of the similarity for the reconstructed sequences that are mapped to a particular simulated variant. The above figures are based on the data from the 48th round of the simulations.

Supplementary Figure 53.

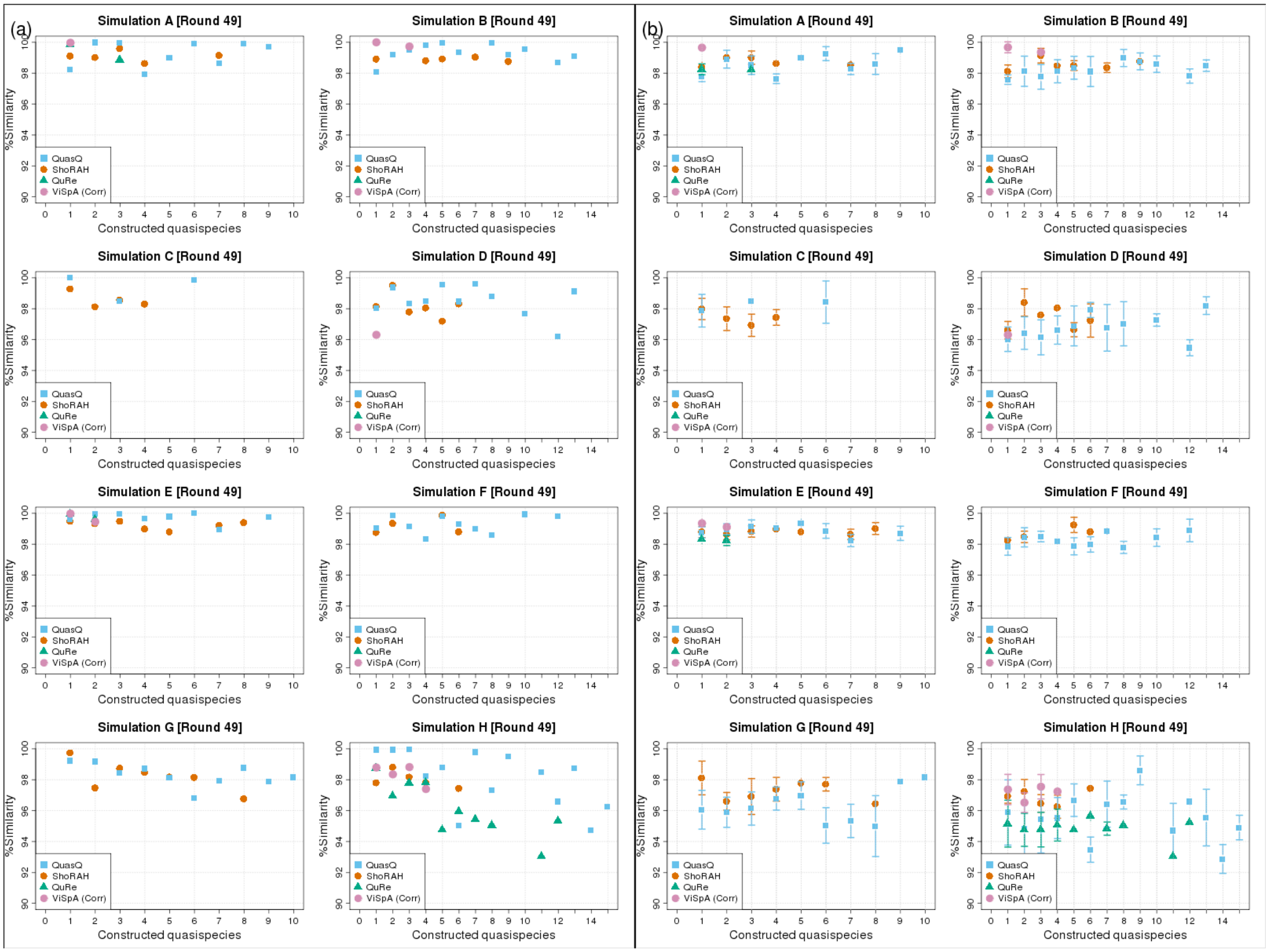

Supplementary Figure 53. (a) Degree of similarity between the best reconstructed haplotype sequences from QuasQ (blue squares), ShoRAH (red diamonds), QuRe (green triangles) and ViSpA with ShoRAH corrected reads (pink circle) with the actual simulated sequences. Here we identified the most similar reconstructed sequence to the simulated variants. (b) This shows the mean and standard deviation of the similarity for the reconstructed sequences that are mapped to a particular simulated variant. The above figures are based on the data from the 49th round of the simulations.

Supplementary Figure 54.

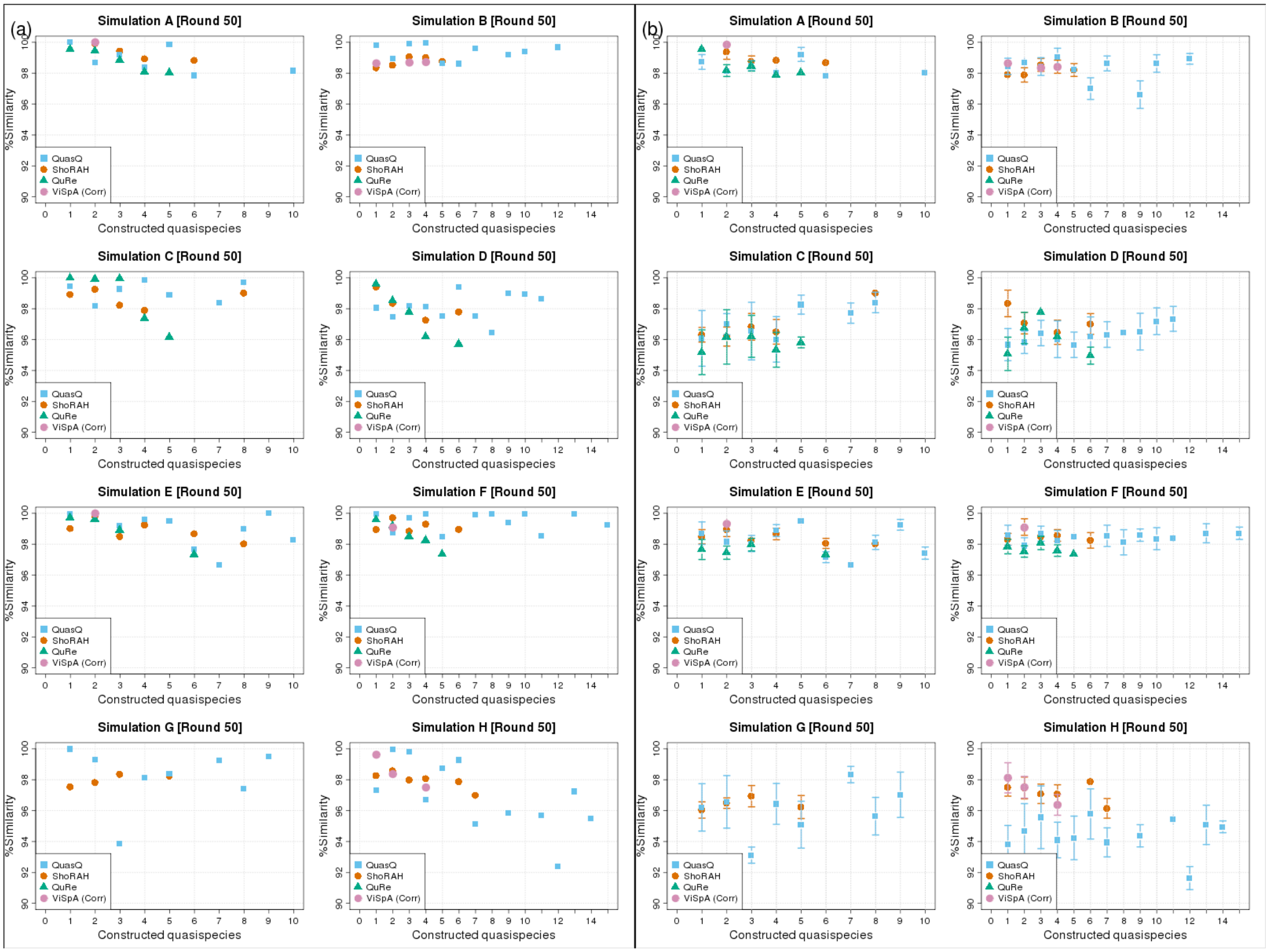

Supplementary Figure 54. (a) Degree of similarity between the best reconstructed haplotype sequences from QuasQ (blue squares), ShoRAH (red diamonds), QuRe (green triangles) and ViSpA with ShoRAH corrected reads (pink circle) with the actual simulated sequences. Here we identified the most similar reconstructed sequence to the simulated variants. (b) This shows the mean and standard deviation of the similarity for the reconstructed sequences that are mapped to a particular simulated variant. The above figures are based on the data from the 50th round of the simulations.

Supplementary Figure 55.

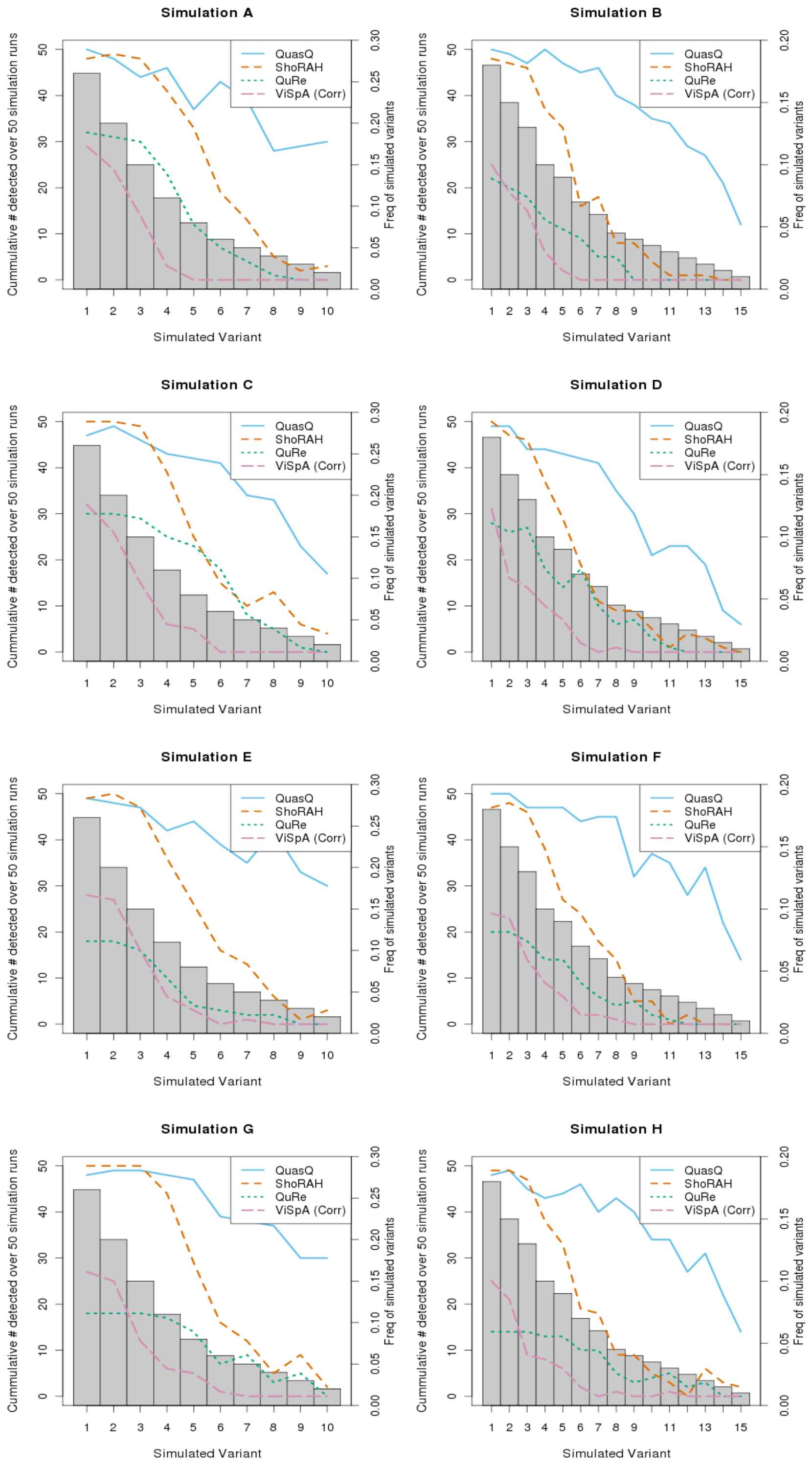

Supplementary Figure 55. The detected simulated variants (out of a total of 15) for each of the 50 runs of simulations with type B setting (Table 1) were tabulated. This figure plots the cumulative count of each of the simulated variants constructed over the 50 runs of simulation B for QuasQ (blue), ShoRAH (red), QuRe (green) and ViSpA with ShoRAH corrected reads (pink) as described by the left vertical axis. The grey bars show the respective frequencies of the simulated variants as described by the right vertical axis.

Supplementary Figure 56.

(a)

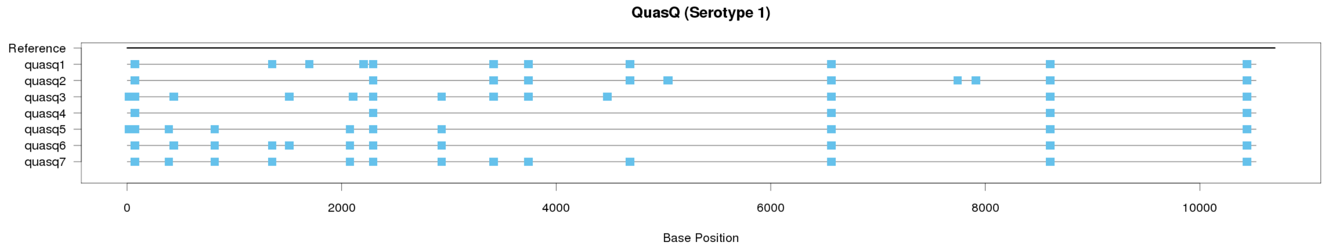

(b)

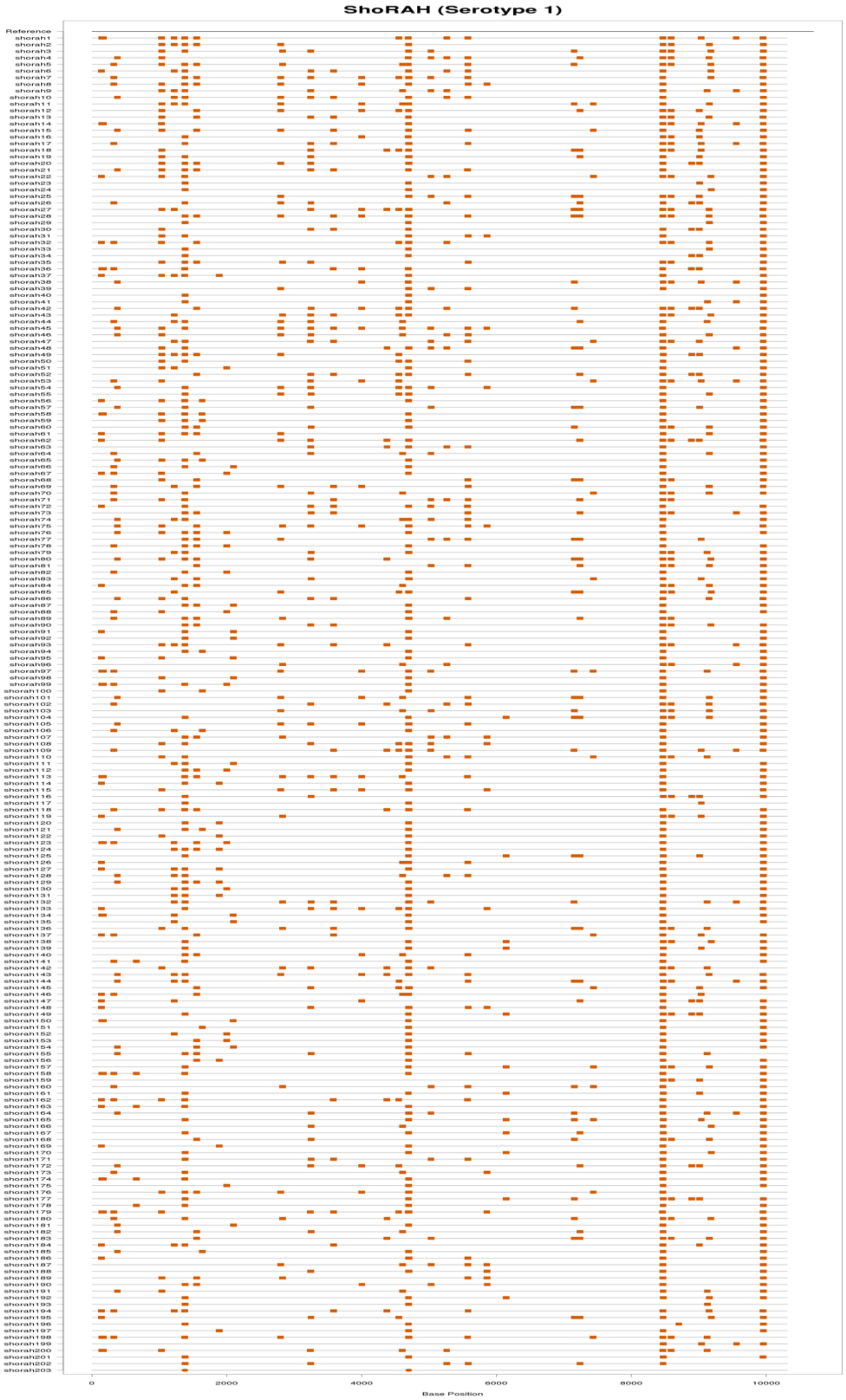

(c)

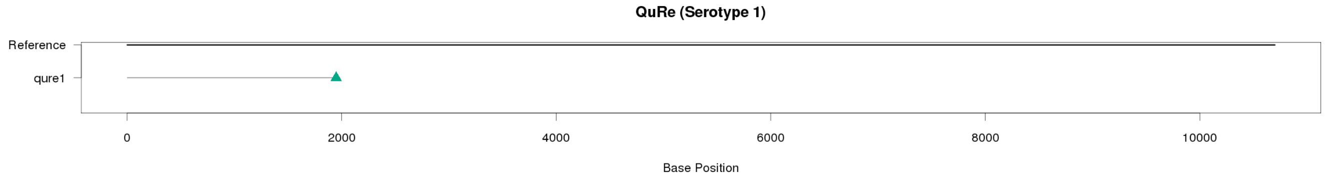
QuRe (Serotype 1)
Reference
qure1
0
2000
4000
6000
8000
10000
Base Position

(d)

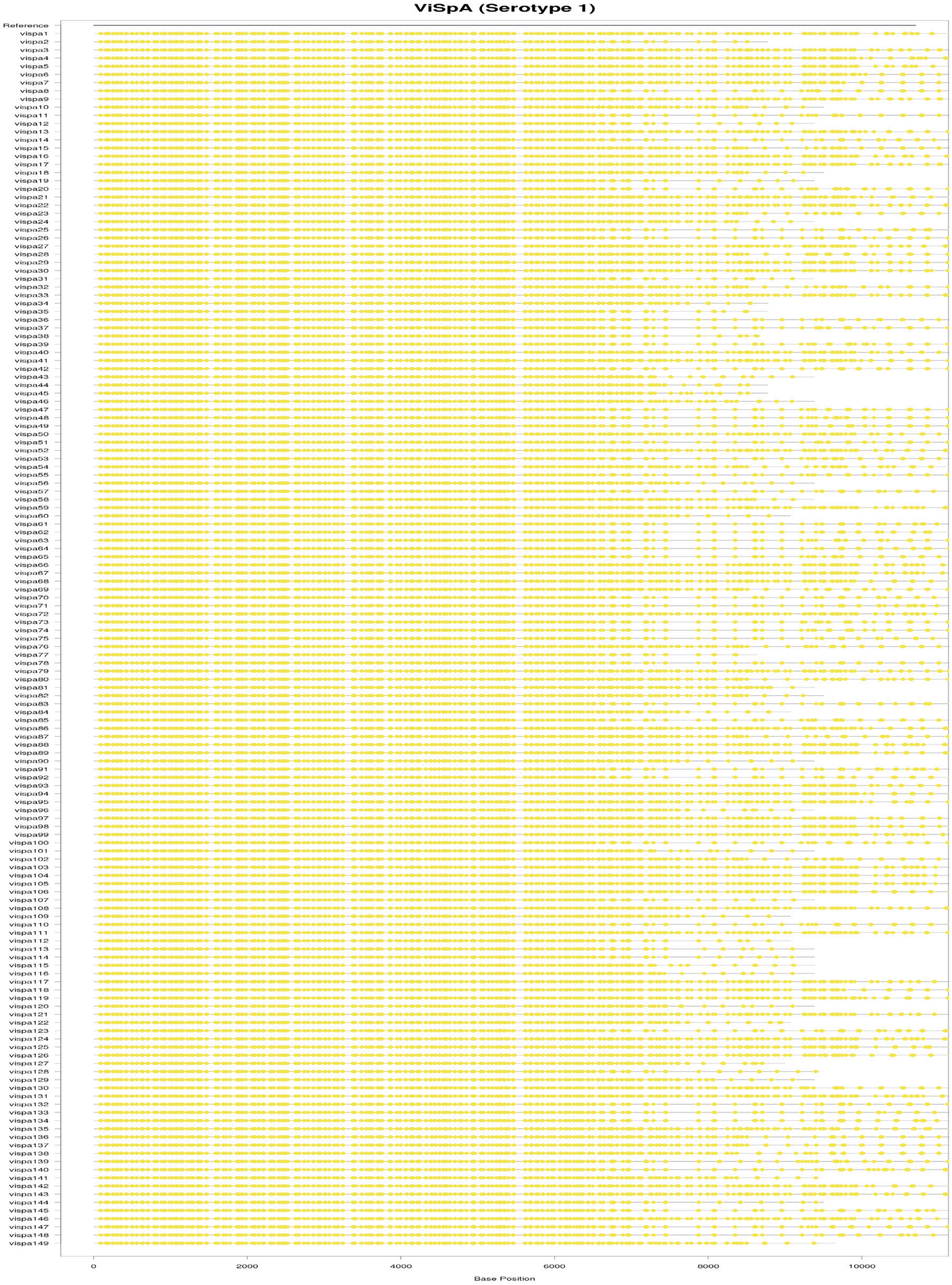
ViSpA (Serotype 1)
Reference
vispa1
vispa2
vispa3
vispa4
vispa5
vispa6
vispa7
vispa8
vispa9
vispa10
vispa11
vispa12
vispa13
vispa14
vispa15
vispa16
vispa17
vispa18
vispa19
vispa20
vispa21
vispa22
vispa23
vispa24
vispa25
vispa26
vispa27
vispa28
vispa29
vispa30
vispa31
vispa32
vispa33
vispa34
vispa35
vispa36
vispa37
vispa38
vispa39
vispa40
vispa41
vispa42
vispa43
vispa44
vispa45
vispa46
vispa47
vispa48
vispa49
vispa50
vispa51
vispa52
vispa53
vispa54
vispa55
vispa56
vispa57
vispa58
vispa59
vispa60
vispa61
vispa62
vispa63
vispa64
vispa65
vispa66
vispa67
vispa68
vispa69
vispa70
vispa71
vispa72
vispa73
vispa74
vispa75
vispa76
vispa77
vispa78
vispa79
vispa80
vispa81
vispa82
vispa83
vispa84
vispa85
vispa86
vispa87
vispa88
vispa89
vispa90
vispa91
vispa92
vispa93
vispa94
vispa95
vispa96
vispa97
vispa98
vispa99
vispa100
vispa101
vispa102
vispa103
vispa104
vispa105
vispa106
vispa107
vispa108
vispa109
vispa110
vispa111
vispa112
vispa113
vispa114
vispa115
vispa116
vispa117
vispa118
vispa119
vispa120
vispa121
vispa122
vispa123
vispa124
vispa125
vispa126
vispa127
vispa128
vispa129
vispa130
vispa131
vispa132
vispa133
vispa134
vispa135
vispa136
vispa137
vispa138
vispa139
vispa140
vispa141
vispa142
vispa143
vispa144
vispa145
vispa146
vispa147
vispa148
vispa149
0
2000
4000
6000
8000
10000
Base Position

(e)

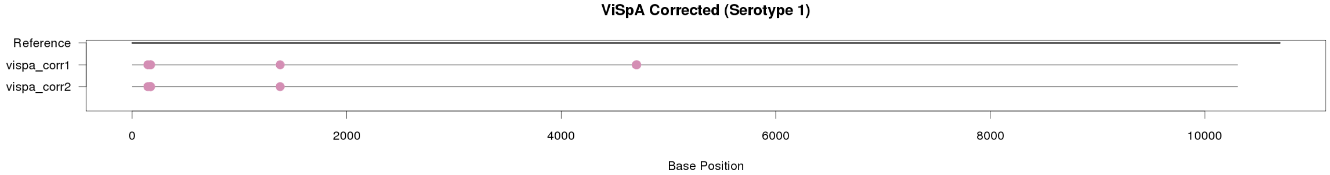

Supplementary Figure 56. BLASTn identity of the top scoring alignment of each variant constructed for serotype 1 isolates by (a) QuasQ (b) ShoRAH (c) QuRe (d) ViSpA and (e) ViSpA (with ShoRAH corrected reads). Due to the large number of variants constructed by ViSpA, we illustrated only those variants with ≥80% identity and align to ≥ 80% of the reference genome length.

Supplementary Figure 57.

(a)

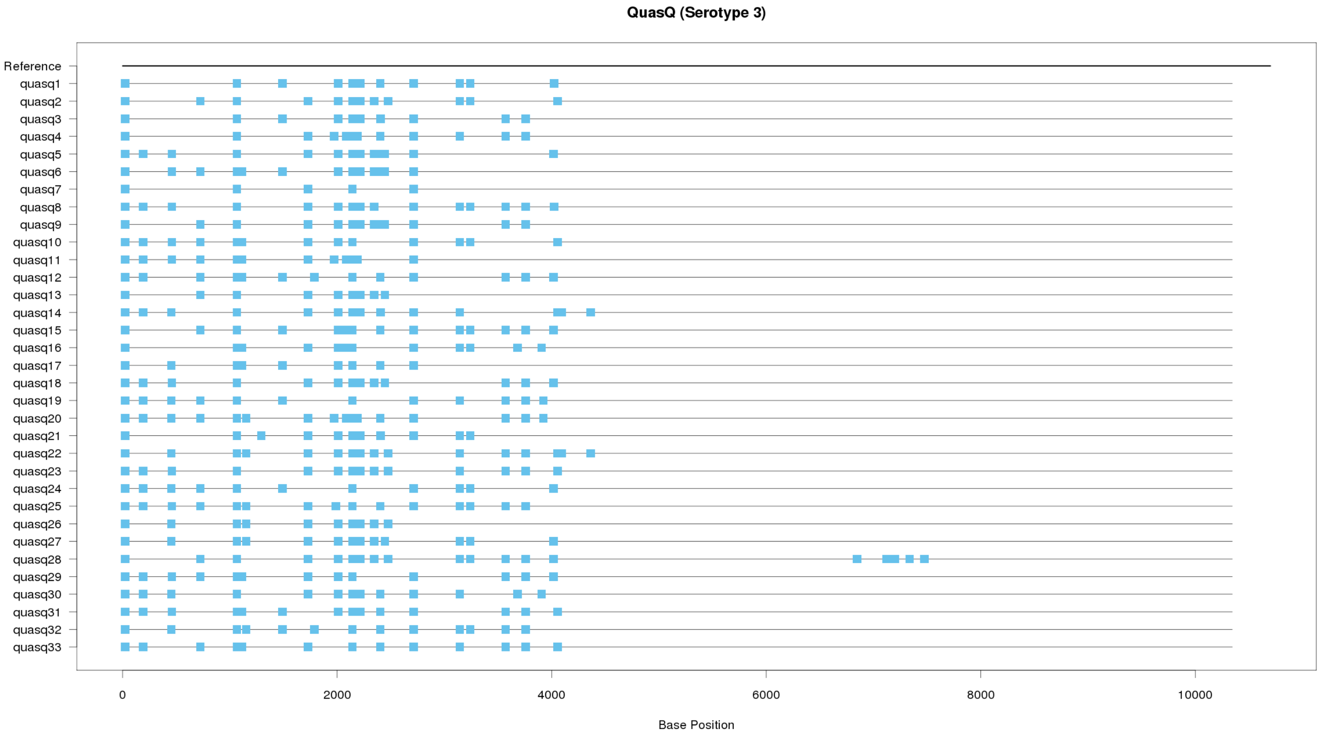

(b)

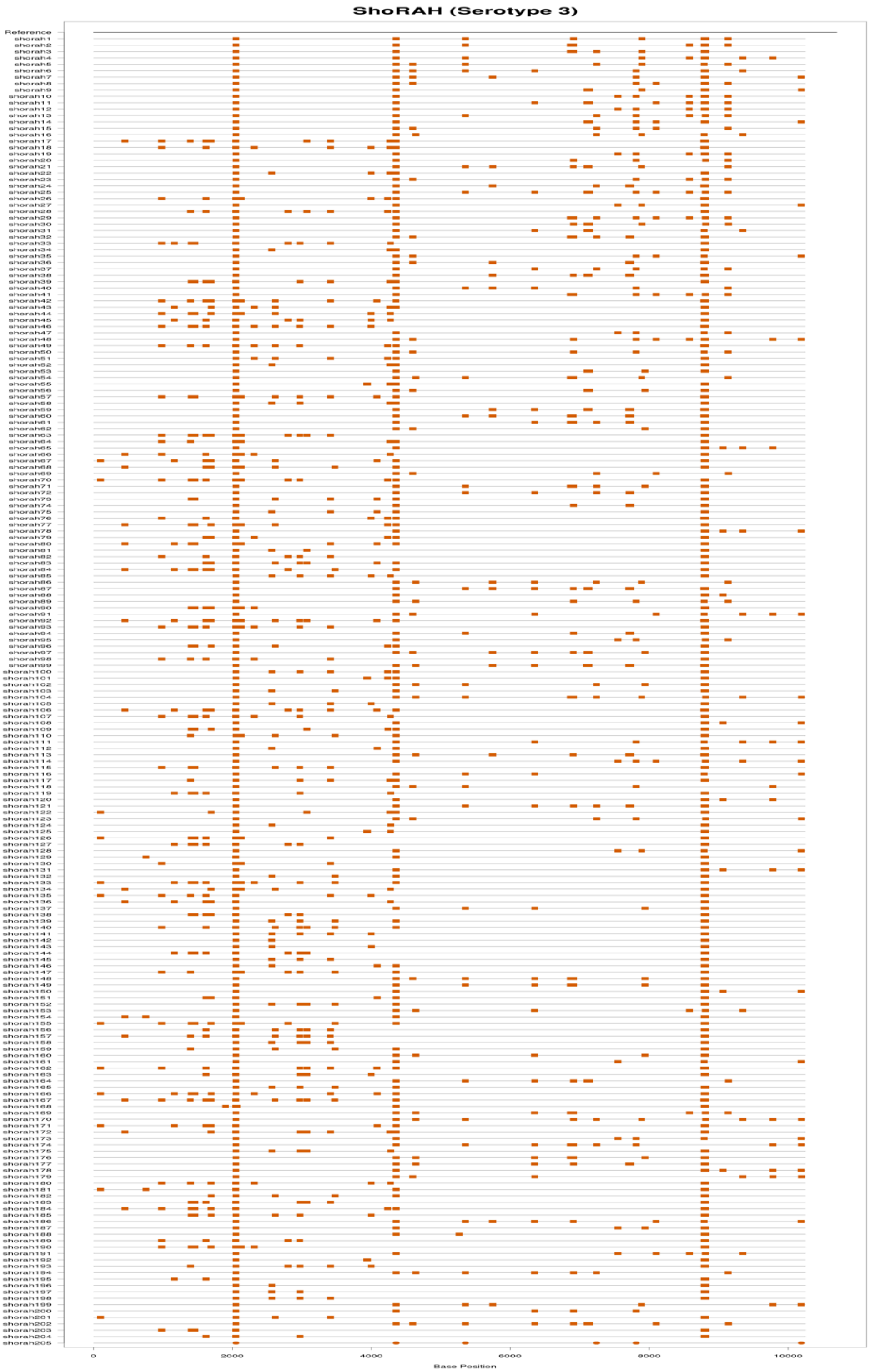
ShoRAH (Serotype 3)
Reference
0
2000
4000
6000
8000
10000
Base Position

(c)

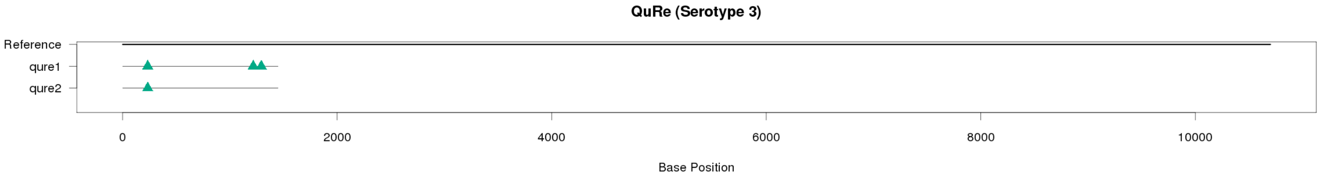
QuRe (Serotype 3)
Reference
qure1
qure2
0
2000
4000
6000
8000
10000
Base Position

(d)

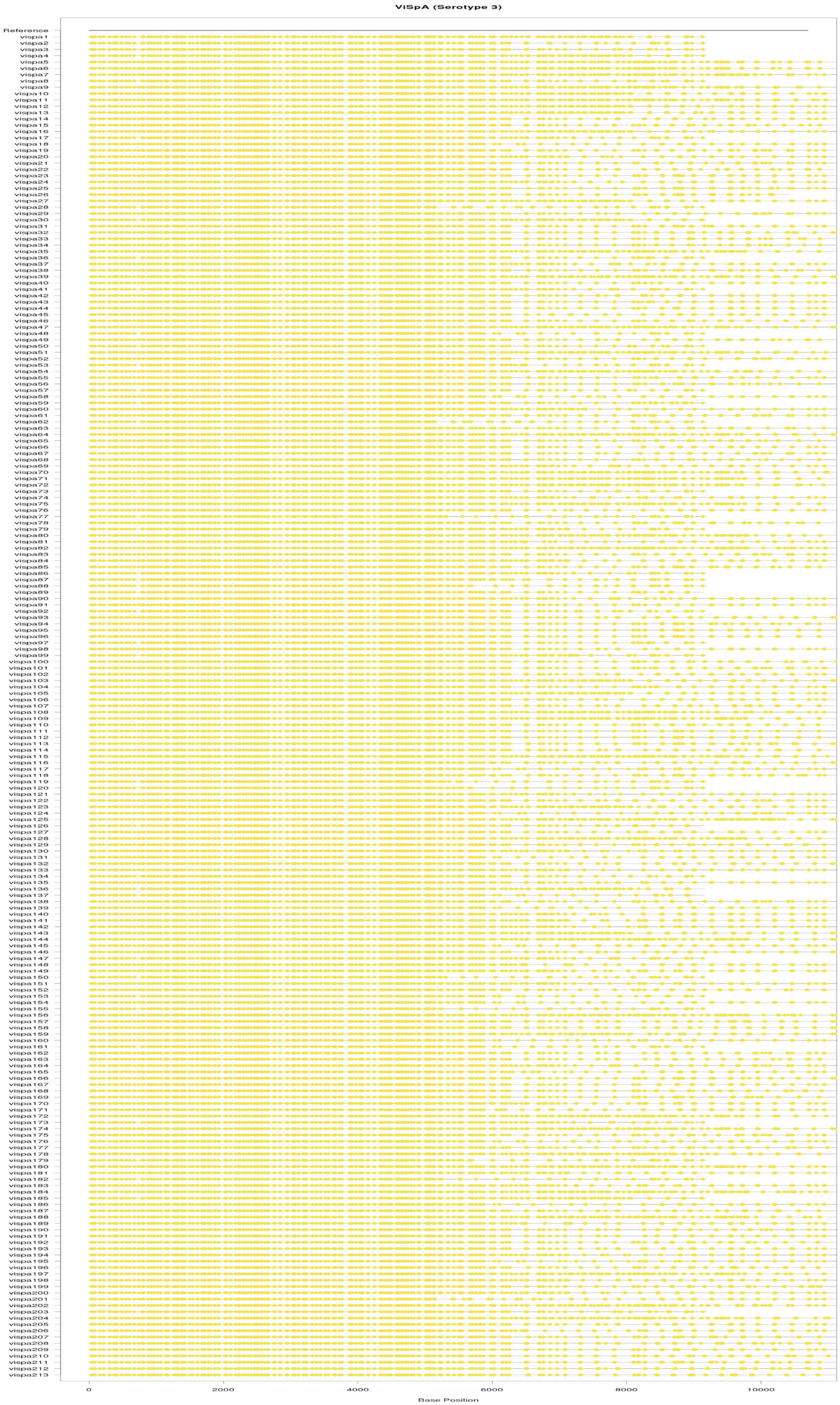

ViSpA (Serotype 3)
Reference
Base Position
0
2000
4000
6000
8000
10000

(e)

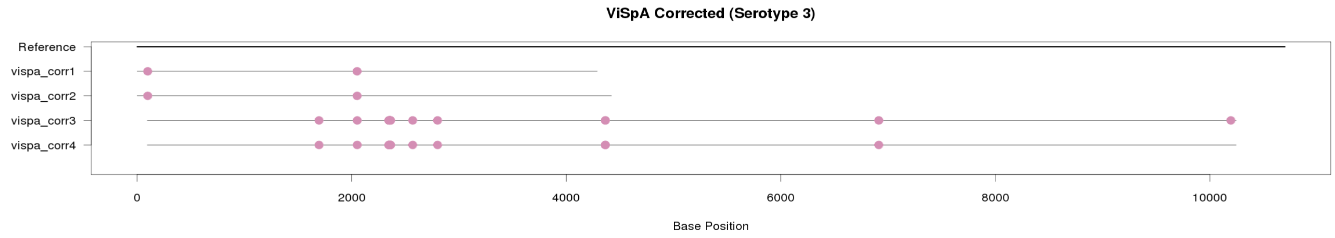

Supplementary Figure 57. BLASTn identity of the top scoring alignment of each variant constructed for serotype 3 isolates by (a) QuasQ (b) ShoRAH (c) QuRe (d) ViSpA and (e) ViSpA (with ShoRAH corrected reads). Due to the large number of variants constructed by ViSpA, we illustrated only those variants with ≥80% identity and align to ≥ 80% of the reference genome length.

Supplementary Figure 58.

(a)

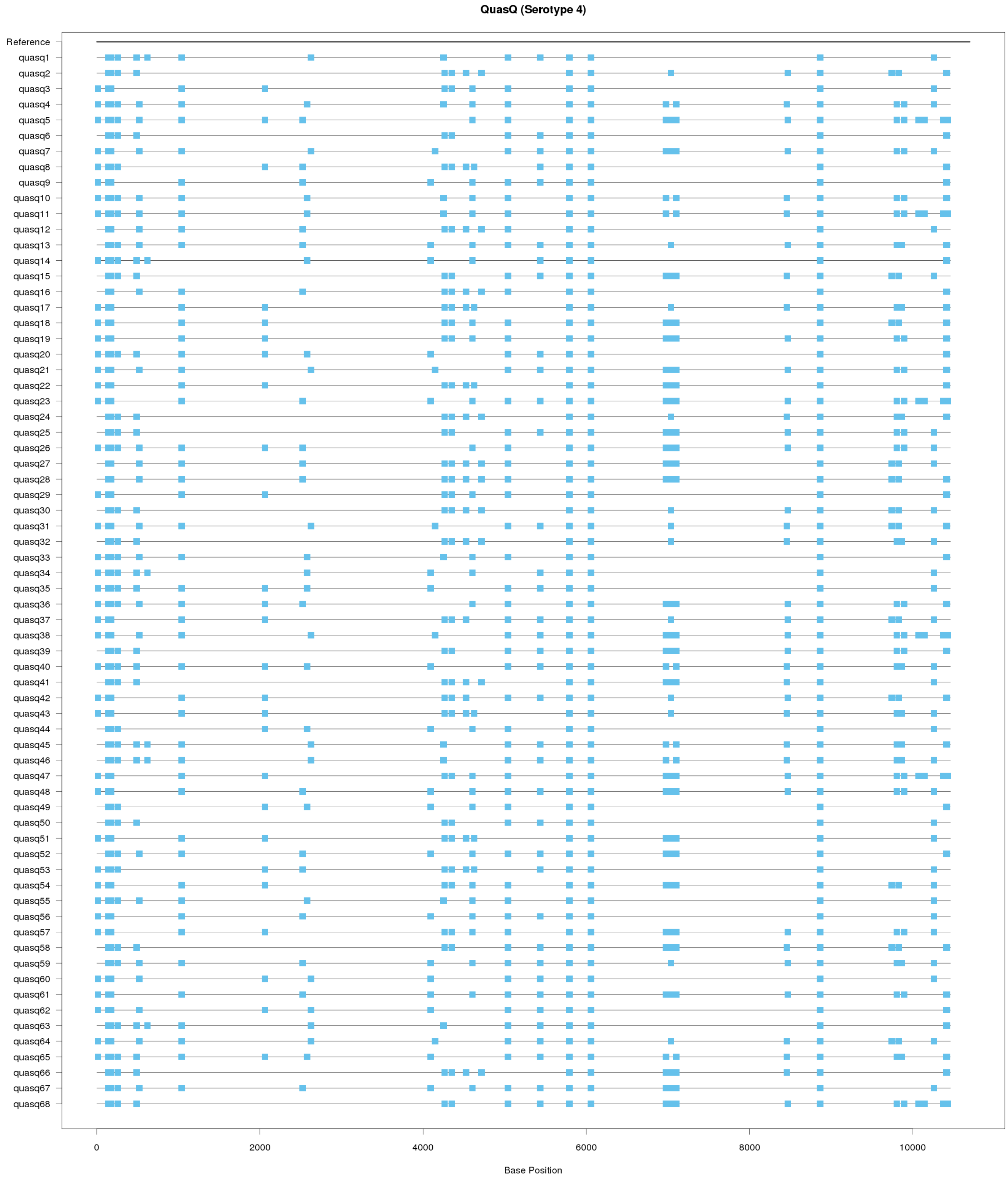

(b)

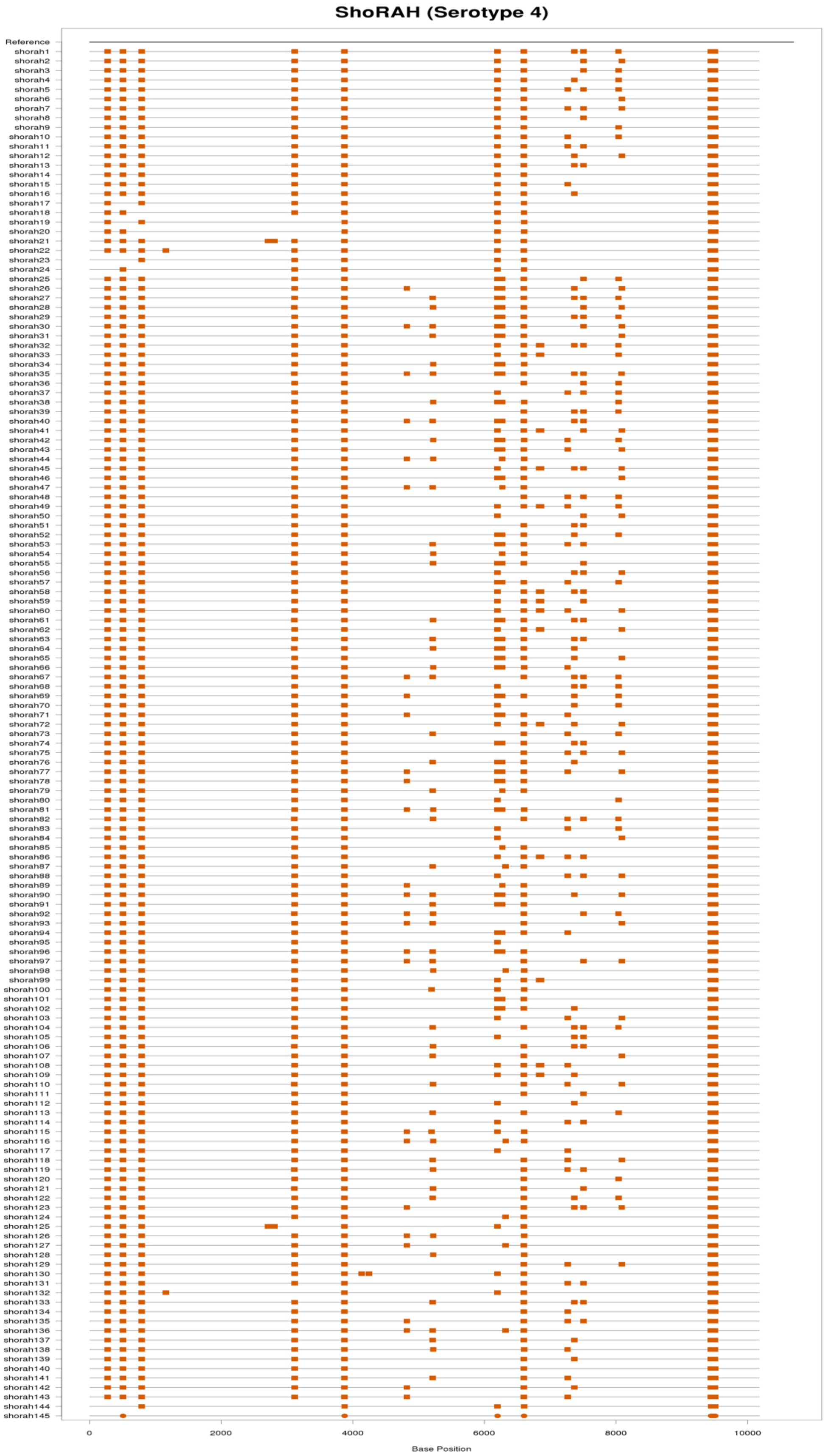
ShoRAH (Serotype 4)
Reference
Base Position
0
2000
4000
6000
8000
10000

(c)

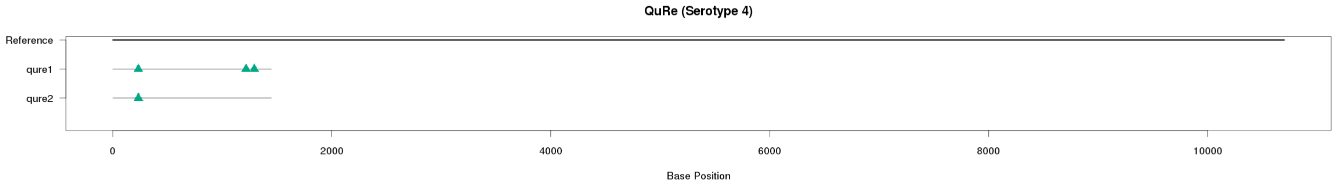

(d)

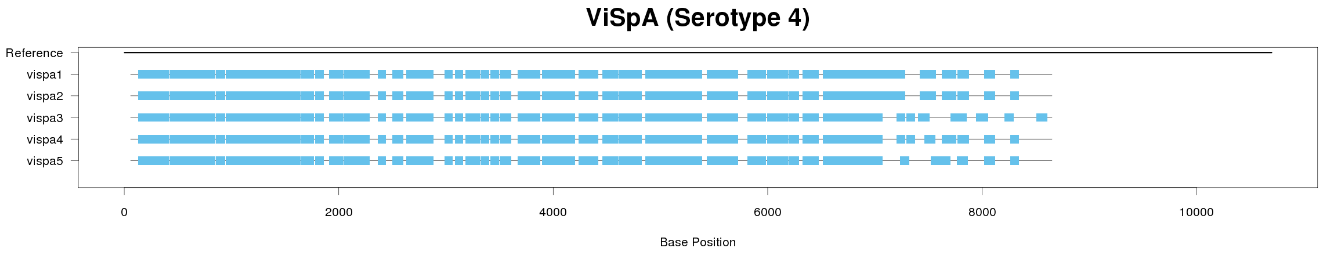

(e)

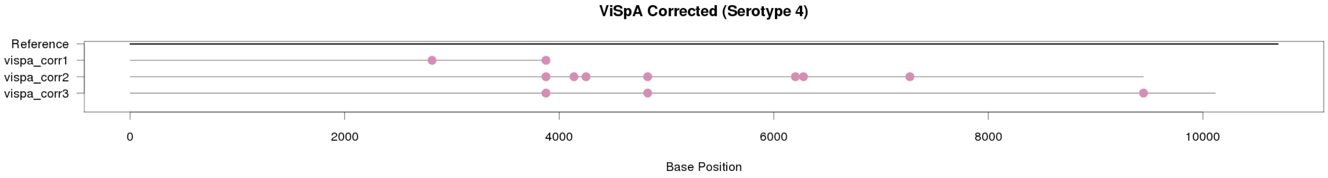

Supplementary Figure 58. BLASTn identity of the top scoring alignment of each variant constructed for serotype 4 isolates by (a) QuasQ (b) ShoRAH (c) QuRe (d) ViSpA and (e) ViSpA (with ShoRAH corrected reads). Due to the large number of variants constructed by ViSpA, we illustrated only those variants with ≥80% identity and align to ≥ 80% of the reference genome length.

Supplementary Table 1. Summary of mean number of constructed sequences, mean recall rate, mean precision and mean F-measure for QuasiRecomb, with and without recombination, over the 50 runs of Simulation A.

	No. of constructed sequences	*Recall Rate*	*Precision*	*F-measure*
With Recombination	9999.420	0.294	0.0003	0.0006
Without Recombination	9963.120	0.242	0.0002	0.0005
